# Assessment of adverse effects attributed to statin therapy in product labels: a meta-analysis of double-blind randomised controlled trials

**DOI:** 10.1016/S0140-6736(25)01578-8

**Published:** 2026-02-05

**Authors:** Cholesterol Treatment Trialists’ (CTT) Collaboration, Writing Committee, Christina Reith, Lisa Blackwell, Jonathan R Emberson, David Preiss, Enti Spata, Kelly Davies, Heather Halls, Charlie Harper, Lisa Holland, Kate Wilson, Robert Humphrey, Alistair J Roddick, Christopher P Cannon, Barry R Davis, Paul N Durrington, Shinya Goto, Graham A Hitman, G Kees Hovingh, Wolfgang Koenig, Vera Krane, Martin J Landray, Borislava Mihaylova, Connie Newman, Jeffrey L Probstfield, Marc S Sabatine, Naveed Sattar, Gregory G Schwartz, Andrew M Tonkin, Harvey D White, Jane Armitage, Anthony Keech, John Simes, Rory Collins, Colin Baigent, CTT Secretariat, Jane Armitage, Colin Baigent, Elizabeth Barnes, Lisa Blackwell, Rory Collins, Kelly Davies, Jonathan Emberson, Jordan Fulcher, Heather Halls, William G Herrington, Lisa Holland, Anthony Keech, Adrienne Kirby, Borislava Mihaylova, Rachel O’Connell, David Preiss, Christina Reith, John Simes, Kate Wilson, CTT collaborating trialists, A to Z trial (phase Z), Michael Blazing, Eugene Braunwald, James de Lemos, Sabina Murphy, Terje R Pedersen, Marc Pfeffer, Harvey D White, Stephen Wiviott, AirForce–Texas Coronary Atherosclerosis Prevention Study (AFCAPS/TexCAPS), Michael Clearfield, John R Downs, Antonio Gotto, Stephen Weis, Assessment of Lescol in Renal Transplantation trial (ALERT), Bengt Fellström, Hallvard Holdaas, Alan Jardine, Terje R Pedersen, Antihypertensive and Lipid-Lowering Treatment to Prevent Heart Attack Trial (ALLHAT), David Gordon, Barry R Davis, Curt Furberg, Richard Grimm, Sara Pressel, Jeffrey L Probstfield, Mahboob Rahman, Lara Simpson, Aggressive Lipid-Lowering Initiation Abates New Cardiac Events trial (ALLIANCE), Michael Koren, Anglo-Scandinavian Cardiac Outcomes Trial (ASCOT), Björn Dahlöf, Ajay Gupta, Neil Poulter, Peter Sever, Hans Wedel, Atorvastatin Study for Prevention of Coronary Heart Disease Endpoints in Non-Insulin-Dependent Diabetes Mellitus study (ASPEN), Robert H Knopp, A Study to Evaluate the Use of Rosuvastatin in Subjects On Regular Haemodialysis: an Assessment of Survival and Cardiovascular Events (AURORA), Stuart Cobbe, Bengt Fellström, Hallvard Holdaas, Alan Jardine, Roland Schmieder, Faiez Zannad, Collaborative Atorvastatin Diabetes Study (CARDS), D John Betteridge, Helen M Colhoun, Paul N Durrington, John Fuller, Graham A Hitman, Andrew Neil, Cholesterol And Recurrent Events trial (CARE), Eugene Braunwald, Barry R Davis, C Morton Hawkins, Lemuel Moyé, Marc Pfeffer, Frank Sacks, Controlled Rosuvastatin Multinational Trial in Heart Failure (CORONA), John Kjekshus, Hans Wedel, John Wikstrand, Die Deutsche Diabetes Dialyse Studie (4D), Christoph Wanner, Vera Krane, Gruppo Italiano per lo Studio della Sopravvivenza nell’Infarto miocardico Heart Failure and Prevention trials (GISSI), Maria Grazia Franzosi, Roberto Latini, Donata Lucci, Aldo Maggioni, Roberto Marchioli, Enrico B Nicolis, Luigi Tavazzi, Gianni Tognoni, Heart Outcomes Prevention Evaluation-3 trial (HOPE-3), Jackie Bosch, Eva Lonn, Salim Yusuf, Heart Protection Study (HPS), Jane Armitage, Louise Bowman, Rory Collins, Anthony Keech, Martin J Landray, Sarah Parish, Richard Peto, Peter Sleight, Incremental Decrease in Endpoints through Aggressive Lipid-lowering study (IDEAL), John JP Kastelein, Terje R Pedersen, Justification for the Use of Statins in Prevention: an Intervention Trial Evaluating Rosuvastatin (JUPITER), Robert Glynn, Antonio Gotto, John JP Kastelein, Wolfgang Koenig, Jean MacFadyen, Paul M Ridker, Long-term Intervention with Pravastatin in Ischaemic Disease trial (LIPID), Anthony Keech, Stephen MacMahon, Ian Marschner, Andrew M Tonkin, John Shaw, John Simes, Harvey D White, Lescol Intervention Prevention Study (LIPS): Patrick W Serruys; Post-Coronary Artery Bypass Graft trial (POST-CABG), Genell Knatterud, Prospective Study of Pravastatin in the Elderly at Risk (PROSPER), Gerard J Blauw, Stuart Cobbe, Ian Ford, J Wouter Jukema, Peter Macfarlane, Chris Packard, Naveed Sattar, James Shepherd, Stella Trompet, Pravastatin or Atorvastatin Evaluation and Infection Therapy trial (PROVE-IT), Eugene Braunwald, Christopher P Cannon, Sabina Murphy, Study of the Effectiveness of Additional Reductions in Cholesterol and Homocysteine (SEARCH), Rory Collins, Jane Armitage, Louise Bowman, Richard Bulbulia, Richard Haynes, Sarah Parish, Richard Peto, Peter Sleight, Stroke Prevention by Aggressive Reduction in Cholesterol Levels study (SPARCL), Pierre Amarenco, K Michael Welch, Scandinavian Simvastatin Survival Study (4S), John Kjekshus, Terje R Pedersen, Lars Wilhelmsen, Treating to New Targets study (TNT), Philip Barter, Antonio Gotto, John LaRosa, John JP Kastelein, James Shepherd, West of Scotland Coronary Prevention Study (WOSCOPS), Stuart Cobbe, Ian Ford, Sharon Kean, Peter Macfarlane, Chris Packard, Michele Robertson, Naveed Sattar, James Shepherd, Robin Young, Other CTT members, Hiroyuki Arashi, Robert Clarke, Marcus Flather, Shinya Goto, Uri Goldbourt, Jemma Hopewell, Patricia Kearney, G Kees Hovingh, George Kitas, Connie Newman, Marc S Sabatine, Gregory G Schwartz, Liam Smeeth, Jonathan Tobert, John Varigos, Junichi Yamaguchi, Independent Oversight Committee, Emily Banks, Michael Blastland, Stephen Evans, Robert Temple, Peter Weissberg, Janet Wittes

## Abstract

**Background:**

Statin product labels (eg, Summaries of Product Characteristics [SmPCs]) list certain adverse outcomes as potential treatment-related effects based mainly on non-randomised and non-blinded studies, which might be subject to bias. We aimed to assess the evidence for such undesirable effects more reliably through a meta-analysis of individual participant data from large double-blind trials of statin therapy.

**Methods:**

In this meta-analysis of individual participant-level data from double-blind randomised controlled trials, we generated a list of all undesirable effect terms listed in statin SmPCs by searching an electronic medicines compendium for five statins (atorvastatin, fluvastatin, pravastatin, rosuvastatin, and simvastatin). Randomised trials were eligible for meta-analysis of these effects if they involved at least 1000 participants, had a scheduled treatment period of at least 2 years, and involved a double-blind comparison of statin versus placebo or of a more intensive versus a less intensive statin regimen. Event rate ratios (RRs) and 95% CIs were calculated with statistical significance assessed after controlling the false discovery rate (FDR) at 5%.

**Findings:**

19 trials compared statin versus placebo (123 940 participants, median follow-up 4·5 years [IQR 3·1–5·4]). In addition to previously reported effects on muscle outcomes and diabetes, only four of 66 further undesirable outcomes that had been attributed to statins were FDR significant: abnormal liver transaminases (783 participants [0·30% per annum] allocated statin *vs* 556 [0·22% per annum] allocated placebo, RR 1·41 [95% CI 1·26–1·57]) and other liver function test abnormalities (651 participants [0·25% per annum] allocated statin *vs* 518 [0·20% per annum] allocated placebo, RR 1·26 [1·12–1·41]; absolute annual excess of 0·13% for combined liver function test abnormality), urinary composition alteration (556 [0·21% per annum] allocated statin *vs* 472 [0·18% per annum] allocated placebo, RR 1·18 [1·04–1·33]), and oedema (3495 [1·38% per annum] allocated statin *vs* 3299 [1·31% per annum] allocated placebo, RR 1·07 [1·02–1·12]). Analysis of the four trials of more intensive versus less intensive statin regimens also found significant excesses for abnormal liver transaminases and other liver function test abnormalities (supporting a dose-dependent effect), but no significant excess was found for urinary composition alteration or oedema.

**Interpretation:**

Adverse event data from blinded randomised trials do not support causal relationships between statin therapy and most of the conditions (including cognitive impairment, depression, sleep disturbance, and peripheral neuropathy) listed in product labels as potential undesirable effects. In light of these findings, such labelling and other official sources of health information should be revised so that patients and their doctors can make appropriately informed decisions regarding statin therapy.

**Funding:**

British Heart Foundation, UK Medical Research Council, and Australian National Health and Medical Research Council.

## Introduction

Large, randomised, placebo-controlled trials have shown that statin therapy reduces the risk of major vascular events in a wide range of people,^1–5^ and has no detectable effects on non-vascular mortality^1^ or on cancer.^6^ An effective statin regimen (eg, atorvastatin 40 mg per day) for 5 years in 10 000 patients would typically prevent major vascular events from occurring in about 1000 patients (ie, 10% absolute benefit) with pre-existing occlusive vascular disease (secondary prevention) and in 500 patients (ie, 5% absolute benefit) who are at increased risk but have not yet had a vascular event (primary prevention).^7^ The main established adverse effect of statin therapy is myopathy, which occurs in rare cases (approximately one case per 10 000 person-years), or, in a more severe form, rhabdomyolysis (approximately 2–3 cases per 100 000 person-years), as indicated by muscle symptoms and related biochemical changes (eg, multi-fold rises in creatine kinase concentrations).^7,8^ In addition, statin therapy causes a small absolute increase (about 1%) in less severe muscle symptoms, although this excess is largely confined to the first year of treatment.^9^ Statins are also associated with a moderate dose-dependent increase in new diagnoses of diabetes, with the majority of cases occurring in people with glycaemic markers already close to the diagnostic threshold for diabetes before initiation of treatment.^10^ Overall, the expert clinical consensus is that the proven cardiovascular benefits of statins far outweigh their known risks and, as a consequence, statins are now prescribed to millions of people worldwide.

However, in non-randomised and non-blinded observational studies (eg, post-marketing safety surveillance data or individual case reports), statin therapy has been associated with an increased risk of several other adverse effects, such as hepatic dysfunction,^11–13^ depression,^14^ impaired cognition,^15^ sleep disturbance,^16^ acute kidney injury, or renal failure,^11,17^ interstitial lung disease,^18^ and pancreatitis.^19^ Although such pharmacoepidemiological studies can be of value in detecting large adverse effects of treatment on rare health outcomes (ie, that would not normally be expected to occur in those not exposed to the intervention), they cannot be relied on for assessment of the causal nature of treatment-related associations when the relative risks are moderate, or relate to health outcomes that are common in the types of patients being studied, because of their inherent potential biases.^7^ Despite this limitation, these observations have resulted in a number of possible undesirable effects of treatment being listed in statin product information labels. Unreliable information about adverse effects of statin therapy hampers patients’ and clinicians’ ability to make properly informed decisions regarding the balance of benefits and risks, and might lead to people who would benefit from taking a statin not starting treatment, or stopping it prematurely,^20–22^ with potentially life-threatening health consequences.

Large-scale randomised controlled trials and their associated meta-analyses can minimise both moderate systematic and random errors in the assessment of treatment effects. In this Article, we used extensive individual participant data from the Cholesterol Treatment Trialists’ (CTT) Collaboration to test whether the potential undesirable effects listed in statin product labels are causally related to statin therapy.

## Methods

### Search strategy and selection criteria

The CTT Collaboration prospectively described its plan to reliably ascertain all statin effects.^23^ Methods have been described in detail.^24^ Briefly, we conducted a meta-analysis of individual participant data from randomised controlled trials of statin therapy participating in the CTT Collaboration. To eliminate reporting biases, which are especially probable when possible adverse outcomes are already listed in product labels given to patients, we restricted our analyses to trials with a double-blind design. Trials were eligible for inclusion if there were no protocol-mandated differences between treatment groups other than those created by blinded allocation to statin versus placebo or blinded allocation to more intensive statin therapy versus less intensive statin therapy, they involved 1000 or more participants, and they included a scheduled treatment period of at least 2 years. We undertook a new round of data collection and processing to collect individual participant data related to all adverse events (ie, any untoward medical occurrences in the trial participants, regardless of whether believed to be causally related to statin therapy) recorded during the scheduled period of treatment and follow-up in these trials.^23^

### Outcome classification

Data were converted into a common format on the basis of the Clinical Data Interchange Standards Consortium Study Data Tabulation Model,^25^ with adverse events being mapped to a common dictionary (the Medical Dictionary for Regulatory Activities^26^ [MedDRA] version 20.0), blind to treatment allocation. Adverse events pertaining to laboratory abnormalities were derived solely from reported events as opposed to interrogation of any biochemical data.

There are hundreds of statin product labels available for statin therapy, with each statin type, dose, and formulation being afforded a designated Summary of Product Characteristics (SmPC). In each SmPC, there is a section that lists outcomes classed as potential undesirable effects. These effects typically include terms related to muscle and diabetes-related outcomes, which have previously been assessed by the CTT Collaboration.^9,10^ A list of all other undesirable effect terms listed in statin SmPCs to be tested in the CTT database was created by searching an electronic medicines compendium^27^ up to March 9, 2023, for each of the five currently widely used types of statin (ie, atorvastatin, rosuvastatin, simvastatin, pravastatin, and fluvastatin; note there is no lovastatin SmPC available in this compendium, likely reflecting its relatively limited use in current clinical practice). For each of these statin types, at least two researchers reviewed statin SmPCs for at least one low-intensity or moderate-intensity statin and one high-intensity formulation, with all terms listed under SmPC section 4.8 as undesirable effects being evaluated. These terms were consolidated into a single list, duplicates were removed, and the terms were then mapped to MedDRA version 20.0 at the preferred term level. When no direct match was found, a clinician mapped the term to the closest available MedDRA preferred term. In this way, undesirable effects cited in one or more SmPC documents were expressed as MedDRA preferred terms and categorised into appropriate body systems. Certain undesirable effect outcomes were combined into a single outcome (eg, nausea and vomiting are listed separately as undesirable effects in statin SmPCs, but were merged into one outcome). For some of these outcomes, further preferred terms assessed as closely medically related to direct-match preferred terms were included in analyses. However, if a medically related term clearly indicated an alternative cause for an outcome other than drug therapy, such as an infective or traumatic cause or a genetic or hereditary basis (eg, the preferred terms post-procedural diarrhoea, traumatic arthritis, or congenital anaemia), the terms were not included. After merging similar terms into composite outcomes, 66 outcomes previously unreported by the CTT Collaboration encompassing 555 MedDRA-preferred terms grouped into 15 body systems were prespecified for subsequent analysis (appendix pp 3–6).

### Statistical analysis

All analyses were done according to the intention-to-treat principle. We calculated the log-rank observed minus expected statistic (o – e) and its variance (v) for the first occurrence of each of these 66 outcomes among participants randomly assigned into each trial.^28^ The inverse-variance-weighted average of log of the rate ratio (log RR) across all trials was then calculated as S/V (with variance 1/V, and hence with 95% CI of S/V ± 1·96/√V), where S is the sum of (o − e) over all trials and V is the sum of v over all trials. Analyses were done using SAS version 9.4 and R version 4.5.1.

Because of the large number of outcomes under assessment, we controlled for multiple testing using the Mehrotra and Adewale double false discovery rate (FDR) method.^23,29^ For each outcome, the difference in event rates between treatment groups was deemed to be statistically significant if it was FDR significant at the 5% level (two-sided). Results are reported with nominal (uncorrected) p values and 95% CIs. Consequently, p values below 0·05 and confidence intervals that exclude a relative risk of 1·0 do not necessarily indicate FDR-significant findings. All 66 outcomes were further assessed by analyses of the trials of more intensive statin therapy versus less intensive statin therapy to allow examination of any possible dose-dependent relationships. For any results emerging as FDR significant, effects subdivided by statin intensity, trial, baseline characteristics, and duration of follow-up were also analysed. For completeness, results for muscle and diabetes-related terms (previously reported in detail)^9,10^ are included for low-intensity or moderate-intensity statin therapy and high-intensity statin therapy, but these tests do not contribute to the new set of comparisons tested with FDR control.

Participants in each of the included trials gave informed consent for participation in those trials. Ethics approval for this meta-analysis was granted by the UK National Health Service Health Research Authority (21/SC/0071).

### Role of the funding source

The funders of the meta-analysis project had no role in its design, data collection, data analysis, data interpretation, or writing of the report.

## Results

Individual participant data were available from 19 double-blind trials^30–48^ of any statin regimen versus placebo (123 940 participants, median follow-up 4·5 years [IQR 3·1–5·4]; table). In these trials, mean age of participants was 63 years (SD 9), 89 407 (72%) were men, 34 533 (28%) were women, 59 610 (48%) had previous vascular disease, and 22 925 (18%) had a medical history of diabetes. One trial (6605 participants) compared a low-intensity statin regimen with placebo,^33^ 16 trials (95 890 participants) compared a moderate-intensity statin regimen with placebo,^30–32,34–42,44,45,47,48^ and two trials (21 445 participants) compared a high-intensity statin regimen with placebo.^43,46^ Individual participant data were also available from four double-blind trials of more intensive versus less intensive statin regimens (30 724 participants, median follow-up 5·0 years [IQR 2·3–6·6], mean age 62 years [SD 10]; all with known vascular disease).^49–52^

**Figure 1:**
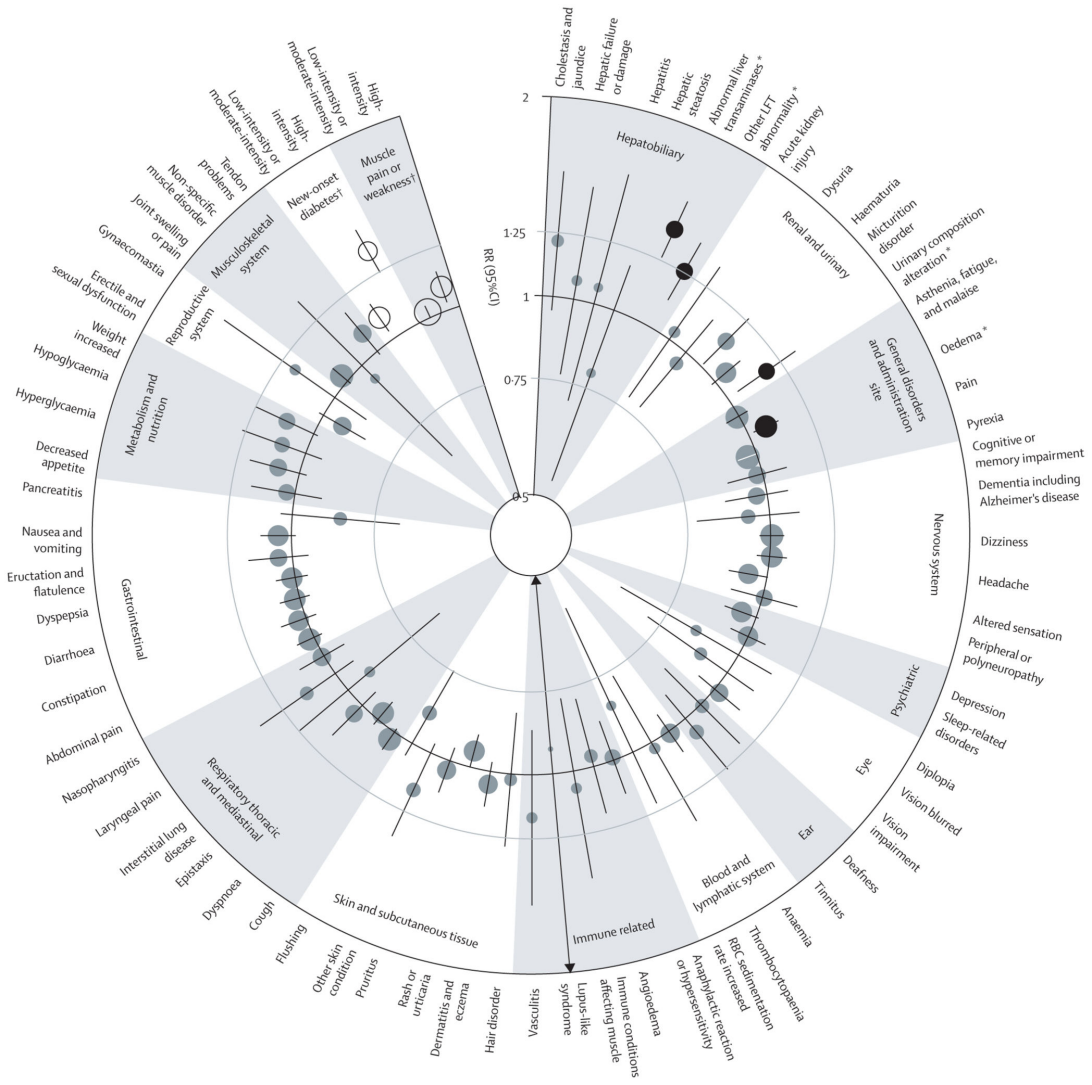
Effect of statin versus placebo on events listed in statin SmPCs, subdivided by component parts Results for two outcomes with fewer than ten events are not shown in the figure, but are included in the appendix. FDR=false discovery rate. LFT=liver function test. RBC=red blood cell. RR=rate ratio. SmPC=Summary of Product Characteristics. *FDR significant at the 5% level; RR for results FDR-significant at the 5% level are indicated by black circles and RR for results not FDR-significant at the 5% level indicated by grey circles. †Excluded from FDR testing; RR for results excluded from FDR testing indicated by white circles.

As compared with placebo, allocation to statin therapy was not associated with any FDR significant excess risk for 62 of the 66 prespecified adverse outcomes of interest across 15 system organ classes (figure 1; appendix pp 7–10). The four outcomes for which an FDR significant excess risk was observed were abnormal liver transaminases (783 of 62 028 participants assigned to statin therapy [0·30% per annum] *vs* 556 of 61 912 participants assigned to placebo [0·22% per annum]; RR 1·41 [95% CI 1·26–1·57]; p<0·0001; absolute annual excess 0·09%); other liver function test abnormalities (which included abnormal alkaline phosphatase, abnormal gamma glutamyl transferase, and non-specific liver-function test abnormalities; appendix p 3; 651 of 62 028 participants assigned to statin therapy [0·25% per annum] *vs* 518 of 61 912 participants assigned to placebo [0·20% per annum]; 1·26 [1·12–1·41]; p=0·00010; absolute annual excess 0·05%); urinary composition alteration (556 of 62 028 participants assigned to statin therapy [0·21% per annum] *vs* 472 of 61 912 participants assigned to placebo [0·18% per annum]; 1·18 [1·04–1·33]; p=0·0089; absolute annual excess 0·03%); and oedema (3495 of 62 028 participants assigned to statin therapy [1·38% per annum] *vs* 3299 of 61 912 participants assigned to placebo [1·31% per annum]; 1·07 [1·02–1·12]; p=0·0071; absolute annual excess 0·07%). Analyses of these four outcomes by statin intensity and trial, baseline characteristics, and duration of follow-up are shown in the appendix (pp 11–22).

**Figure 2:**
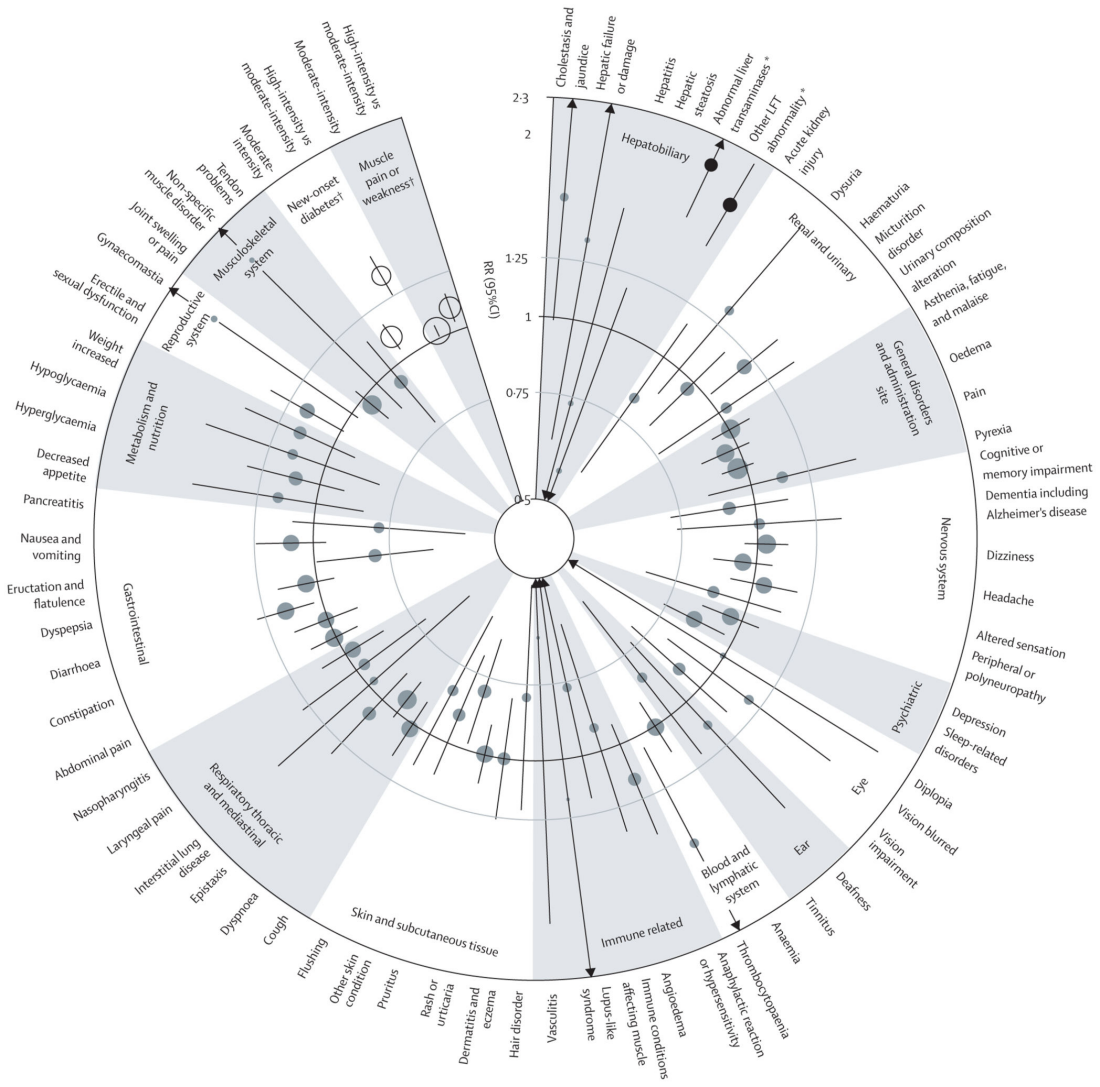
Effect of more intensive statin therapy versus less intensive statin therapy on events listed in statin SmPCs, subdivided by component parts Results for three outcomes with fewer than ten events are not shown in the figure, but are included in the appendix. FDR=false discovery rate. LFT=liver function test. RBC=red blood cell. RR=rate ratio. SmPC=Summary of Product Characteristics. *FDR significant at the 5% level; RR for results FDR significant at the 5% level indicated by black circles and RR for results not FDR-significant at the 5% level indicated by grey circles. †Excluded from FDR testing; RR for results excluded from FDR testing indicated by white circles.

The magnitude of the excess risk for abnormal liver transaminases appeared to be related to statin intensity. Allocation to more intensive statin therapy resulted in an FDR significant excess of abnormal liver transaminases (218 of 15 390 participants assigned to more intensive statin therapy [0·30% per annum] *vs* 102 of 15 334 participants assigned to less intensive statin therapy [0·14% per annum]; 2·06 [1·66–2·57]; p<0·0001; absolute annual excess 0·16%; figure 2; appendix pp 23–24). Consistent with this finding, there was significant heterogeneity between the RRs for low-intensity or moderate-intensity versus placebo and high-intensity versus placebo trials (p>=0·0035; appendix p 11). The results observed for other liver-function test abnormalities were similar: there was an excess risk in trials of more versus less intensive statin therapy (RR 1·87 [1·56–2·24]; p<0·0001; figure 2; appendix pp 23, 25) and there was significant heterogeneity in the RRs for low-intensity or moderate-intensity versus placebo and high-intensity versus placebo trials (p<0·0001; appendix p 12).

The observed findings for an intensity-related increase in risk of liver function test abnormalities were driven by atorvastatin 80 mg per day; in comparisons of high-intensity statin versus placebo, RRs were significantly greater in the trial of atorvastatin 80 mg versus placebo than the trial of rosuvastatin 20 mg versus placebo for both abnormal liver transaminases and other liver function test abnormalities (appendix pp 11, 12). Consequently, after excluding the trial of atorvastatin 80 mg per day versus placebo, the magnitude of the associations observed in the remaining 18 trials was reduced for both abnormal liver transaminases (RR 1·30 [95% CI 1·16–1·46]; p<0·0001) and other liver function test abnormalities (1·09 [0·96–1·23]; p=0·17), and there was no longer significant heterogeneity for these outcomes between the low-intensity or moderate-intensity versus placebo trials and the remaining high-intensity versus placebo trial (p_heterogeneity_=0·56 and p_heterogeneity_=0·39, respectively). Post-hoc analyses combining all terms relating to abnormal liver function test terms into a single outcome did not substantially alter the findings (statin *vs* placebo RR 1·34 [95% CI 1·23–1·45], absolute annual excess 0·13%; appendix pp 26–29); more intensive versus less intensive statin therapy (2·01 [1·74–2·32], absolute annual excess 0·36%; appendix pp 30–33). Post-hoc analyses of statin versus placebo for the specific terms included in the composite term (other liver function test abnormalities) showed similar RRs for the components of increased or abnormal alkaline phosphatase (1·18 [0·86–1·62]) and gamma-glutamyl transferase (1·21 [0·95–1·55]), but a possibly larger RR for non-specific liver function tests or hepatic enzyme increases or abnormalities (1·28 [1·12–1·46]; appendix p 34), with a broadly similar pattern for more intensive versus less intensive statin therapy (appendix p 35). There was no evidence that statins increased the risk of clinical hepatobiliary outcomes, including cholestasis and jaundice, hepatic failure or damage, hepatitis, or hepatic steatosis (figures 1, 2; appendix pp 7, 23).

For the outcome of urinary composition alteration, post-hoc analyses of subcomponents contributing to this outcome showed an excess for the composite of proteinuria, albuminuria, or microalbuminuria (1·20 [1·02–1·42], absolute annual excess 0·02%), but no apparent effect of statin therapy on the presence of white or red blood cells in urine or other urine abnormality (appendix p 36). Analyses of more intensive versus less intensive statin therapy showed no FDR significant excess of urinary composition alteration for those allocated to more intensive statin therapy (figure 2; appendix p 37), and no significant trend or heterogeneity was observed in the trials comparing different intensities of statin therapy versus placebo for this outcome (appendix p 13). There were no FDR significant excesses for any other clinical renal or urinary outcomes, including acute kidney injury, dysuria, haematuria, and micturition disorder (figures 1, 2; appendix pp 8, 38).

For the outcome of oedema, analyses of more intensive versus less intensive statin therapy showed no significant excess (figure 2; appendix p 38), and no significant trend or heterogeneity was observed for the trials comparing different intensities of statin therapy versus placebo for this outcome (appendix p 14). There was no FDR significant excess in any other general disorder conditions (including asthenia, fatigue and malaise, pain, and pyrexia; figures 1, 2; appendix pp 8, 38).

As for the comparison of statin therapy versus placebo, allocation to more intensive versus less intensive statin therapy resulted in no FDR significant excess for any of the other 62 outcomes that were assessed (figure 2; appendix pp 23, 38–40).

## Discussion

Statin therapy has been used by hundreds of millions of people worldwide over the past 30 years, and the data show that statin use has contributed substantively to age-specific reductions in global cardiovascular disease mortality and morbidity. However, concerns about the safety of statins have been raised, with claims of excesses in a wide range of conditions in multiple organ systems. Drug labels for statins include an extensive range of terms listed as potential undesirable effects, but there is a scarcity of compelling evidence to support the inclusion of most of them. Widespread confusion about statin safety hinders the ability of doctors and patients to make informed decisions about initiating or continuing statin therapy. Following the publication in 2012–13 of misleading claims that statins cause side-effects in about a fifth of patients,^53–55^ analyses of prescription data from the UK Clinical Practice Research Datalink found a proportional increase of about 10% in patients stopping statin therapy for secondary and primary cardiovascular prevention, as well as reductions in the numbers of patients who had their cardiovascular risk assessed to assess their eligibility for statin therapy.^20^ The researchers estimated that from October, 2013, to March, 2014, more than 200 000 UK patients stopped taking their statin therapy, which (depending on what proportion resumed treatment) could have resulted in about 2000–6000 avoidable cardiovascular events over the subsequent decade. Similarly, studies in Denmark and Australia found that negative statin-related news stories were followed by increases in the likelihood of patients stopping statin therapy^21^ or reduced numbers of prescriptions of statin therapy being issued, including for patients at particularly high risk of heart attacks and strokes.^22^

By bringing together the individual participant data on all adverse events recorded in large-scale, long-term, double-blind statin trials, we aimed to assess whether statin treatment produced a causal excess of any of these events. This CTT Collaboration project involved the collection of more than 800 datasets and 38 million records, with meticulous attention to categorising all recorded events according to a single nosology. We have already reported significant excess risks of muscle symptoms^9^ and new onset diabetes^10^ (appendix p 41). Our analyses do not support a causal role of statins for the vast majority of events listed as undesirable effects in statin SmPCs, including sleep disturbance, memory loss, sexual dysfunction, depression, and interstitial lung disease, which were previously listed by at least one regulatory agency as a potential statin class effect.^18^ This finding indicates that some of the information provided in statin product labels is unreliable and misleading. Statins were already widely reported to cause liver enzyme abnormalities,^11–13,56–60^ which our analysis confirms. Our analyses identified only two further conditions (urinary composition alteration and oedema) of 66 assessed in which there was a possibility of a causal effect of statin therapy. The terms listed as undesirable effects in statin SmPCs do not necessarily represent the full range of outcomes upon which statins might have an effect, and drug labels other than SmPCs (eg, US Package Inserts) might list more terms than included in our current analyses. Consequently, all other adverse events recorded in these trials are being assessed for subsequent publication.

The observed adverse effect of statin therapy on liver enzyme abnormalities was particularly evident for atorvastatin 80 mg per day (the highest available atorvastatin dose), which resulted in more than a doubling of abnormal liver transaminases, and more than a trebling of other liver function test abnormalities (although with an observed absolute annual excess of <1·6%), which is consistent with previous literature.^61^ The analyses of the more intensive versus less intensive statin comparisons also revealed an FDR significant excess of first reports for abnormal liver transaminases and other liver function test abnormalities in those allocated more intensive statin therapy. These findings suggest a dose-response for liver function tests related to statin intensity. However, there was no evidence of an excess of markers typically indicative of hepatic obstructive pathology (such as alkaline phosphatase or gamma-glutamyl transferase). Importantly, no FDR significant excesses for allocation to statin therapy were observed for any other liver outcomes (including cholestasis and jaundice, hepatic failure or damage, or hepatitis), indicating that there are not typically more serious clinical hepatic sequelae. A previous individual participant data meta-analysis also found no evidence of any adverse effect of statin therapy on liver-related cancer incidence or mortality.^6^ In contrast to reports that statin use might be associated with protection of liver damage in individuals at risk of non-alcoholic steatohepatitis,^62,63^ there was also no clear evidence of a protective effect against hepatic steatosis. The clinical relevance of transaminase elevations with statin use is yet to be fully elucidated, and further work is under way to assess biochemical liver function parameters in more detail. Clarifying the clinical implications of any changes in liver function tests while taking statin therapy would be of value for more informative guidelines regarding monitoring liver transaminases after commencing therapy.^64–67^

The findings for urinary composition alteration and oedema are of uncertain clinical importance, given that the absence of a dose-response in the more intensive versus less intensive statin trials does not support a cause-and-effect relationship. Our post-hoc analyses indicate that although statin therapy might marginally increase urinary protein concentrations, there was no significant impact on any other renal outcomes (including acute kidney injury). This result is in line with findings from the Study of Heart and Renal Protection,^68^ which showed no significant effect of simvastatin plus ezetimibe on the rate of change in estimated glomerular filtration rate compared with placebo, but in contrast to reports from some previous studies of an association of statin therapy with acute renal failure or kidney injury,^11,69^ or an attenuation of progression of kidney function decline and proteinuria.^70–72^ We could not ascertain the severity of oedema in the CTT database, and the observed small increase in proteinuria is unlikely to be responsible for the observed excess. Statin therapy is thought to prevent or delay incident heart failure by reducing the incidence of coronary heart disease and consequent cardiac damage,^73–75^ making it unlikely that the excess of oedema is related to heart failure.

The main strength of this meta-analysis of individual level data obtained from large-scale, double-blind, randomised trials is that it provides reliable estimates of the causal contribution of statins across a wide range of outcomes listed as undesirable effects in SmPCs, which had not previously been systematically assessed. Non-randomised observational studies in which the rates of health outcomes reported in individuals who receive the treatment of interest (and know that they are taking it) and those who do not (and know that they are not) are prone to material biases and confounding, which cannot be guaranteed to be removed through statistical adjustment. Biases can also be introduced by making non-randomised comparisons between rates of events across different trials, not only because the outcome definitions might differ but also because the types of patients studied and the duration of follow-up might differ. Such between-trial comparisons might be seriously misleading, which is the reason why meta-analysis of randomised trials involves statistical methods based on the within-trial differences in a particular outcome.^7,76^ As a consequence, health outcomes do not need to have been obtained in the same way in the different randomised trials contributing to a meta-analysis for comparisons of the rates between the randomly allocated groups within each separate trial to provide unbiased assessments of any real effects of the treatment. We aimed to further ensure the validity of our findings by prespecifying outcomes blind to treatment allocation. The significant excesses identified previously for prespecified muscle^9^ and diabetes-related^10^ outcomes indicates that the absence of excess risks for the numerous other prespecified outcomes in this report is probably robust. In addition, our finding for a lack of effect of statin therapy on cognitive impairment reinforces the findings from the more detailed assessments for this outcome performed in the PROSPER^77^ and HPS^36^ trials.

Our study had some limitations. We cannot exclude the possibility of an excess of adverse effects associated with statin use beyond the durations studied in the included trials. Data were collected in a wide variety of formats from trials whose design and coding methods varied, in many cases years after completion of participating trials, with substantial variation in how stopping of study treatment (such as date, and permanent versus temporary stops) was recorded. Because of this heterogeneity, we were unable to reliably assess whether any outcomes for which there was an observed FDR significant excess led to cessation of study treatment, and whether these outcomes subsequently resolved (which might have added further insights into event severity), or the effect of any treatment rechallenge following a cessation. This limitation might be particularly relevant in relation to liver function tests, given that many trial protocols required monitoring of such parameters and cessation of blinded study treatment (temporary or permanent depending on the circumstances) if they were raised beyond a certain threshold. In addition, all of the outcomes in this report were derived solely from adverse event reports, and hence did not account for analyses of biochemical data. For the vast majority of reported outcomes, this limitation will not have impacted our findings, but such data could further inform analyses of effects on liver function test abnormalities, given that the observed small absolute excesses might be an underestimate. However, substantial liver function dysfunction would typically result in submission of an adverse event report, and the absence of an observed signal for more serious liver outcomes (such as clinical hepatitis) would suggest that the effect of such missing biochemical data is unlikely to materially alter our main findings. That said, any cessation of study treatment because of impaired liver function tests might also have mitigated against more serious clinical liver sequelae.

Despite including randomised data from 154 664 participants enrolled in 23 statin trials with a median follow-up of 4·7 years, the statistical power to detect rare adverse effects (or small relative increases in risk) remains limited, particularly for outcomes with very low event rates and after adjustment for multiple testing. However, for an adverse event with a placebo rate of 1% per year, an RR of 1·09 could have been detected with about 80% power at 2p=0·01 before controlling for multiple testing. Finally, although we sought adverse event data from all of the randomised, double-blind trials included in these analyses, some data were not available (mainly because of data privacy concerns in some of the companies providing the data). However, the missing data corresponded to less than 1% of all participants,^44,46,47^ so they are unlikely to have affected our findings.

In conclusion, this individual participant data meta-analysis of randomised trials has confirmed that statin therapy increases hepatic transaminase and other liver function tests in a statin intensity-related manner, although the absolute excess risks and clinical consequences appear to be low. However, no evidence of causality was found for the vast majority (62 of 66) of other outcomes listed as potential undesirable effects of treatment in statin labels. These findings reinforce previous conclusions that any risks associated with statin therapy are greatly outweighed by their cardiovascular benefits. Consequently, there is a pressing need for regulatory authorities to require revision of statin labels and for other official sources of health information to be updated, so that clinicians, patients, and the public can make informed decisions regarding the balance of the benefits and risks of statin therapy.

## Supplementary Material

Supplementary material

## Figures and Tables

**Table 1:** Participant characteristics

	Year of publication of primary results	Number of included participants*	Treatment regimen (mg/day) ^†^	Follow-up (years)	LDL-C (mmol/L)	Age (years)	Women	Men	White participants^‡^	History of vascular disease	Medical history of diabetes	Timing of scheduled routine follow-up visits	Type of data principally collected
Statin vs placebo													
4S^30^	1994	4444	S20-40 vs placebo	5·4 (5·3–5·6)	4·9 (0·7)	59(7)	827 (19%)	3617 (81%)	NA	4444 (100%)	202 (5%)	Every 1·5 months until 18 months, then every 6–48 months	All AEs
WOSCOPS^31^	1995	6595	P40 vs placebo	4·8 (4·3–5·3)	5·0 (0·5)	55 (6)	0	6595 (100%)	NA	1066 (16%)	77 (1%)	Every 3 months until final follow-up	All AEs
CARE^32^	1996	4159	P40 vs placebo	4·9 (4·4–5·5)	3·6 (0·4)	59(9)	576 (14%)	3583 (86%)	3851 (93%)	4159 (100%)	586 (14%)	At 1·5 months, then every 3 months until 72 months	All AEs
AFCAPS/ TexCAPS^33^	1998	6605	L20-40 vs placebo	50 (4·7–5·8)	3·9 (0·4)	58 (7)	997 (15%)	5608 (85%)	5860 (89%)	0	155 (2%)	Every 1·5 months until 12 months, 15 months, 18 months then every 6 months until 60 months	All AEs
LIPID^34^	1998	9014	P40 vs placebo	5·9 (5·4–6·4)	3·9 (0·8)	61 (8)	1516 (17%)	7498 (83%)	NA	9014 (100%)	782 (9%)	At 3, 6, 9,12 months, then annually until 72 months	SAEs
LIPS^35^	2002	1677	F80 vs placebo	4·0 (3·6–4·0)	3·4 (0·8)	60 (10)	271 (16%)	1406 (84%)	1650 (98%)	1677 (100%)	202 (12%)	At 1·5 and 6 months, then every ~6 months	All AEs
HRS^36^	2002	20536	S40 vs placebo	5·2 (4·6–5·6)	3·4 (0·8)	64 (8)	5082 (25%)	15454 (75%)	19 901 (97%)	17386 (85%)	5963 (29%)	At 4, 8, and 12 months, then every 6 months until 60 months	SAEs + selected AEs
PROSPER^37^	2002	5804	P40 vs placebo	3·3 (3·0–3·5)	3·8 (0·8)	75 (3)	3000 (52%)	2804 (48%)	NA	2565 (44%)	623 (11%)	Every 3 months until final follow-up	All AEs
ASCOT-LLA^33^	2003	10 240	A10 vs placebo	3·3 (2·8–3·7)	3·4 (0·7)	63 (9)	1919 (19%)	8321 (81%)	9687 (95%)	1684 (16%)	2540 (25%)	At 1·5,3, and 6 months, then every 6 months until 66 months or final follow-up	All AEs
ALERT^39^	2003	2102	F40-80 vs placebo	5·5 (5 2–5·6)	4·1 (1·0)	50 (11)	715 (34%)	1387 (66%)	2039 (97%)	409 (19%)	396 (19%)	At 1·5 months then every 6 months until 72 months	All AEs
CARDS^40^	2004	2838	A10 vs placebo	4·2 (3·4–4·9)	2·9 (0·8)	61(8)	909 (32%)	1929 (68%)	2676 (94%)	106 (4%)	2838 (100%)	At 1, 2,3, and 6 months, then every 6 months until 48 months	All AEs
4D^41^	2005	1255	A20 vs placebo	2·7 (1·7–4·0)	3·3 (0·8)	66(8)	578 (46%)	677 (54%)	924 (74%)	1041 (83%)	1255 (100%)	At 1 and 6 months, then every 6 months until 48 months	All AEs
ASPEN^42^	2006	2410	A10 vs placebo	4·0 (2·9–4·5)	2·9 (0·7)	60(8)	811 (34%)	1599 (66%)	2029 (84%)	747 (31%)	2410 (100%)	At 1, 2,3, and 6 months, then every 6 months until 48 months	All AEs
SPARCL^43^	2006	4731	A80 vs placebo	4·9 (44–5·5)	3·5 (0·6)	63 (11)	1908 (40%)	2823 (60%)	4415 (93%)	4731 (100%)	794 (17%)	At 1,3 and 6 months, then every 6 months until 78 months	All AEs
CORONA^44^	2007	4982	R10 vs placebo	2 7 (2·2–3·1)	3·6 (0·9)	72(7)	1175 (24%)	3807 (76%)	NA	4982 (100%)	1473 (30%)	At 1·5 and 3 months, then every 3 months until 51 months or final follow-up	All AEs
GISSI-HF^45^	2008	4574	R10 vs placebo	3·9 (3·0–4·4)	3·1 (0·9)	68 (11)	1032 (23%)	3542 (77%)	4574 (100%)	4574 (100%)	1196 (26%)	At 1,3, and 6 months, then every 6 months until 60 months	SAEs + selected AEs
JUPITER^46^	2008	16714	R20 vs placebo	1·9 (1·5–2·4)	2·7 (0·5)	65 (8)	6374 (38%)	10340 (62%)	NA	0	44 (<1%)	At 3 and 6 months, then every 6 months until 36 months, close out	All AEs
AURORA^47^	2009	2555	R10 vs placebo	3·9 (2 2–4·6)	2·6 (0·9)	64 (9)	969 (38%)	1586 (62%)	NA	1025 (40%)	658 (26%)	At 3 and 6 months, then every 6 months until 42 months	All AEs
HOPE-3^48^	2016	12705	RIO vs placebo	5·5 (51–6·2)	3·3 (0·9)	66 (6)	5874 (46%)	6831 (54%)	2546 (20%)	0	731 (6%)	At 1·5 and 6 months, then every 6 months until 96 months	SAEs + selected AEs
Subtotal (n=19 studies)	··	123 940	··	4·5 (3·1–5·4)	3·5 (0·9)	63 (9)	34533 (28%)	89407 (72%)	60152 (81%)	59 610 (48%)	22 925 (18%)	··	··
More intensive vs less intensive statin (double blind)													
PROVE-IT^49^	2004	4162	A80 vs P40	2·1 (1·9–2·3)	2·6 (0·7)	58 (11)	911 (22%)	3251 (78%)	3776 (91%)	4162 (100%)	762 (18%)	At 0·5,1, and 4 months, then every 4 months until 28 months	All AEs
AtoZ^50^	2004	4497	S40 then S80 vs placebo then S20	2·0 (1·4–2·0)	2·1 (0·5)	60 (11)	1100 (24%)	3397 (76%)	3825 (85%)	4497 (100%)	1059 (24%)	At 1 and 4 months, then every 4 months until 24 months	SAEs + selected AEs
TNT^51^	2005	10001	A80 vs A10	5·0 (4·8–5·3)	2·5 (0·5)	61 (9)	1902 (19%)	8099 (81%)	9410 (94%)	10001 (100%)	1501 (15%)	At 3, 6, 9, and 12 months, then every 6 months until 72 months	All AEs
SEARCH^52^	2010	12 064	S80 vs S20	7·0 (6·5–7·5)	2·5 (0·6)	64 (9)	2052 (17%)	10012 (83%)	11854 (98%)	12 064 (100%)	1267 (11%)	At 2,4, 8, and 12 months, then every 6 months until 84 months	SAEs + selected AEs
Subtotal (n=4 studies)	··	30724	··	5·0 (2·3–6·6)	2·5 (0·6)	62 (10)	5965 (19%)	24759 (81%)	28 865 (94%)	30724(100%)	4589 (15%)	··	··
All trials (n=23 studies)	··	154664	··	4·7 (3·0–5·5)	3·3 (1·0)	63 (9)	40498 (26%)	114166 (74%)	89017 (85%)	90334 (58%)	27514 (18%)	··	··

Data are median (IQR), mean (SD), or n (%) unless otherwise stated. All participants were randomly assigned in a 1:1 allocation. Overall totals and subtotals are calculated across all included trials. AURORA, CORONA, and JUPITER studies supplied age in categorical bands, and so the midpoint of each categorical band was used as a surrogate for baseline age in all analyses. Medical history of diabetes is defined as participants with a history of diabetes and does not include those retrospectively defined as having diabetes at baseline on the basis of adverse events, glucose-lowering medication, or glucose or HbA1c measurements at the time of assignment to a treatment group. Details for collection of adverse events represents the planned data collection timetable. However, not all data were available or could be used (eg, because only provided as summary-level data). 4S=Scandinavian Simvastatin Survival Study. WOSCOPS=West of Scotland Coronary Prevention Study. CARE=Cholesterol And Recurrent Events. AFCAPS/TexCAPS=Air Force–Texas Coronary Atherosclerosis Prevention Study. LIPID=Long-term Intervention with Pravastatin in Ischaemic Disease. LIPS=Lescol Intervention Prevention Study. HPS=Heart Protection Study. PROSPER=PROspective Study of Pravastatin in the Elderly at Risk. ASCOT-LLA=Anglo-Scandinavian Cardiac Outcomes Trial-Lipid Lowering Arm. ALERT=Assessment of Lescol in Renal Transplantation. CARDS=Collaborative Atorvastatin Diabetes Study. 4D=Die Deutsche Diabetes Dialyse Studie. ASPEN=Atorvastatin Study for Prevention of Coronary Heart Disease Endpoints in Non-Insulin-Dependent Diabetes Mellitus. SPARCL=Stroke Prevention by Aggressive Reduction in Cholesterol Levels. CORONA=Controlled Rosuvastatin Multinational Trial in Heart Failure. GISSI-HF=Gruppo Italiano per lo Studio della Sopravvivenza nell’Insufficienza Cardiaca. JUPITER=Justification for the Use of Statins in Prevention: an Intervention Trial Evaluating Rosuvastatin. AURORA=A Study to Evaluate the Use of Rosuvastatin in Subjects on Regular Hemodialysis: an Assessment of Survival and Cardiovascular Events. HOPE-3=Heart Outcomes Prevention Evaluation-3 trial. PROVE-IT=Pravastatin or Atorvastatin Evaluation and Infection Therapy. A to Z=Aggrastat to Zocor. TNT=Treating to New Targets. SEARCH=Study of the Effectiveness of Additional Reductions in Cholesterol and Homocysteine. NA=not available. ··=not applicable.

* A small number of participants in the AURORA (n=218), CORONA (n=27), and JUPITER (n=1088) trials withdrew consent for use of their data post-trial, and hence data from these participants are excluded. The ASCOT-LLA trial excludes 65 participants for whom data were not available because of protocol violations.

† S indicates simvastatin, P indicates pravastatin, F indicates fluvastatin, A indicates atorvastatin, R indicates rosuvastatin, and L indicates lovastatin; numbers following abbreviation indicate statin daily dose in mg.

‡ Percentages were calculated after excluding the seven trials where information on race and ethnicity was not provided (the relevant denominators are therefore 73832 for all trials of statin *vs* placebo and 104 556 for all trials).

## Data Availability

Individual participant data from each contributing trial have been provided to the CTT Collaboration on the understanding that they would be used only for the purpose of the CTT meta-analyses and would not be released to others. Requests for such data should be made directly to the data custodians of each trial. The CTT data policy can be found on the project website (https://www.cttcollaboration.org/).

## Research in context

### Evidence before this study

We searched Ovid MEDLINE, Ovid Embase, and the Cochrane Database of Systematic Reviews from their date of inception to Dec 15, 2025 for meta-analyses and review articles, published in any language, which specifically assessed the effects of statin regimens on all outcomes listed as undesirable effects in statin product labels (Summaries of Product Characteristics [SmPC]). We searched using a combination of appropriate MeSH terms for meta-analyses and review articles (eg, “meta-analysis” or “systematic review” or “scoping review” or “network meta-analysis”), statins (eg, “statins” or “Hydroxymethylglutaryl-CoA Reductase Inhibitors”), and terms related to product information documents (eg, “Summary of Product Characteristics” or “SmPC” or “Patient Information Leaflet” or “Package insert” or “pil” or “Product Information” or “USPI” or “United States prescribing information” or “product insert”). Although some articles described the effect of statin therapy on a range of potential undesirable effects, no comprehensive assessment of all such terms appears to have been undertaken previously. Data from randomised controlled trials have shown that statin therapy can, rarely, cause substantial muscle damage or, in a more severe form, rhabdomyolysis as indicated by muscle symptoms accompanied by related biochemical changes (eg, multifold rises in creatine kinase). Recent individual participant data meta-analyses have also shown that statin therapy causes a small relative increase in less severe muscle symptoms largely confined to the first year of treatment. Individual participant data meta-analyses have also shown a moderate dose-dependent increase in new diagnoses of diabetes, the majority occurring in individuals with glycaemic markers already close to the diagnostic threshold for diabetes at the time of initiation of statin treatment. Statin SmPCs also list numerous other adverse (ie, undesirable) outcomes as possible effects of statin therapy. However, these attributions typically derive from case reports or observational studies, which can be subject to bias and confounding. Therefore, robust evidence is needed to clarify the possible effects of statin therapy on these outcomes to support informed decision-making by patients and clinicians.

### Added value of this study

We aimed to minimise the risk of biases by restricting our analyses to large-scale, randomised, double-blind trials of statin therapy in which there was systematic and unbiased event reporting. We obtained details of all adverse events recorded in each individual trial participant, and coded them using standard nosology (from the Medical Dictionary for Regulatory Activities). The availability of individual participant data permitted assessment of any causal effects of statin therapy on health outcomes currently listed as possible undesirable effects in statin labelling. A false discovery rate (FDR) multiple-testing method was used to control for the number of health outcomes investigated. Results from randomised placebo-controlled double-blind trials showed that, after controlling for multiple testing using the FDR method, statin therapy was associated with a significant excess risk for only four of 66 prespecified outcomes: abnormal liver transaminases, other liver function test abnormalities, urinary composition alteration, and oedema. The absolute annual excesses for each of these outcomes was very small (<0·1%). The effect on liver function tests appeared to be related to statin intensity because a similar excess was also observed in trials comparing more intensive with less intensive statin therapy; however, this association was not seen for alteration in urinary composition nor for oedema. No significant excess risk was observed for any other hepatobiliary outcomes, nor for any of the other 62 prespecified outcomes including cognitive impairment, depression, sleep disturbance, erectile and sexual dysfunction, peripheral neuropathy, acute kidney injury, and interstitial lung disease.

### Implications of all the available evidence

These findings indicate that, in addition to the previously reported adverse effects of statin therapy on muscle outcomes and diabetes, statins are associated only with small absolute increases in abnormal liver biochemistry, and possible adverse effects of unknown clinical relevance on urinary composition and oedema, but not with any other outcomes listed in statin SmPCs. Consequently, the undesirable effect sections of statin product labels might overstate risks and mislead clinicians and patients, and should be revised to better support informed, evidence-based decision making.
