# Supplementary material for: Assessment of adverse effects attributed to statin therapy in product labels: a meta-analysis of double-blind randomised controlled trials
Source: Lancet. Author manuscript; Available in PMC 2026 Apr 15. (PMC7619005; doi:10.1016/S0140-6736(25)01578-8)
Supplement: Supplementary material [file EMS212506-supplement-Supplementary_material.pdf]

THE LANCET

Supplementary appendix

This appendix formed part of the original submission and has been peer reviewed. We post it as supplied by the authors.

Supplement to: Cholesterol Treatment Trialists' (CTT) Collaboration. Assessment of adverse effects attributed to statin therapy in product labels: a meta-analysis of double-blind randomised controlled trials. *Lancet* 2026; published online Feb 5. https://doi.org/10.1016/S0140-6736(25)01578-8.

Assessment of adverse effects attributed to statin therapy in product labels: a meta-analysis of double-blind randomised controlled trials

Web appendix: Table of Contents

			Page
**Webtables**			
**1**	Statin SmPC outcomes assessed in CTT analyses		**3**
**Webfigures**			
**1**	**<u>Statin vs placebo</u>; results overall subdivided by category component parts:**		
	**a**	Gastrointestinal, hepatobiliary, and metabolism and nutrition disorders	**7**
	**b**	Renal & urinary, reproductive system & breast, musculoskeletal & connective tissue disorders and general disorders & administration site conditions	**8**
	**c**	Nervous system, psychiatric, eye, ear & labyrinth disorders	**9**
	**d**	Blood & lymphatic system, immune related, skin & subcutaneous tissue, and respiratory, thoracic & mediastinal disorders	**10**
**2**	**<u>Statin vs placebo</u>; FDR significant outcomes subdivided by statin intensity and trial:**		
	**a**	Hepatobiliary disorders: abnormal liver transaminases	**11**
	**b**	Hepatobiliary disorders: other liver function test abnormality	**12**
	**c**	Renal & urinary disorders: urinary composition alteration	**13**
	**d**	General disorders & administration site conditions: oedema	**14**
**3**	**<u>Statin vs placebo</u>; FDR significant outcomes subdivided by baseline characteristics:**		
	**a**	Hepatobiliary disorders: abnormal liver transaminases	**15**
	**b**	Hepatobiliary disorders: other liver function test abnormality	**16**
	**c**	Renal & urinary disorders: urinary composition alteration	**17**
	**d**	General disorders & administration site conditions: oedema	**18**
**4**	**<u>Statin vs placebo</u>; FDR significant outcomes by duration of follow-up:**		
	**a**	Hepatobiliary disorders: abnormal liver transaminases	**19**
	**b**	Hepatobiliary disorders: other liver function test abnormality	**20**
	**c**	Renal & urinary disorders: urinary composition alteration	**21**
	**d**	General disorders & administration site conditions: oedema	**22**
**5**	**<u>More vs less intensive statin therapy</u>; Hepatobiliary disorders:**		
	**a**	Subdivided by category component parts	**23**
	**b**	Abnormal liver transaminases, subdivided by statin intensity and trial	**24**
	**c**	Other liver function test abnormality, subdivided by statin intensity and trial	**25**
**6**	**<u>Post-hoc analysis statin vs placebo</u>; Hepatobiliary disorders: Abnormal liver function tests subdivided by:**		
	**a**	Category component parts	**26**
	**b**	Statin intensity and trial	**27**
	**c**	Baseline characteristics	**28**
	**d**	Duration of follow-up	**29**
**7**	**<u>Post-hoc analysis more vs less intensive statin therapy</u>; Hepatobiliary disorders: Abnormal liver function tests subdivided by:**		
	**a**	Category component parts	**30**
	**b**	Statin intensity and trial	**31**
	**c**	Baseline characteristics	**32**
	**d**	Duration of follow-up	**33**
**8**	**<u>Post-hoc analysis</u>; Hepatobiliary disorders: other liver function test abnormality, subdivided by component parts:**		
	**a**	**Statin vs placebo**	**34**
	**b**	**More vs less intensive statin therapy**	**35**
**9**	**<u>Post-hoc analysis</u>; Renal & urinary disorders: urinary composition alteration, subdivided by component parts:**		
	**a**	**Statin vs placebo**	**36**
	**b**	**More vs less intensive statin therapy**	**37**
**10**	**<u>More vs less intensive statin therapy</u>; subdivided by category component parts:**		
	**a**	Renal & urinary, reproductive system & breast, musculoskeletal & connective tissue disorders and general disorders & administration site conditions	**38**
	**b**	Nervous system, psychiatric, eye, ear & labyrinth disorders	**39**
	**c**	Blood & lymphatic system, immune related, skin & subcutaneous tissue, and respiratory, thoracic & mediastinal disorders	**40**
**11**	**<u>Statin vs placebo</u>; Any muscle pain or weakness and new-onset diabetes by statin intensity**		**41**

Webtable 1: Statin SmPC outcomes assessed in CTT analyses

System Organ Class	CTT analysis outcome description	Direct/close match included MedDRA Preferred Terms
**Blood and lymphatic system disorders**		
	Anaemia	Anaemia; Anaemia macrocytic; Anaemia megaloblastic; Hyperchromic anaemia; Hypochromic anaemia; Microcytic anaemia; Normochromic normocytic anaemia
	Eosinophilia	Eosinophilia; Hypereosinophilic syndrome; Eosinophilia myalgia syndrome
	Thrombocytopenia	Thrombocytopenia; Platelet production decreased; Plateletcrit decreased; Platelet count decreased
	Red blood cell sedimentation rate increased	Red blood cell sedimentation rate increased; Red blood cell sedimentation rate abnormal
**Ear and labyrinth disorders**		
	Deafness	Deafness; Deafness bilateral; Deafness neurosensory; Deafness permanent; Deafness transitory; Deafness unilateral; Mixed deafness
	Tinnitus	Tinnitus
**Eye disorders**		
	Diplopia	Diplopia; Heteronymous diplopia; Homonymous diplopia
	Vision blurred	Vision blurred
	Visual impairment	Visual impairment; Visual acuity reduced; Visual acuity tests abnormal; Visual field tests abnormal
**Gastrointestinal disorders**		
	Abdominal pain	Abdominal discomfort; Abdominal pain; Abdominal pain lower; Abdominal pain upper
	Constipation	Constipation; Infrequent bowel movements
	Diarrhoea	Diarrhoea; Frequent bowel movements
	Dyspepsia	Dyspepsia; Biliary dyspepsia; Epigastric discomfort
	Eructation and flatulence	Eructation; Flatulence
	Nausea and vomiting	Nausea; Vomiting
	Pancreatitis	Pancreatitis; Haemorrhagic necrotic pancreatitis; Oedematous pancreatitis; Pancreatitis acute; Pancreatitis chronic; Pancreatitis haemorrhagic; Pancreatitis necrotising; Pancreatitis relapsing; Pancreatic failure; Pancreatic necrosis; Pancreatic toxicity
**Hepatobiliary disorders**		
	Cholestasis and jaundice	Cholestasis; Jaundice; Jaundice hepatocellular; Bilirubin conjugated abnormal; Bilirubin conjugated increased; Blood bilirubin increased; Jaundice cholestatic; Jaundice extrahepatic obstructive; Jaundice acholuric
	Hepatic failure or damage	Hepatic failure; Acute hepatic failure; Acute on chronic liver failure; Chronic hepatic failure; Subacute hepatic failure; Hepatic necrosis; Liver disorder; Drug-induced liver injury; Hepatocellular injury; Hepatotoxicity; Liver injury; Mixed liver injury; Coma hepatic; Hepatic encephalopathy
	Hepatitis	Hepatitis; Hepatitis acute; Hepatitis chronic active; Hepatitis chronic persistent; Hepatitis fulminant; Hepatitis toxic; Chronic hepatitis
	Hepatic steatosis	Hepatic steatosis; Non-alcoholic steatohepatitis; Steatohepatitis; Non-alcoholic fatty liver
	Abnormal liver transaminases	Alanine aminotransferase abnormal; Alanine aminotransferase increased; Aspartate aminotransferase abnormal; Aspartate aminotransferase increased; Transaminases abnormal; Transaminases increased
	Other liver function test abnormality	Blood alkaline phosphatase increased; Blood alkaline phosphatase abnormal; Gamma-glutamyltransferase increased; Gamma-glutamyltransferase abnormal; Liver function test abnormal; Liver function test increased; Hepatic function abnormal; Hepatic enzyme abnormal; Hepatic enzyme increased

System Organ Class	CTT analysis outcome description	Direct/close match included MedDRA Preferred Terms
**Immune related disorders**		
	Anaphylactic reaction or hypersensitivity	Anaphylactic reaction; Anaphylactic shock; Anaphylactoid reaction; Anaphylactoid shock; Anaphylaxis treatment; Hypersensitivity; Drug hypersensitivity; Type I hypersensitivity; Type II hypersensitivity; Type III immune complex mediated reaction; Type IV hypersensitivity reaction
	Angioedema	Angioedema; Allergic oedema; Epiglottic oedema; Gingival oedema; Idiopathic angioedema; Laryngeal oedema; Laryngotracheal oedema; Nasal oedema; Oedema mouth; Oedema mucosal; Palatal oedema; Pharyngeal oedema; Respiratory tract oedema; Tongue oedema; Tracheal oedema; Gingival swelling; Mouth swelling; Oropharyngeal swelling; Palatal swelling; Oculorespiratory syndrome; Swollen tongue; Face oedema; Circumoral oedema; Eye oedema; Eyelid oedema; Lip oedema; Orbital oedema; Periorbital oedema; Eye swelling; Lip swelling; Swelling face
	Immune conditions affecting muscle	Immune mediated necrotising myopathy; Polymyalgia rheumatica; Polymyositis; Dermatomyositis; Myasthenia gravis; Myasthenia gravis crisis; Ocular myasthenia
	Lupus-like syndrome	Lupus-like syndrome; Acute cutaneous lupus erythematosus; Central nervous system lupus; Chronic cutaneous lupus erythematosus; Cutaneous lupus erythematosus; Lupus cystitis; Lupus encephalitis; Lupus endocarditis; Lupus enteritis; Lupus hepatitis; Lupus myocarditis; Lupus nephritis; Lupus pancreatitis; Lupus pleurisy; Lupus pneumonitis; Lupus vasculitis; Neuropsychiatric lupus; Pericarditis lupus; Peritonitis lupus; Subacute cutaneous lupus erythematosus; Systemic lupus erythematosus; Systemic lupus erythematosus disease activity index abnormal; Systemic lupus erythematosus disease activity index increased; Systemic lupus erythematosus rash
	Serious skin drug reactions	Drug reaction with eosinophilia and systemic symptoms; Stevens-Johnson syndrome; Toxic epidermal necrolysis
	Vasculitis	Vasculitis; Cutaneous vasculitis; Diffuse vasculitis; Haemorrhagic vasculitis; Hypersensitivity vasculitis; Nodular vasculitis; Ocular vasculitis; Pulmonary vasculitis; Renal vasculitis; Retinal vasculitis; Urticarial vasculitis; Vasculitis cerebral; Vasculitis gastrointestinal; Vasculitis necrotising; Henoch-Schonlein purpura
**Metabolism and nutrition disorders**		
	Decreased appetite	Decreased appetite
	Hyperglycaemia	Hyperglycaemia; Blood glucose increased; Glycosylated haemoglobin increased; Hyperglycaemic seizure; Hyperglycaemic unconsciousness
	Hypoglycaemia	Hypoglycaemia; Hypoglycaemic coma; Hypoglycaemic encephalopathy; Hypoglycaemic seizure; Hypoglycaemic unconsciousness; Shock hypoglycaemic
	Weight increased	Weight increased; Body mass index increased; Abnormal weight gain
**Musculoskeletal and connective tissue disorders**		
	Joint swelling or pain	Arthralgia; Arthritis; Polyarthritis; Joint swelling
	Non-specific muscle disorder	Muscle disorder; Extraocular muscle disorder; Muscle tone disorder; Papillary muscle disorder; Muscle rupture
	Tendon problems	Tendon disorder; Tendonitis; Tendon rupture; Enthesopathy; Epicondylitis; Tendinous contracture; Tendon calcification; Tendon discomfort; Tendon laxity; Tendon necrosis; Tendon pain; Tendon sheath disorder; Tenosynovitis; Trigger finger
**Nervous system disorders**		
	Cognitive or memory impairment	Amnesia; Amnestic disorder; Memory impairment; Borderline mental impairment; Bradyphrenia; Cognitive disorder; Cognitive linguistic deficit; Disturbance in attention; Judgement impaired; Mental fatigue; Mental impairment; Confusional state; Disorientation; Vascular cognitive impairment
	Dementia including Alzheimer’s disease	Corticobasal degeneration; Dementia; Dementia Alzheimer's type; Dementia of the Alzheimer's type, uncomplicated; Dementia of the Alzheimer's type, with

System Organ Class	CTT analysis outcome description	Direct/close match included MedDRA Preferred Terms
		delirium; Dementia of the Alzheimer's type, with delusions; Dementia of the Alzheimer's type, with depressed mood; Dementia with Lewy bodies; Frontotemporal dementia; Mixed dementia; Posterior cortical atrophy; Presenile dementia; Prodromal Alzheimer's disease; Progressive supranuclear palsy; Senile dementia; Vascular dementia; Vascular encephalopathy
	Dizziness	Dizziness; Presyncope; Syncope
	Headache	Headache; Cluster headache; New daily persistent headache; Typical aura without headache; Basilar migraine; Hemiplegic migraine; Migraine; Migraine with aura; Migraine without aura; Ophthalmoplegic migraine; Retinal migraine; Status migrainosus; Vestibular migraine
	Altered sensation	Dysaesthesia; Dysaesthesia pharynx; Oral dysaesthesia; Thermohyperaesthesia; Dysgeusia; Hypoaesthesia; Anal hypoaesthesia; Genital hypoaesthesia; Hemianaesthesia; Hypoaesthesia eye; Hypoaesthesia oral; Pharyngeal hypoaesthesia; Thermoanaesthesia; Thermohypoaesthesia; Vulvovaginal hypoaesthesia; Paraesthesia; Anal paraesthesia; Eye paraesthesia; Genital paraesthesia; Hemiparaesthesia; Intranasal paraesthesia; Meralgia paraesthetica; Notalgia paraesthetica; Paraesthesia ear; Paraesthesia mucosal; Paraesthesia oral; Pharyngeal paraesthesia; Sensory disturbance; Anorectal sensory loss; Eyelid sensory disorder; Sensory level abnormal; Sensory loss
	Peripheral or poly-neuropathy	Neuropathy peripheral; Acute motor axonal neuropathy; Acute motor-sensory axonal neuropathy; Acute polyneuropathy; Anti-myelin-associated glycoprotein associated polyneuropathy; Autoimmune neuropathy; Autonomic neuropathy; Axonal neuropathy; Cardiac autonomic neuropathy; Chronic inflammatory demyelinating polyradiculoneuropathy; Demyelinating polyneuropathy; Diabetic amyotrophy; Diabetic autonomic neuropathy; Diabetic mononeuropathy; Diabetic neuropathy; Multifocal motor neuropathy; Neuronal neuropathy; Peripheral motor neuropathy; Peripheral sensorimotor neuropathy; Peripheral sensory neuropathy; Polyneuropathy; Polyneuropathy chronic; Polyneuropathy idiopathic progressive; Small fibre neuropathy; Toxic neuropathy; Toxic optic neuropathy; Guillain-Barre syndrome; Miller Fisher syndrome; Lewis-Sumner syndrome
**Psychiatric disorders**		
	Depression	Depression; Adjustment disorder with depressed mood; Adjustment disorder with mixed anxiety and depressed mood; Agitated depression; Antidepressant therapy; Depression suicidal; Major depression; Persistent depressive disorder; Depressed mood; Depressive symptom; Feeling of despair; Feelings of worthlessness; Morose; Negative thoughts; Depressive delusion
	Sleep-related disorders	Insomnia; Hyposomnia; Initial insomnia; Middle insomnia; Terminal insomnia; Nightmare; Sleep terror; Sleep disorder
**Renal and urinary disorders**		
	Acute kidney injury	Acute kidney injury
	Dysuria	Dysuria
	Haematuria	Haematuria
	Micturition disorder	Micturition disorder; Micturition frequency decreased; Micturition urgency; Nocturia; Pollakiuria; Urinary hesitation; Urinary incontinence; Urinary retention; Urinary straining; Urine flow decreased; Terminal dribbling; Enuresis
	Urinary composition alteration	Proteinuria; Albuminuria; Microalbuminuria; White blood cells urine positive; Red blood cells urine; Red blood cells urine positive; Urine abnormality
**Reproductive system and breast disorders**		
	Erectile and sexual dysfunction	Erectile dysfunction; Sexual dysfunction; Female sexual dysfunction; Male sexual dysfunction; Libido decreased; Loss of libido
	Gynaecomastia	Gynaecomastia
**Respiratory, thoracic and mediastinal disorders**		
	Cough	Cough

System Organ Class	CTT analysis outcome description	Direct/close match included MedDRA Preferred Terms
	Dyspnoea	Dyspnoea; Dyspnoea at rest; Dyspnoea exertional
	Epistaxis	Epistaxis
	Interstitial lung disease	Interstitial lung disease; Pulmonary fibrosis
	Laryngeal pain	Laryngeal pain; Laryngeal discomfort
	Nasopharyngitis	Nasopharyngitis
**Skin and subcutaneous tissue disorders**		
	Hair disorder	Alopecia; Alopecia areata; Alopecia scarring; Alopecia totalis; Alopecia universalis; Diffuse alopecia; Hair colour changes; Hair disorder; Hair growth abnormal; Hair growth rate abnormal
	Dermatitis and eczema	Dermatitis; Dermatitis bullous; Eczema; Eczema eyelids; Eczema weeping; Hand dermatitis
	Rash or urticaria	Rash; Skin reaction; Photosensitivity reaction; Exfoliative rash; Eyelid rash; Genital rash; Mucocutaneous rash; Nodular rash; Perineal rash; Rash erythematous; Rash follicular; Rash generalised; Rash macular; Rash maculo-papular; Rash maculovesicular; Rash morbilliform; Rash papular; Rash papulosquamous; Rash pruritic; Rash pustular; Rash vesicular; Vulvovaginal rash; Urticaria; Chronic spontaneous urticaria; Haemorrhagic urticaria; Idiopathic urticaria; Urticaria chronic; Urticaria papular; Urticaria vesiculosa
	Pruritus	Pruritus; Anal pruritus; Ear pruritus; Eye pruritus; Eyelids pruritus; Gingival pruritus; Lip pruritus; Nasal pruritus; Oral pruritus; Pruritus generalised; Pruritus genital; Tongue pruritus; Vulvovaginal pruritus
	Other skin condition	Erythema multiforme; Lichenoid keratosis; Skin disorder
	Flushing	Flushing; Hot flush
**General disorders and administration site conditions**		
	Asthenia, Fatigue, and malaise	Asthenia; Chronic fatigue syndrome; Decreased activity; Fatigue; Lethargy; Listless; Malaise; Sluggishness
	Oedema	Oedema; Generalised oedema; Oedema peripheral; Skin oedema
	Pain	Pain; Adnexa uteri pain; Axillary pain; Bladder pain; Bone pain; Breakthrough pain; Breast pain; Central pain syndrome; Chest pain; Chronic idiopathic pain syndrome; Complex regional pain syndrome; Ear pain; External ear pain; Eye pain; Eyelid pain; Facial pain; Gallbladder pain; Gastrointestinal pain; Genital pain; Genito-pelvic pain or penetration disorder; Gingival pain; Hepatic pain; Hernia pain; Ligament pain; Lip pain; Lymph node pain; Masticatory pain; Mucosal pain; Myofascial pain syndrome; Neuromuscular pain; Nipple pain; Non-cardiac chest pain; Oesophageal pain; Oral pain; Oropharyngeal pain; Pain in jaw; Pain of skin; Pain threshold decreased; Painful ejaculation; Painful erection; Painful os peroneum syndrome; Painful respiration; Paradoxical pain; Patellofemoral pain syndrome; Pelvic pain; Penile pain; Perineal pain; Phantom pain; Pleuritic pain; Prostatic pain; Pubic pain; Pulmonary pain; Radicular pain; Renal pain; Salivary gland pain; Scar pain; Scrotal pain; Sinus pain; Spermatic cord pain; Spinal pain; Suprapubic pain; Testicular pain; Thyroid pain; Tracheal pain; Urethral pain; Urinary tract pain; Uterine cervical pain; Uterine pain; Vascular pain; Visceral pain; Vulvovaginal pain
	Pyrexia	Pyrexia; Hyperpyrexia; Body temperature increased

Webfigure 1a: Effect of statin vs placebo on GASTROINTESTINAL, HEPATOBILIARY, AND METABOLISM AND NUTRITION DISORDERS listed in statin SmPCs, subdivided by category component parts

Endpoint	Events (% per annum) Statin (n=62028)	Events (% per annum) Placebo (n=61912)	Observed−Expected O−E	Observed−Expected Var(O−E)	Rate Ratio (95% CI)
**Gastrointestinal disorders**					
Abdominal pain	3443 (1.4)	3367 (1.3)	35.4	1702.1	1.02 (0.97 − 1.07)
Constipation	1865 (0.7)	1810 (0.7)	28.3	918.6	1.03 (0.97 − 1.10)
Diarrhoea	2707 (1.1)	2649 (1.0)	28.3	1338.6	1.02 (0.97 − 1.08)
Dyspepsia	2472 (1.0)	2430 (1.0)	18.5	1225.2	1.02 (0.96 − 1.07)
Eructation and flatulence	722 (0.3)	683 (0.3)	18.3	351.2	1.05 (0.95 − 1.17)
Nausea and vomiting	2111 (0.8)	1999 (0.8)	51.3	1027.2	1.05 (0.99 − 1.12)
Pancreatitis	164 (<0.1)	192 (<0.1)	−14.5	89.0	0.85 (0.69 − 1.05)
**Hepatobiliary disorders**					
Cholestasis and jaundice	144 (<0.1)	118 (<0.1)	12.8	65.5	1.22 (0.95 − 1.55)
Hepatic failure or damage	73 (<0.1)	68 (<0.1)	2.4	35.2	1.07 (0.77 − 1.49)
Hepatitis	48 (<0.1)	45 (<0.1)	1.4	23.2	1.06 (0.71 − 1.59)
Hepatic steatosis	43 (<0.1)	54 (<0.1)	−5.6	24.2	0.79 (0.53 − 1.18)
Abnormal liver transaminases *	783 (0.3)	556 (0.2)	114.2	334.7	1.41 (1.26 − 1.57)
Other LFT abnormality *†	651 (0.3)	518 (0.2)	66.6	292.2	1.26 (1.12 − 1.41)
**Metabolism and nutrition disorders ****					
Decreased appetite	503 (0.2)	486 (0.2)	8.4	247.2	1.03 (0.91 − 1.17)
Hyperglycaemia	766 (0.3)	706 (0.3)	29.7	367.9	1.08 (0.98 − 1.20)
Hypoglycaemia	373 (0.1)	338 (0.1)	16.3	177.7	1.10 (0.95 − 1.27)
Weight increased	578 (0.2)	516 (0.2)	29.5	273.5	1.11 (0.99 − 1.25)

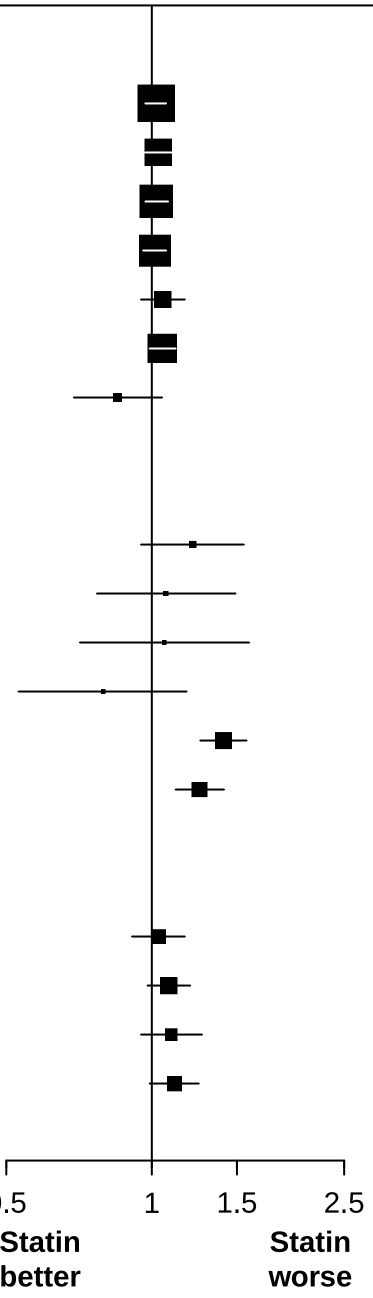

* FDR significant at the 5% level

† LFT=Liver function test

** The outcome diabetes mellitus is also included in statin labels as an potential undesirable effect but has previously been reported separately: <DOI: https://doi.org/10.1016/S2213−8587(24)00040−8>

Webfigure 1b: Effect of statin vs placebo on RENAL AND URINARY, REPRODUCTIVE SYSTEM AND BREAST, MUSCULOSKELETAL AND CONNECTIVE TISSUE DISORDERS, AND GENERAL DISORDERS AND ADMINISTRATION SITE CONDITIONS listed in statin SmPCs, subdivided by category component parts

Endpoint	Events (% per annum)		Observed−Expected		Rate Ratio (95% CI)
	Statin (n=62028)	Placebo (n=61912)	O−E	Var(O−E)	
**Renal and urinary disorders**					
Acute kidney injury	103 (<0.1)	99 (<0.1)	1.6	50.5	1.03 (0.78 − 1.36)
Dysuria	192 (<0.1)	201 (<0.1)	−5.0	98.2	0.95 (0.78 − 1.16)
Haematuria	656 (0.3)	578 (0.2)	37.8	308.5	1.13 (1.01 − 1.26)
Micturition disorder	1906 (0.7)	1814 (0.7)	45.0	929.8	1.05 (0.98 − 1.12)
Urinary composition alteration *	556 (0.2)	472 (0.2)	41.9	256.9	1.18 (1.04 − 1.33)
**Reproductive system and breast disorders**					
Erectile and sexual dysfunction	869 (0.3)	926 (0.4)	−31.9	448.7	0.93 (0.85 − 1.02)
Gynaecomastia	92 (<0.1)	77 (<0.1)	7.3	42.2	1.19 (0.88 − 1.61)
**Musculoskeletal and connective tissue disorders**					
Joint swelling or pain	4774 (1.9)	4626 (1.9)	73.5	2349.2	1.03 (0.99 − 1.07)
Non−specific muscle disorder	52 (<0.1)	55 (<0.1)	−1.6	26.7	0.94 (0.64 − 1.37)
Tendon problems	899 (0.3)	822 (0.3)	36.2	430.2	1.09 (0.99 − 1.20)
**General disorders and administration site conditions**					
Asthenia, fatigue and malaise	4253 (1.7)	4281 (1.7)	−19.5	2132.5	0.99 (0.95 − 1.03)
Oedema *	3495 (1.4)	3299 (1.3)	110.8	1695.9	1.07 (1.02 − 1.12)
Pain	5995 (2.4)	6175 (2.5)	−103.4	3041.4	0.97 (0.93 − 1.00)
Pyrexia	675 (0.3)	687 (0.3)	−7.4	340.5	0.98 (0.88 − 1.09)

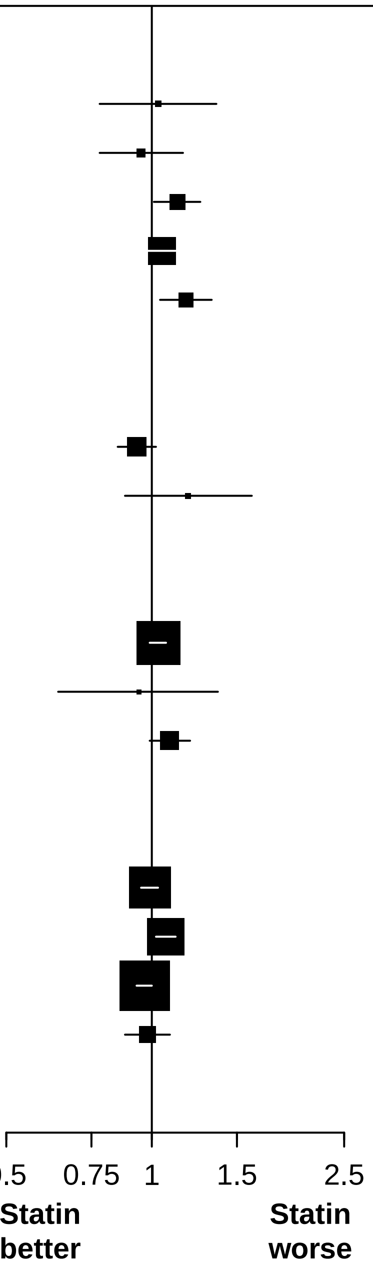

* FDR significant at the 5% level.

Webfigure 1c: Effect of statin vs placebo on NERVOUS SYSTEM, PSYCHIATRIC, EYE, AND EAR AND LABYRINTH DISORDERS listed in statin SmPCs, subdivided by category component parts

Endpoint	Events (% per annum) Statin (n=62028)	Events (% per annum) Placebo (n=61912)	Observed−Expected O−E	Observed−Expected Var(O−E)	Rate Ratio (95% CI)
**Nervous system disorders**					
Cognitive or memory impairment	616 (0.2)	639 (0.2)	−12.3	313.7	0.96 (0.86 − 1.07)
Dementia including Alzheimer's	231 (<0.1)	249 (<0.1)	−9.3	120.0	0.93 (0.77 − 1.11)
Dizziness	4156 (1.6)	4140 (1.7)	3.3	2073.5	1.00 (0.96 − 1.05)
Headache	2808 (1.1)	2790 (1.1)	6.7	1398.9	1.00 (0.95 − 1.06)
Altered sensation	1573 (0.6)	1673 (0.7)	−55.5	811.4	0.93 (0.87 − 1.00)
Peripheral or poly−neuropathy	543 (0.2)	537 (0.2)	0.9	269.9	1.00 (0.89 − 1.13)
**Psychiatric disorders**					
Depression	1910 (0.7)	2016 (0.8)	−55.5	981.3	0.95 (0.89 − 1.01)
Sleep−related disorders	1728 (0.7)	1731 (0.7)	−3.1	864.6	1.00 (0.93 − 1.07)
**Eye disorders**					
Diplopia	77 (<0.1)	91 (<0.1)	−7.3	42.0	0.84 (0.62 − 1.14)
Vision blurred	144 (<0.1)	161 (<0.1)	−8.9	76.2	0.89 (0.71 − 1.11)
Vision impairment	895 (0.3)	881 (0.3)	7.8	443.9	1.02 (0.93 − 1.12)
**Ear and labyrinth disorders**					
Deafness	228 (<0.1)	225 (<0.1)	0.5	113.2	1.00 (0.84 − 1.21)
Tinnitus	279 (0.1)	262 (0.1)	7.9	135.2	1.06 (0.90 − 1.25)

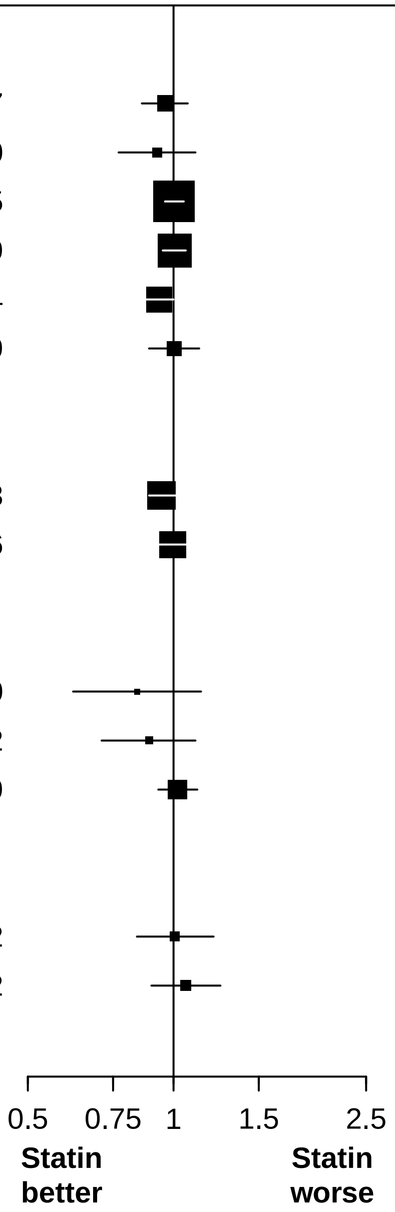

Webfigure 1d: Effect of statin vs placebo on BLOOD AND LYMPHATIC SYSTEM, IMMUNE RELATED, SKIN AND SUBCUTANEOUS TISSUE, AND RESPIRATORY, THORACIC AND MEDIASTINAL DISORDERS listed in statin SmPCs, subdivided by category component parts

Endpoint	Events (% per annum)		Observed−Expected		Rate Ratio (95% CI)
	Statin (n=62028)	Placebo (n=61912)	O−E	Var(O−E)	
**Blood and lymphatic system disorders**					
Anaemia	1236 (0.5)	1228 (0.5)	3.6	615.9	1.01 (0.93 − 1.09)
Eosinophilia	0 (0.0)	1 (<0.1)	−0.5	0.2	
Thrombocytopaenia	92 (<0.1)	90 (<0.1)	1.0	45.5	1.02 (0.76 − 1.37)
RBC sedimentation rate increased †	50 (<0.1)	60 (<0.1)	−5.0	27.5	0.84 (0.57 − 1.21)
**Immune related disorders**					
Anaphylactic reaction or hypersensitivity	427 (0.2)	431 (0.2)	−2.7	214.5	0.99 (0.86 − 1.13)
Angioedema	179 (<0.1)	185 (<0.1)	−3.6	91.0	0.96 (0.78 − 1.18)
Immune conditions affecting muscle	79 (<0.1)	74 (<0.1)	2.3	38.2	1.06 (0.77 − 1.46)
Lupus−like syndrome	11 (<0.1)	12 (<0.1)	−0.5	5.7	0.92 (0.40 − 2.07)
Serious skin drug reactions	3 (<0.1)	2 (<0.1)	0.5	1.2	1.48 (0.26 − 8.56)
Vasculitis	89 (<0.1)	77 (<0.1)	6.1	41.5	1.16 (0.86 − 1.57)
**Skin and subcutaneous tissue disorders**					
Hair disorder	143 (<0.1)	140 (<0.1)	1.3	70.7	1.02 (0.81 − 1.29)
Dermatitis and eczema	1278 (0.5)	1223 (0.5)	28.1	625.2	1.05 (0.97 − 1.13)
Rash or urticaria	2147 (0.8)	2259 (0.9)	−63.3	1101.3	0.94 (0.89 − 1.00)
Pruritus	1152 (0.4)	1111 (0.4)	19.6	565.7	1.04 (0.95 − 1.12)
Other skin condition	263 (0.1)	228 (<0.1)	17.5	122.7	1.15 (0.97 − 1.38)
Flushing	252 (<0.1)	283 (0.1)	−16.1	133.7	0.89 (0.75 − 1.05)
**Respiratory, thoracic and mediastinal disorders**					
Cough	4009 (1.6)	3895 (1.5)	61.3	1975.4	1.03 (0.99 − 1.08)
Dyspnoea	2946 (1.2)	3016 (1.2)	−40.6	1490.1	0.97 (0.92 − 1.02)
Epistaxis	698 (0.3)	667 (0.3)	14.4	341.2	1.04 (0.94 − 1.16)
Interstitial lung disease	73 (<0.1)	80 (<0.1)	−3.7	38.2	0.91 (0.66 − 1.25)
Laryngeal pain	210 (<0.1)	184 (<0.1)	12.1	98.5	1.13 (0.93 − 1.38)
Nasopharyngitis	973 (0.4)	960 (0.4)	5.1	483.1	1.01 (0.92 − 1.10)

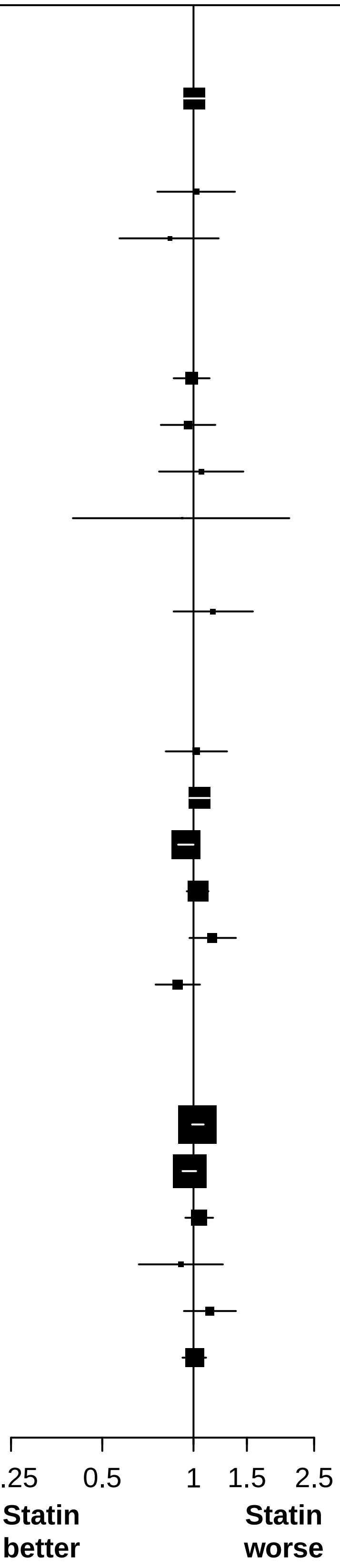

† RBC=Red blood cell

RRs are only plotted for outcomes with at least 10 events, though all outcomes contribute to the shown subtotals and totals

Webfigure 2a: Effect of statin vs placebo on HEPATOBILIARY DISORDERS: ABNORMAL LIVER TRANSAMINASES, subdivided by statin intensity and trial

Trial	Dose	Events (% per annum) Statin (n=62028)	Placebo (n=61912)	Observed−Expected O−E	Var(O−E)	Rate Ratio (95% CI)
**Low− or moderate−intensity statin**						
**Lovastatin**						
AFCAPS/TexCAPS	20−40mg	114 (0.7)	77 (0.5)	18.5	47.7	1.47 (1.11 − 1.96)
**Subtotal**		**114 (0.7)**	**77 (0.5)**	**18.5**	**47.7**	**1.47 (1.11 − 1.96)**
**Fluvastatin**						
ALERT	40−80mg	4 (<0.1)	7 (0.1)	−1.5	2.7	0.58 (0.18 − 1.89)
LIPS	80mg	10 (0.3)	9 (0.3)	0.4	4.7	1.09 (0.44 − 2.68)
**Subtotal**		**14 (0.2)**	**16 (0.2)**	**−1.1**	**7.5**	**0.87 (0.42 − 1.77)**
**Simvastatin**						
4S	20−40mg	247 (2.3)	201 (1.9)	23.7	112.0	1.24 (1.03 − 1.49)
HPS	40mg	−	−	−	−	
**Subtotal**		**247 (0.4)**	**201 (0.3)**	**23.7**	**112.0**	**1.24 (1.03 − 1.49)**
**Pravastatin**						
WOSCOPS	40mg	19 (0.1)	10 (<0.1)	4.4	7.2	1.85 (0.89 − 3.82)
CARE	40mg	22 (0.2)	20 (0.2)	0.9	10.5	1.09 (0.60 − 2.00)
LIPID	40mg	54 (0.2)	37 (0.1)	8.3	22.7	1.44 (0.96 − 2.17)
PROSPER	40mg	−	−	−	−	
**Subtotal**		**95 (0.2)**	**67 (0.1)**	**13.7**	**40.5**	**1.40 (1.03 − 1.91)**
**Atorvastatin**						
ASCOT−LLA	10mg	15 (<0.1)	17 (0.1)	−1.0	8.0	0.88 (0.44 − 1.76)
ASPEN	10mg	10 (0.2)	13 (0.3)	−1.6	5.7	0.75 (0.33 − 1.71)
CARDS	10mg	21 (0.4)	20 (0.3)	0.4	10.2	1.04 (0.56 − 1.91)
4D	20mg	1 (<0.1)	3 (0.2)	−1.1	1.0	0.32 (0.04 − 2.32)
**Subtotal**		**47 (0.2)**	**53 (0.2)**	**−3.4**	**25.0**	**0.87 (0.59 − 1.29)**
**Rosuvastatin**						
CORONA	10mg	14 (0.2)	5 (<0.1)	4.5	4.7	2.57 (1.05 − 6.32)
GISSI−HF	10mg	−	−	−	−	
AURORA	10mg	14 (0.3)	9 (0.2)	2.5	5.7	1.55 (0.68 − 3.51)
HOPE−3	10mg	13 (<0.1)	7 (<0.1)	3.0	5.0	1.81 (0.76 − 4.36)
**Subtotal**		**41 (<0.1)**	**21 (<0.1)**	**10.0**	**15.5**	**1.90 (1.16 − 3.13)**
**Any low− or moderate−intensity**		**558 (0.2)**	**435 (0.2)**	**61.3**	**248.2**	**1.28 (1.13 − 1.45)**
**High−intensity statin**						
**Atorvastatin**						
SPARCL	80mg	111 (1.0)	40 (0.4)	36.4	37.7	2.62 (1.91 − 3.61)
**Subtotal**		**111 (1.0)**	**40 (0.4)**	**36.4**	**37.7**	**2.62 (1.91 − 3.61)**
**Rosuvastatin**						
JUPITER	20mg	114 (0.6)	81 (0.5)	16.5	48.7	1.40 (1.06 − 1.86)
**Subtotal**		**114 (0.6)**	**81 (0.5)**	**16.5**	**48.7**	**1.40 (1.06 − 1.86)**
**Any high−intensity**		**225 (0.8)**	**121 (0.4)**	**52.9**	**86.5**	**1.84 (1.49 − 2.28)**
**All trials**		**783 (0.3)**	**556 (0.2)**	**114.2**	**334.7**	**1.41 (1.26 − 1.57)**

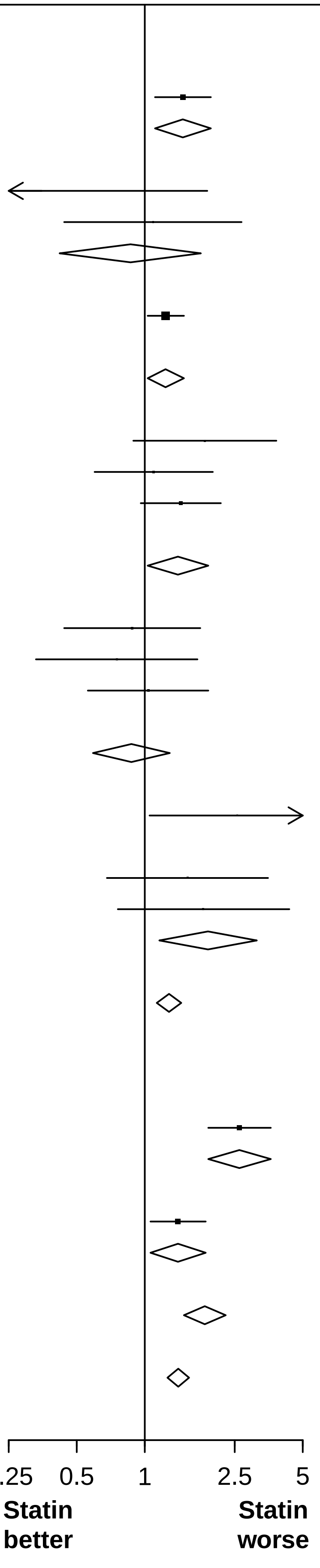

99% or 95% CI

Note: No data is represented by a '−'

Test for heterogeneity between 6 statins in the low− or moderate−intensity analysis: χ^2_5 =8.7, p=0.12

Test for heterogeneity between 2 statins in the high−intensity trials analysis: χ^2_1 =8.3, p=0.0039

Test for heterogeneity between low− or moderate−intensity and high−intensity trials analysis: χ^2_1 =8.5, p=0.0035

RRs are only plotted for outcomes with at least 10 events, though all outcomes contribute to the shown subtotals and totals

Webfigure 2b: Effect of statin vs placebo on HEPATOBLIARY DISORDERS: OTHER LIVER FUNCTION TEST ABNORMALITY, subdivided by statin intensity and trial

Trial	Dose	Events (% per annum) Statin (n=62028)	Events (% per annum) Placebo (n=61912)	Observed−Expected O−E	Observed−Expected Var(O−E)	Rate Ratio (95% CI)
**Low− or moderate−intensity statin**						
**Lovastatin**						
AFCAPS/TexCAPS	20−40mg	64 (0.4)	48 (0.3)	7.8	28.0	1.32 (0.91 − 1.91)
**Subtotal**		**64 (0.4)**	**48 (0.3)**	**7.8**	**28.0**	**1.32 (0.91 − 1.91)**
**Fluvastatin**						
ALERT	40−80mg	26 (0.5)	18 (0.3)	4.1	11.0	1.45 (0.80 − 2.62)
LIPS	80mg	5 (0.2)	2 (<0.1)	1.5	1.7	2.32 (0.53 − 10.21)
**Subtotal**		**31 (0.4)**	**20 (0.2)**	**5.6**	**12.7**	**1.55 (0.89 − 2.68)**
**Simvastatin**						
4S	20−40mg	12 (0.1)	11 (<0.1)	0.4	5.7	1.08 (0.48 − 2.44)
HPS	40mg	17 (<0.1)	22 (<0.1)	−2.6	9.7	0.77 (0.41 − 1.44)
**Subtotal**		**29 (<0.1)**	**33 (<0.1)**	**−2.1**	**15.5**	**0.87 (0.53 − 1.43)**
**Pravastatin**						
WOSCOPS	40mg	14 (<0.1)	22 (0.1)	−4.1	9.0	0.63 (0.33 − 1.22)
CARE	40mg	98 (1.0)	110 (1.1)	−6.5	52.0	0.88 (0.67 − 1.16)
LIPID	40mg	26 (0.1)	16 (<0.1)	4.9	10.5	1.60 (0.87 − 2.92)
PROSPER	40mg	85 (0.9)	73 (0.8)	6.4	39.5	1.17 (0.86 − 1.61)
**Subtotal**		**223 (0.4)**	**221 (0.4)**	**0.7**	**111.0**	**1.01 (0.84 − 1.21)**
**Atorvastatin**						
ASCOT−LLA	10mg	24 (0.1)	22 (0.1)	0.9	11.5	1.09 (0.61 − 1.93)
ASPEN	10mg	20 (0.5)	22 (0.5)	−1.3	10.5	0.88 (0.48 − 1.61)
CARDS	10mg	13 (0.2)	20 (0.3)	−3.6	8.2	0.64 (0.33 − 1.28)
4D	20mg	9 (0.5)	6 (0.3)	1.6	3.7	1.53 (0.56 − 4.20)
**Subtotal**		**66 (0.2)**	**70 (0.2)**	**−2.4**	**34.0**	**0.93 (0.67 − 1.30)**
**Rosuvastatin**						
CORONA	10mg	15 (0.2)	11 (0.2)	1.9	6.5	1.35 (0.63 − 2.91)
GISSI−HF	10mg	31 (0.4)	18 (0.2)	6.6	12.2	1.71 (0.98 − 3.00)
AURORA	10mg	12 (0.3)	14 (0.3)	−1.0	6.5	0.86 (0.40 − 1.85)
HOPE−3	10mg	7 (<0.1)	9 (<0.1)	−1.0	4.0	0.77 (0.29 − 2.06)
**Subtotal**		**65 (0.1)**	**52 (<0.1)**	**6.5**	**29.2**	**1.25 (0.87 − 1.80)**
**Any low− or moderate−intensity**		**478 (0.2)**	**444 (0.2)**	**16.0**	**230.5**	**1.07 (0.94 − 1.22)**
**High−intensity statin**						
**Atorvastatin**						
SPARCL	80mg	120 (1.1)	33 (0.3)	44.6	38.2	3.21 (2.34 − 4.40)
**Subtotal**		**120 (1.1)**	**33 (0.3)**	**44.6**	**38.2**	**3.21 (2.34 − 4.40)**
**Rosuvastatin**						
JUPITER	20mg	53 (0.3)	41 (0.2)	6.0	23.5	1.29 (0.86 − 1.93)
**Subtotal**		**53 (0.3)**	**41 (0.2)**	**6.0**	**23.5**	**1.29 (0.86 − 1.93)**
**Any high−intensity**		**173 (0.6)**	**74 (0.3)**	**50.5**	**61.7**	**2.27 (1.77 − 2.91)**
**All trials**		**651 (0.3)**	**518 (0.2)**	**66.6**	**292.2**	**1.26 (1.12 − 1.41)**

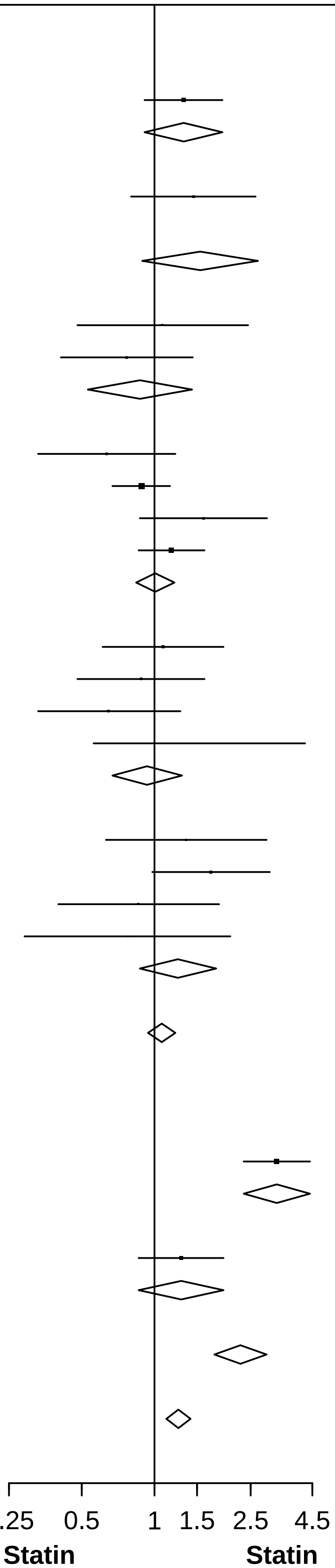

99% or 95% CI

Test for heterogeneity between 6 statins in the low− or moderate−intensity analysis: χ^2_5 =5.4, p=0.37

Test for heterogeneity between 2 statins in the high−intensity trials analysis: χ^2_1 =12.1, p=0.00051

Test for heterogeneity between low− or moderate−intensity and high−intensity trials analysis: χ^2_1 =27.3, p<0.0001

RRs are only plotted for outcomes with at least 10 events, though all outcomes contribute to the shown subtotals and totals

Webfigure 2c: Effect of statin vs placebo on RENAL AND URINARY DISORDERS: URINARY COMPOSITION ALTERATION, subdivided by statin intensity and trial

Trial	Dose	Events (% per annum) Statin (n=62028)	Events (% per annum) Placebo (n=61912)	Observed−Expected O−E	Observed−Expected Var(O−E)	Rate Ratio (95% CI)
**Low− or moderate−intensity statin**						
**Lovastatin**						
AFCAPS/TexCAPS	20−40mg	−	−	−	−	
**Subtotal**		**−**	**−**	**−**	**−**	
**Fluvastatin**						
ALERT	40−80mg	37 (0.7)	29 (0.5)	4.1	16.5	1.28 (0.79 − 2.08)
LIPS	80mg	1 (<0.1)	0 (0.0)	0.5	0.2	
**Subtotal**		**38 (0.5)**	**29 (0.3)**	**4.6**	**16.7**	**1.31 (0.81 − 2.12)**
**Simvastatin**						
4S	20−40mg	9 (<0.1)	0 (0.0)	4.5	2.2	7.30 (1.98 − 26.98)
HPS	40mg	1 (<0.1)	2 (<0.1)	−0.5	0.7	0.51 (0.05 − 4.92)
**Subtotal**		**10 (<0.1)**	**2 (<0.1)**	**4.0**	**3.0**	**3.76 (1.21 − 11.65)**
**Pravastatin**						
WOSCOPS	40mg	1 (<0.1)	4 (<0.1)	−1.5	1.2	0.30 (0.05 − 1.71)
CARE	40mg	103 (1.0)	60 (0.6)	21.7	40.7	1.70 (1.25 − 2.31)
LIPID	40mg	−	−	−	−	
PROSPER	40mg	180 (2.0)	175 (1.9)	3.2	88.7	1.04 (0.84 − 1.28)
**Subtotal**		**284 (0.5)**	**239 (0.4)**	**23.3**	**130.7**	**1.20 (1.01 − 1.42)**
**Atorvastatin**						
ASCOT−LLA	10mg	21 (0.1)	19 (0.1)	0.9	10.0	1.10 (0.59 − 2.04)
ASPEN	10mg	36 (0.8)	35 (0.8)	−0.2	17.7	0.99 (0.62 − 1.58)
CARDS	10mg	46 (0.8)	28 (0.5)	8.9	18.5	1.62 (1.03 − 2.55)
4D	20mg	−	−	−	−	
**Subtotal**		**103 (0.4)**	**82 (0.3)**	**9.6**	**46.2**	**1.23 (0.92 − 1.64)**
**Rosuvastatin**						
CORONA	10mg	0 (0.0)	1 (<0.1)	−0.5	0.2	
GISSI−HF	10mg	−	−	−	−	
AURORA	10mg	0 (0.0)	1 (<0.1)	−0.5	0.2	
HOPE−3	10mg	2 (<0.1)	1 (<0.1)	0.5	0.7	1.92 (0.20 − 18.42)
**Subtotal**		**2 (<0.1)**	**3 (<0.1)**	**−0.5**	**1.2**	**0.67 (0.12 − 3.84)**
**Any low− or moderate−intensity**		**437 (0.2)**	**355 (0.2)**	**41.0**	**198.0**	**1.23 (1.07 − 1.41)**
**High−intensity statin**						
**Atorvastatin**						
SPARCL	80mg	3 (<0.1)	0 (0.0)	1.5	0.7	7.45 (0.77 − 71.62)
**Subtotal**		**3 (<0.1)**	**0 (0.0)**	**1.5**	**0.7**	**7.45 (0.77 − 71.62)**
**Rosuvastatin**						
JUPITER	20mg	116 (0.6)	117 (0.7)	−0.6	58.2	0.99 (0.77 − 1.28)
**Subtotal**		**116 (0.6)**	**117 (0.7)**	**−0.6**	**58.2**	**0.99 (0.77 − 1.28)**
**Any high−intensity**		**119 (0.4)**	**117 (0.4)**	**0.9**	**59.0**	**1.02 (0.79 − 1.31)**
**All trials**		**556 (0.2)**	**472 (0.2)**	**41.9**	**256.9**	**1.18 (1.04 − 1.33)**

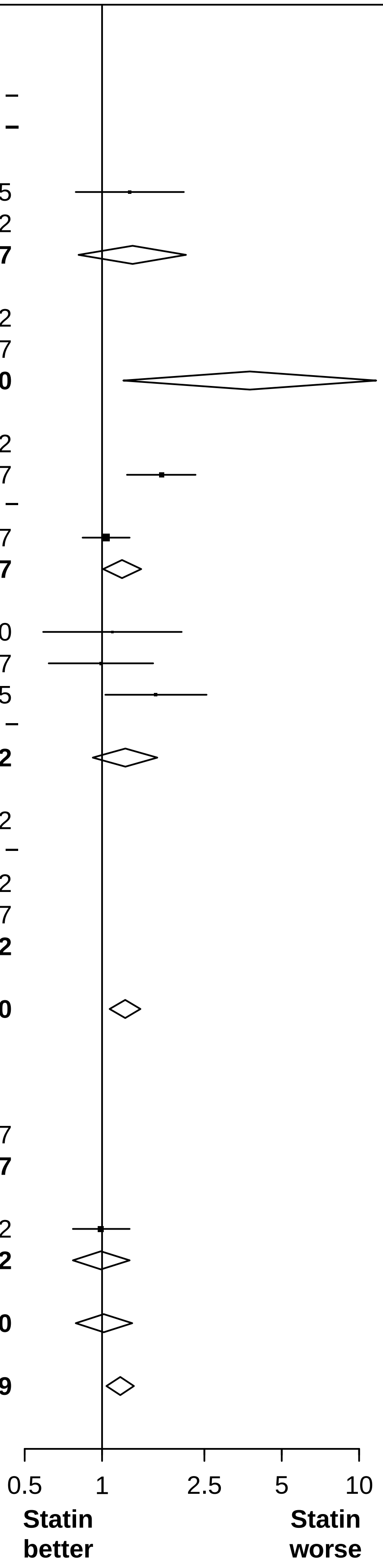

■ 99% or ◇ 95% CI

Note: No data is represented by a '−'

Test for heterogeneity between 5 statins in the low− or moderate−intensity analysis: χ^2_4 =4.4, p=0.36

Test for heterogeneity between 2 statins in the high−intensity trials analysis: χ^2_1 =3.0, p=0.082

Test for heterogeneity between low− or moderate−intensity and high−intensity trials analysis: χ^2_1 =1.7, p=0.20

RRs are only plotted for outcomes with at least 10 events, though all outcomes contribute to the shown subtotals and totals

Webfigure 2d: Effect of statin vs placebo on GENERAL DISORDERS AND ADMINISTRATION SITE CONDITIONS: OEDEMA, subdivided by statin intensity and trial

Trial	Dose	Events (% per annum) Statin (n=62028)	Events (% per annum) Placebo (n=61912)	Observed−Expected O−E	Observed−Expected Var(O−E)	Rate Ratio (95% CI)
**Low− or moderate−intensity statin**						
**Lovastatin**						
AFCAPS/TexCAPS	20−40mg	6 (<0.1)	4 (<0.1)	1.0	2.5	1.49 (0.43 − 5.13)
**Subtotal**		**6 (<0.1)**	**4 (<0.1)**	**1.0**	**2.5**	**1.49 (0.43 − 5.13)**
**Fluvastatin**						
ALERT	40−80mg	227 (4.9)	227 (4.8)	0.7	113.5	1.01 (0.84 − 1.21)
LIPS	80mg	36 (1.2)	27 (0.9)	4.2	15.7	1.31 (0.80 − 2.14)
**Subtotal**		**263 (3.4)**	**254 (3.3)**	**5.0**	**129.2**	**1.04 (0.87 − 1.23)**
**Simvastatin**						
4S	20−40mg	59 (0.5)	54 (0.5)	2.3	28.2	1.08 (0.75 − 1.57)
HPS	40mg	21 (<0.1)	27 (<0.1)	−3.1	12.0	0.77 (0.44 − 1.36)
**Subtotal**		**80 (0.1)**	**81 (0.1)**	**−0.8**	**40.2**	**0.98 (0.72 − 1.34)**
**Pravastatin**						
WOSCOPS	40mg	40 (0.3)	29 (0.2)	5.4	17.2	1.36 (0.85 − 2.19)
CARE	40mg	259 (2.8)	232 (2.5)	13.4	122.7	1.12 (0.93 − 1.33)
LIPID	40mg	6 (<0.1)	6 (<0.1)	0.0	3.0	0.99 (0.32 − 3.06)
PROSPER	40mg	449 (5.2)	452 (5.2)	0.0	225.2	1.00 (0.88 − 1.14)
**Subtotal**		**754 (1.3)**	**719 (1.2)**	**18.7**	**368.2**	**1.05 (0.95 − 1.17)**
**Atorvastatin**						
ASCOT−LLA	10mg	718 (4.9)	666 (4.5)	26.3	345.8	1.08 (0.97 − 1.20)
ASPEN	10mg	109 (2.6)	95 (2.3)	6.3	50.9	1.13 (0.86 − 1.49)
CARDS	10mg	123 (2.2)	116 (2.1)	2.9	59.7	1.05 (0.81 − 1.35)
4D	20mg	4 (0.2)	1 (<0.1)	1.5	1.2	3.34 (0.58 − 19.30)
**Subtotal**		**954 (3.6)**	**878 (3.3)**	**37.0**	**457.7**	**1.08 (0.99 − 1.19)**
**Rosuvastatin**						
CORONA	10mg	82 (1.3)	82 (1.4)	−0.4	41.0	0.99 (0.73 − 1.35)
GISSI−HF	10mg	860 (15.2)	822 (14.0)	31.4	418.3	1.08 (0.98 − 1.19)
AURORA	10mg	71 (1.7)	79 (1.9)	−4.1	37.5	0.90 (0.65 − 1.24)
HOPE−3	10mg	10 (<0.1)	13 (<0.1)	−1.6	5.7	0.76 (0.34 − 1.73)
**Subtotal**		**1023 (2.0)**	**996 (1.9)**	**25.4**	**502.5**	**1.05 (0.96 − 1.15)**
**Any low− or moderate−intensity**		**3080 (1.4)**	**2932 (1.3)**	**86.3**	**1500.4**	**1.06 (1.01 − 1.11)**
**High−intensity statin**						
**Atorvastatin**						
SPARCL	80mg	132 (1.2)	118 (1.1)	7.5	62.5	1.13 (0.88 − 1.44)
**Subtotal**		**132 (1.2)**	**118 (1.1)**	**7.5**	**62.5**	**1.13 (0.88 − 1.44)**
**Rosuvastatin**						
JUPITER	20mg	283 (1.6)	249 (1.4)	17.0	133.0	1.14 (0.96 − 1.35)
**Subtotal**		**283 (1.6)**	**249 (1.4)**	**17.0**	**133.0**	**1.14 (0.96 − 1.35)**
**Any high−intensity**		**415 (1.5)**	**367 (1.3)**	**24.6**	**195.5**	**1.13 (0.99 − 1.30)**
**All trials**		**3495 (1.4)**	**3299 (1.3)**	**110.8**	**1695.9**	**1.07 (1.02 − 1.12)**

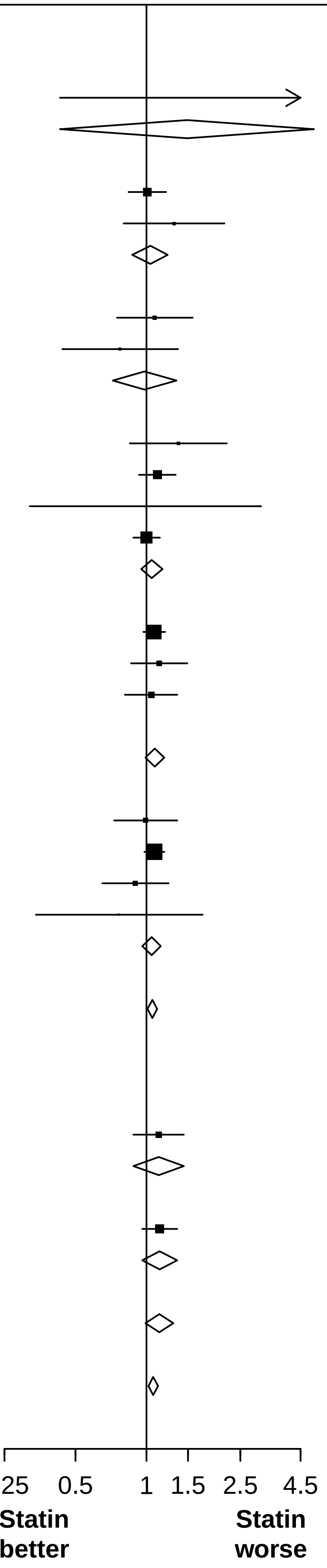

■ 99% or ◇ 95% CI

Test for heterogeneity between 6 statins in the low− or moderate−intensity analysis: χ^2_5 =0.9, p=0.97

Test for heterogeneity between 2 statins in the high−intensity trials analysis: χ^2_1 =0.1, p=0.96

Test for heterogeneity between low− or moderate−intensity and high−intensity trials analysis: χ^2_1 =0.8, p=0.37

RRs are only plotted for outcomes with at least 10 events, though all outcomes contribute to the shown subtotals and totals

Webfigure 3a: Effect of statin vs placebo on HEPATOBILIARY DISORDERS: ABNORMAL LIVER TRANSAMINASES, subdivided by baseline characteristics

Subgroup	Events (% per annum) Statin (n=62028)	Events (% per annum) Placebo (n=61912)	Observed−Expected O−E	Observed−Expected Var(O−E)	Rate Ratio (95% CI)
**Age, years** (Trend: χ^2_1 =10.8, p=0.0010)					
≤65	591 (0.4)	458 (0.3)	70.3	262.1	1.31 (1.16 − 1.48)
>65, ≤75	159 (0.2)	91 (0.1)	31.6	62.4	1.66 (1.30 − 2.13)
>75	33 (0.2)	7 (<0.1)	13.2	10.0	3.76 (2.02 − 7.00)
**Sex** (Heterogeneity: χ^2_1 =1.7, p=0.19)					
Female	167 (0.2)	104 (0.1)	32.8	67.7	1.62 (1.28 − 2.06)
Male	616 (0.3)	452 (0.2)	81.4	266.9	1.36 (1.20 − 1.53)
**Race/Ethnicity** (Heterogeneity: χ^2_3 =3.4, p=0.33)					
Asian	14 (<0.1)	4 (<0.1)	5.0	4.5	3.07 (1.22 − 7.75)
Black	5 (0.2)	6 (0.2)	−0.5	2.7	0.83 (0.25 − 2.73)
Caucasian	277 (0.2)	188 (0.1)	45.0	116.2	1.47 (1.23 − 1.77)
Other	25 (0.2)	15 (0.1)	5.0	10.0	1.65 (0.89 − 3.07)
Missing	462 (0.5)	343 (0.4)	59.9	201.2	1.35 (1.17 − 1.55)
**History of vascular disease** (Heterogeneity: χ^2_1 =0.3, p=0.61)					
No	313 (0.2)	229 (0.2)	41.6	135.4	1.36 (1.15 − 1.61)
Yes	470 (0.4)	327 (0.2)	72.4	199.2	1.44 (1.25 − 1.65)
**Body mass index, kg/m^2** (Trend: χ^2_1 =9.5, p=0.0021)					
≤25	226 (0.3)	144 (0.2)	42.7	92.5	1.59 (1.29 − 1.95)
>25, ≤30	332 (0.3)	238 (0.2)	45.3	142.4	1.37 (1.17 − 1.62)
>30	140 (0.2)	143 (0.3)	−2.5	70.7	0.96 (0.76 − 1.22)
**Estimated GFR, mL/min/1.73m^2** (Trend: χ^2_1 =0.3, p=0.61)					
On dialysis	15 (0.2)	12 (0.2)	1.4	6.7	1.23 (0.58 − 2.62)
≤60	137 (0.2)	69 (0.1)	34.8	51.5	1.97 (1.50 − 2.59)
>60, ≤90	309 (0.2)	231 (0.2)	37.9	135.0	1.32 (1.12 − 1.57)
>90	69 (0.2)	37 (0.1)	16.6	26.5	1.88 (1.28 − 2.75)
**LDL−C, mmol/L** (Trend: χ^2_1 =0.5, p=0.46)					
≤3.0	155 (0.2)	102 (0.1)	26.4	64.2	1.51 (1.18 − 1.93)
>3.0, ≤4.0	276 (0.3)	189 (0.2)	42.8	116.2	1.45 (1.21 − 1.73)
>4.0	348 (0.4)	258 (0.3)	46.8	151.4	1.36 (1.16 − 1.60)
**Total**	**783 (0.3)**	**556 (0.2)**	**114.2**	**334.7**	**1.41 (1.26 − 1.57)**

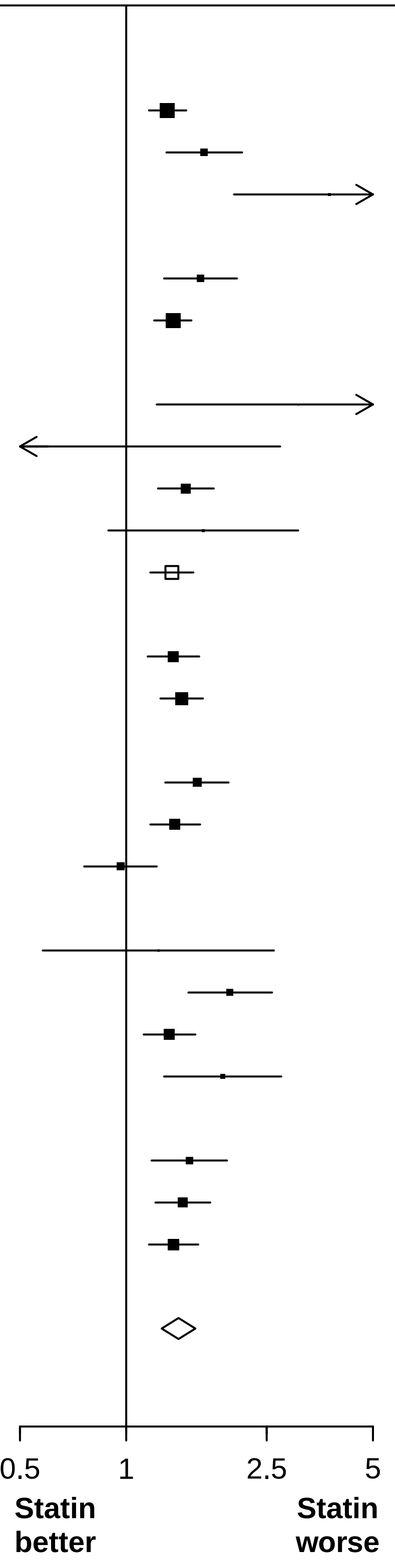

Webfigure 3b: Effect of statin vs placebo on HEPATOBILIARY DISORDERS: OTHER LIVER FUNCTION TEST ABNORMALITY, subdivided by baseline characteristics

Subgroup	Events (% per annum) Statin (n=62028)	Events (% per annum) Placebo (n=61912)	Observed−Expected O−E	Observed−Expected Var(O−E)	Rate Ratio (95% CI)
**Age, years** (Trend: χ_1^2 =0.3, p=0.56)					
≤65	372 (0.2)	300 (0.2)	36.7	167.9	1.24 (1.07 − 1.45)
>65, ≤75	194 (0.2)	160 (0.2)	16.5	88.4	1.20 (0.98 − 1.48)
>75	85 (0.5)	58 (0.3)	13.6	35.7	1.46 (1.05 − 2.03)
**Sex** (Heterogeneity: χ_1^2 =4.4, p=0.037)					
Female	227 (0.3)	152 (0.2)	38.4	94.7	1.50 (1.23 − 1.83)
Male	424 (0.2)	366 (0.2)	28.4	197.5	1.15 (1.00 − 1.33)
**Race/Ethnicity** (Heterogeneity: χ_3^2 =1.1, p=0.77)					
Asian	8 (<0.1)	8 (<0.1)	−0.1	4.0	0.97 (0.36 − 2.60)
Black	14 (0.5)	12 (0.4)	0.7	6.5	1.11 (0.51 − 2.40)
Caucasian	389 (0.3)	286 (0.2)	51.4	168.7	1.36 (1.17 − 1.58)
Other	23 (0.2)	22 (0.2)	0.9	11.2	1.08 (0.60 − 1.94)
Missing	217 (0.2)	190 (0.2)	13.5	101.7	1.14 (0.94 − 1.39)
**History of vascular disease** (Heterogeneity: χ_1^2 =4.1, p=0.042)					
No	255 (0.2)	235 (0.2)	10.6	122.5	1.09 (0.91 − 1.30)
Yes	396 (0.3)	283 (0.2)	55.5	169.7	1.39 (1.19 − 1.61)
**Body mass index, kg/m^2** (Trend: χ_1^2 =0.1, p=0.72)					
≤25	188 (0.2)	157 (0.2)	16.6	86.2	1.21 (0.98 − 1.50)
>25, ≤30	247 (0.2)	220 (0.2)	13.0	116.7	1.12 (0.93 − 1.34)
>30	141 (0.2)	120 (0.2)	9.1	65.1	1.15 (0.90 − 1.47)
**Estimated GFR, mL/min/1.73m^2** (Trend: χ_1^2 =0.1, p=0.70)					
On dialysis	21 (0.3)	20 (0.3)	0.6	10.2	1.06 (0.57 − 1.95)
≤60	238 (0.4)	182 (0.3)	28.6	105.0	1.31 (1.08 − 1.59)
>60, ≤90	309 (0.2)	261 (0.2)	23.8	142.5	1.18 (1.00 − 1.39)
>90	59 (0.2)	36 (0.1)	11.0	23.7	1.59 (1.06 − 2.38)
**LDL−C, mmol/L** (Trend: χ_1^2 =0.4, p=0.52)					
≤3.0	176 (0.3)	125 (0.2)	26.0	75.2	1.41 (1.13 − 1.77)
>3.0, ≤4.0	308 (0.3)	263 (0.3)	22.5	142.7	1.17 (0.99 − 1.38)
>4.0	158 (0.2)	124 (0.2)	17.2	70.4	1.28 (1.01 − 1.61)
**Total**	**651 (0.3)**	**518 (0.2)**	**66.6**	**292.2**	**1.26 (1.12 − 1.41)**

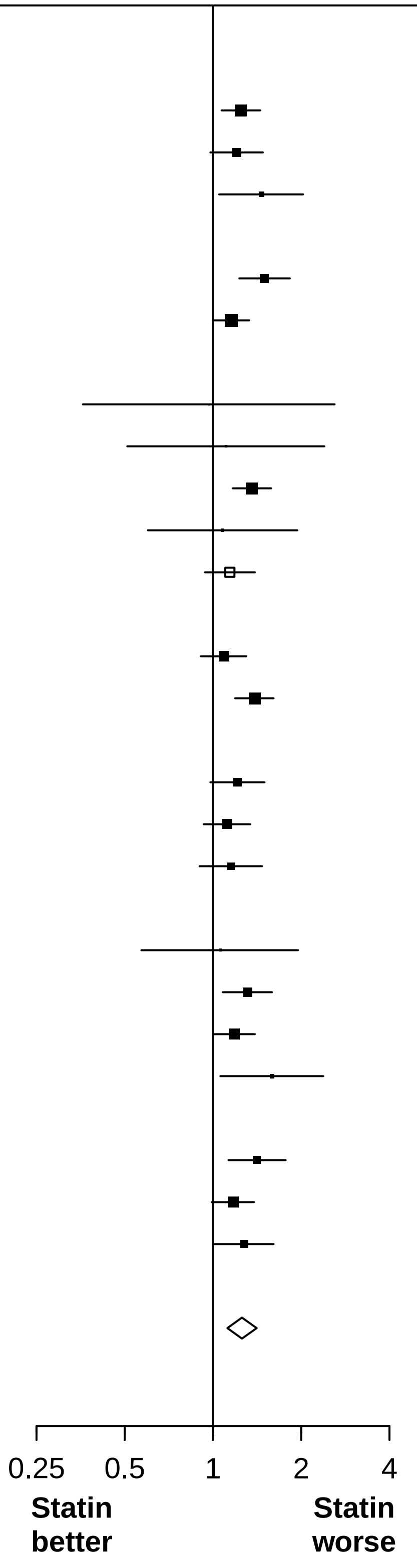

Webfigure 3c: Effect of statin vs placebo on RENAL AND URINARY DISORDERS: URINARY COMPOSITION ALTERATION, subdivided by baseline characteristics

Subgroup	Events (% per annum) Statin (n=62028)	Events (% per annum) Placebo (n=61912)	Observed−Expected O−E	Observed−Expected Var(O−E)	Rate Ratio (95% CI)
**Age, years** (Trend: χ^2_1 =1.4, p=0.23)					
≤65	231 (0.1)	180 (0.1)	26.0	102.7	1.29 (1.06 − 1.56)
>65, ≤75	218 (0.2)	192 (0.2)	12.9	102.4	1.13 (0.93 − 1.38)
>75	107 (0.6)	100 (0.6)	3.2	51.7	1.06 (0.81 − 1.40)
**Sex** (Heterogeneity: χ^2_1 =0.9, p=0.33)					
Female	152 (0.2)	141 (0.2)	5.0	73.2	1.07 (0.85 − 1.35)
Male	404 (0.2)	331 (0.2)	37.1	183.7	1.22 (1.06 − 1.41)
**Race/Ethnicity** (Heterogeneity: χ^2_3 =2.6, p=0.46)					
Asian	3 (<0.1)	4 (<0.1)	−0.9	1.7	0.59 (0.13 − 2.67)
Black	8 (0.3)	5 (0.2)	1.3	3.2	1.52 (0.51 − 4.53)
Caucasian	229 (0.2)	161 (0.1)	33.2	97.5	1.41 (1.15 − 1.71)
Other	10 (<0.1)	4 (<0.1)	3.3	3.5	2.58 (0.90 − 7.35)
Missing	306 (0.3)	298 (0.3)	4.5	151.0	1.03 (0.88 − 1.21)
**History of vascular disease** (Heterogeneity: χ^2_1 =6.2, p=0.013)					
No	331 (0.3)	320 (0.3)	7.4	162.7	1.05 (0.90 − 1.22)
Yes	225 (0.2)	152 (0.1)	34.6	94.2	1.44 (1.18 − 1.77)
**Body mass index, kg/m^2** (Trend: χ^2_1 =0.2, p=0.66)					
≤25	163 (0.2)	132 (0.2)	16.9	73.7	1.26 (1.00 − 1.58)
>25, ≤30	232 (0.2)	203 (0.2)	12.4	108.7	1.12 (0.93 − 1.35)
>30	158 (0.3)	136 (0.2)	11.5	73.4	1.17 (0.93 − 1.47)
**Estimated GFR, mL/min/1.73m^2** (Trend: χ^2_1 =0.7, p=0.39)					
On dialysis	0 (0.0)	1 (<0.1)	−0.5	0.2	
≤60	250 (0.4)	204 (0.3)	22.3	113.5	1.22 (1.01 − 1.46)
>60, ≤90	268 (0.2)	237 (0.2)	16.1	126.1	1.14 (0.95 − 1.35)
>90	22 (<0.1)	24 (<0.1)	−1.4	11.4	0.88 (0.49 − 1.58)
**LDL−C, mmol/L** (Trend: χ^2_1 =1.5, p=0.23)					
≤3.0	160 (0.2)	146 (0.2)	6.2	76.4	1.08 (0.87 − 1.36)
>3.0, ≤4.0	269 (0.3)	234 (0.2)	19.2	125.7	1.16 (0.98 − 1.39)
>4.0	121 (0.2)	86 (0.1)	15.8	51.7	1.36 (1.03 − 1.78)
**Total**	**556 (0.2)**	**472 (0.2)**	**41.9**	**256.9**	**1.18 (1.04 − 1.33)**

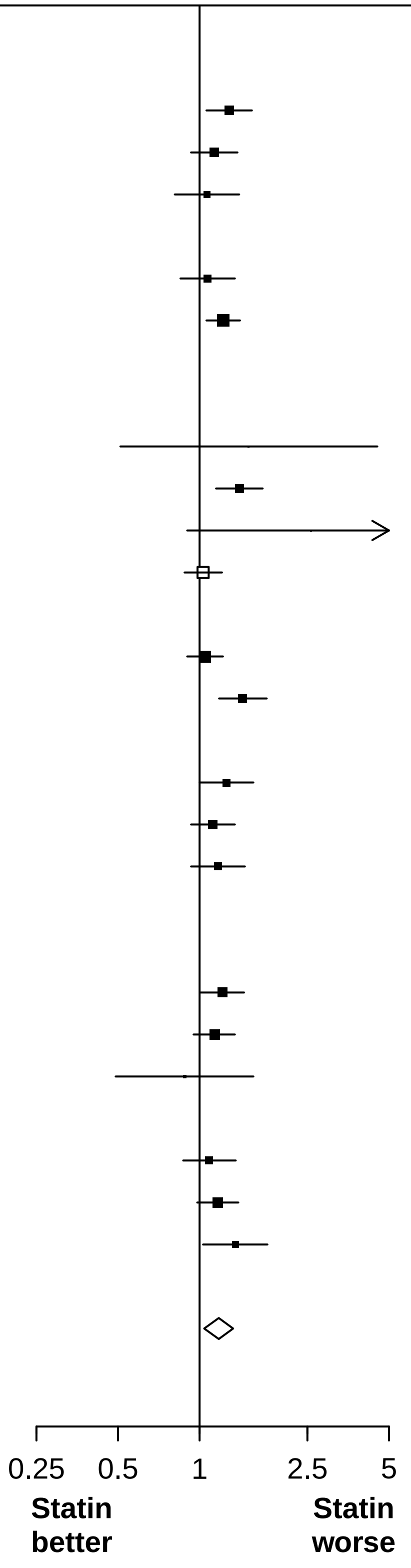

Webfigure 3d: Effect of statin vs placebo on GENERAL DISORDERS AND ADMINISTRATION SITE CONDITIONS: OEDEMA, subdivided by baseline characteristics

Subgroup	Events (% per annum) Statin (n=62028)	Events (% per annum) Placebo (n=61912)	Observed−Expected O−E	Observed−Expected Var(O−E)	Rate Ratio (95% CI)
**Age, years** (Trend: χ^2_1 =0.1, p=0.98)					
≤65	1377 (0.9)	1301 (0.9)	41.5	668.9	1.06 (0.99 − 1.15)
>65, ≤75	1369 (1.6)	1255 (1.5)	49.0	654.4	1.08 (1.00 − 1.16)
>75	746 (4.6)	737 (4.5)	21.1	368.7	1.06 (0.96 − 1.17)
**Sex** (Heterogeneity: χ^2_1 =0.1, p=0.76)					
Female	1309 (1.9)	1224 (1.8)	34.3	632.0	1.06 (0.98 − 1.14)
Male	2186 (1.2)	2075 (1.1)	74.1	1063.3	1.07 (1.01 − 1.14)
**Race/Ethnicity** (Heterogeneity: χ^2_3 =2.0, p=0.57)					
Asian	15 (<0.1)	21 (0.1)	−3.4	8.9	0.69 (0.36 − 1.32)
Black	36 (1.3)	31 (1.1)	2.2	16.7	1.14 (0.71 − 1.85)
Caucasian	2432 (1.9)	2271 (1.8)	92.3	1173.2	1.08 (1.02 − 1.15)
Other	22 (0.2)	25 (0.2)	−0.2	11.4	0.98 (0.55 − 1.75)
Missing	990 (1.1)	951 (1.1)	20.2	485.2	1.04 (0.95 − 1.14)
**History of vascular disease** (Heterogeneity: χ^2_1 =0.1, p=0.77)					
No	1571 (1.3)	1494 (1.2)	43.5	765.8	1.06 (0.99 − 1.14)
Yes	1924 (1.5)	1805 (1.4)	66.0	929.7	1.07 (1.01 − 1.14)
**Body mass index, kg/m^2** (Trend: χ^2_1 =1.2, p=0.27)					
≤25	818 (1.1)	776 (1.0)	25.4	398.0	1.07 (0.97 − 1.18)
>25, ≤30	1334 (1.2)	1316 (1.2)	9.0	661.1	1.01 (0.94 − 1.09)
>30	1257 (2.3)	1138 (2.1)	72.7	596.6	1.13 (1.04 − 1.22)
**Estimated GFR, mL/min/1.73m^2** (Trend: χ^2_1 =5.5, p=0.019)					
On dialysis	75 (1.3)	80 (1.3)	−2.6	38.7	0.94 (0.68 − 1.28)
≤60	1416 (2.3)	1397 (2.3)	9.5	701.8	1.01 (0.94 − 1.09)
>60, ≤90	1519 (1.1)	1389 (1.0)	76.3	725.7	1.11 (1.03 − 1.19)
>90	185 (0.6)	150 (0.5)	17.7	83.3	1.24 (1.00 − 1.53)
**LDL−C, mmol/L** (Trend: χ^2_1 =0.1, p=0.81)					
≤3.0	1142 (1.7)	1106 (1.7)	22.4	560.5	1.04 (0.96 − 1.13)
>3.0, ≤4.0	1457 (1.4)	1315 (1.3)	80.4	691.9	1.12 (1.04 − 1.21)
>4.0	709 (0.9)	704 (0.9)	1.9	352.7	1.01 (0.91 − 1.12)
**Total**	**3495 (1.4)**	**3299 (1.3)**	**110.8**	**1695.9**	**1.07 (1.02 − 1.12)**

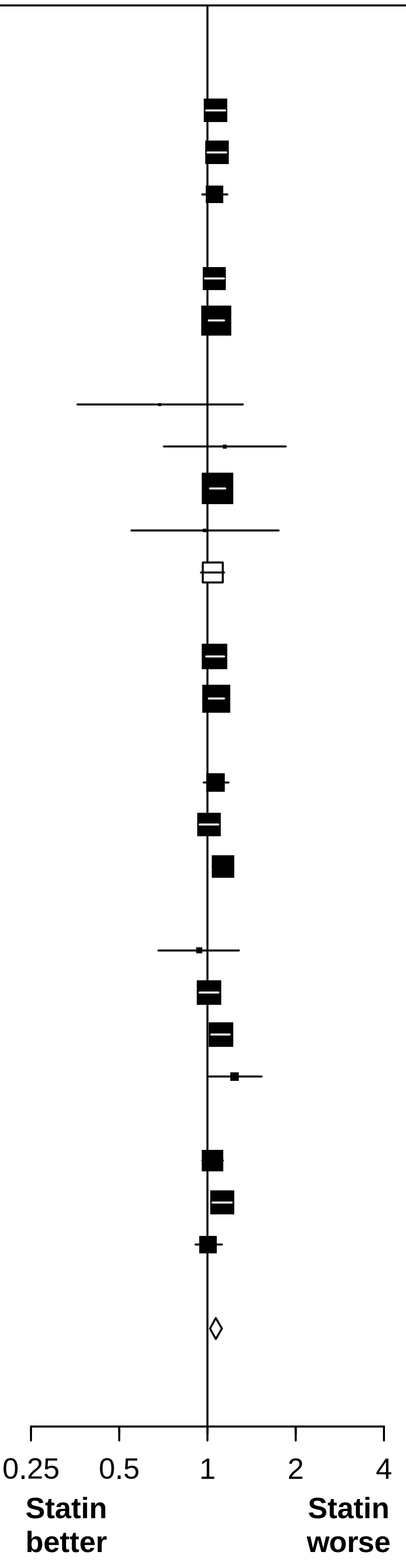

Webfigure 4a: Effect of statin vs placebo on HEPATOBILIARY DISORDERS: ABNORMAL LIVER TRANSAMINASES, subdivided by duration of follow−up

Year	Events (% per annum) Statin	Events (% per annum) Placebo	Observed−Expected O−E	Observed−Expected Var(O−E)	Rate Ratio (95% CI)
**Low− or moderate−intensity statin**	**(n=51288)**	**(n=51207)**			
0−3 months	88 (0.7)	58 (0.5)	15.0	36.5	1.51 (1.09 − 2.09)
3−6 months	56 (0.4)	38 (0.3)	9.1	23.5	1.47 (0.98 − 2.20)
6−9 months	43 (0.3)	40 (0.3)	1.6	20.7	1.08 (0.70 − 1.66)
9−12 months	42 (0.3)	34 (0.3)	4.1	19.0	1.24 (0.79 − 1.94)
**Year 1**	**229 (0.5)**	**170 (0.3)**	**29.7**	**99.7**	**1.35 (1.11 − 1.64)**
Year 2	115 (0.2)	86 (0.2)	14.7	50.2	1.34 (1.02 − 1.77)
Year 3	78 (0.2)	70 (0.2)	4.0	37.0	1.11 (0.81 − 1.54)
Year 4	72 (0.2)	57 (0.1)	7.3	32.2	1.26 (0.89 − 1.77)
Years 5+	64 (0.1)	52 (0.1)	5.6	29.0	1.21 (0.84 − 1.74)
**All after year 1**	**329 (0.2)**	**265 (0.1)**	**31.6**	**148.5**	**1.24 (1.05 − 1.45)**
**All years**	**558 (0.2)**	**435 (0.2)**	**61.3**	**248.2**	**1.28 (1.13 − 1.45)**
**High−intensity statin**	**(n=10740)**	**(n=10705)**			
0−3 months	64 (2.4)	12 (0.5)	26.1	19.0	3.96 (2.52 − 6.20)
3−6 months	53 (2.0)	28 (1.1)	12.6	20.2	1.86 (1.21 − 2.88)
6−9 months	28 (1.1)	20 (0.8)	4.1	12.0	1.40 (0.80 − 2.47)
9−12 months	12 (0.5)	6 (0.2)	3.0	4.5	1.96 (0.78 − 4.95)
**Year 1**	**157 (1.5)**	**66 (0.6)**	**45.8**	**55.7**	**2.28 (1.75 − 2.96)**
Year 2	44 (0.5)	28 (0.3)	8.2	18.0	1.58 (0.99 − 2.50)
Year 3	14 (0.3)	14 (0.3)	0.2	7.0	1.02 (0.49 − 2.15)
Year 4	6 (0.2)	7 (0.2)	−0.4	3.2	0.88 (0.30 − 2.61)
Years 5+	4 (0.2)	6 (0.3)	−0.9	2.5	0.70 (0.20 − 2.40)
**All after year 1**	**68 (0.4)**	**55 (0.3)**	**7.0**	**30.7**	**1.26 (0.88 − 1.79)**
**All years**	**225 (0.8)**	**121 (0.4)**	**52.9**	**86.5**	**1.84 (1.49 − 2.28)**
**Any statin vs. placebo**	**(n=62028)**	**(n=61912)**			
0−3 months	152 (1.0)	70 (0.5)	41.1	55.5	2.10 (1.61 − 2.73)
3−6 months	109 (0.7)	66 (0.4)	21.7	43.7	1.64 (1.22 − 2.21)
6−9 months	71 (0.5)	60 (0.4)	5.7	32.7	1.19 (0.84 − 1.68)
9−12 months	54 (0.4)	40 (0.3)	7.1	23.5	1.35 (0.90 − 2.03)
**Year 1**	**386 (0.6)**	**236 (0.4)**	**75.6**	**155.4**	**1.63 (1.39 − 1.90)**
Year 2	159 (0.3)	114 (0.2)	22.9	68.2	1.40 (1.10 − 1.77)
Year 3	92 (0.2)	84 (0.2)	4.2	44.0	1.10 (0.82 − 1.48)
Year 4	78 (0.2)	64 (0.2)	6.9	35.5	1.22 (0.87 − 1.69)
Years 5+	68 (0.1)	58 (0.1)	4.7	31.5	1.16 (0.82 − 1.64)
**All after year 1**	**397 (0.2)**	**320 (0.2)**	**38.6**	**179.2**	**1.24 (1.07 − 1.44)**
**All years**	**783 (0.3)**	**556 (0.2)**	**114.2**	**334.7**	**1.41 (1.26 − 1.57)**

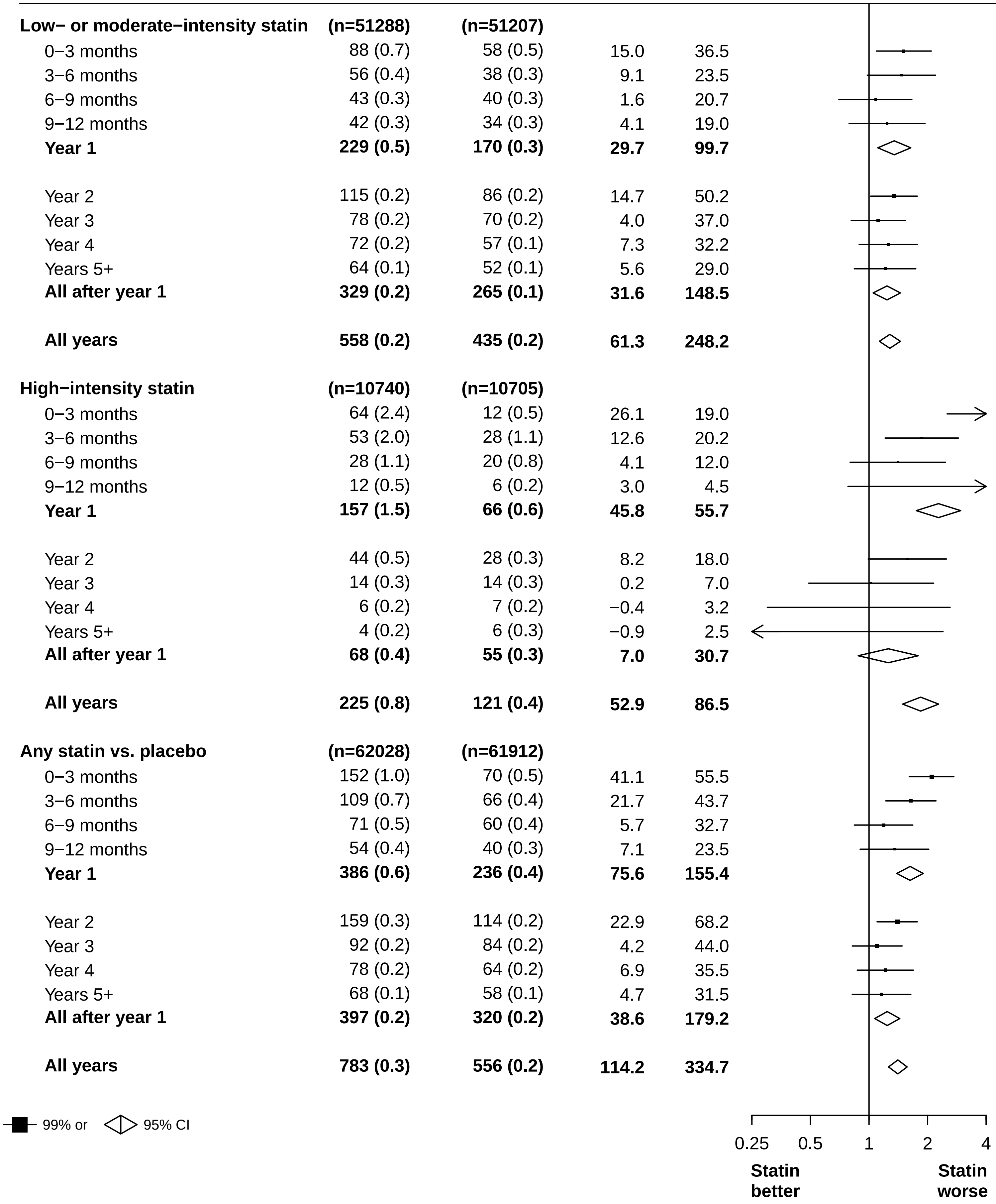

For low− or moderate−intensity statin trials, the trend test for duration (Y1 vs Y1+) was: χ^2_1 =0.4, p=0.51

For high−intensity statin trials, the trend test for duration (Y1 vs Y1+) was: χ^2_1 =7.0, p=0.0082

For any−intensity statin trials, the trend test for duration (Y1 vs Y1+) was: χ^2_1 =6.1, p=0.013

Webfigure 4b: Effect of statin vs placebo on HEPATOBILIARY DISORDERS: OTHER LIVER FUNCTION TEST ABNORMALITY, subdivided by duration of follow−up

Year	Events (% per annum): Statin	Events (% per annum): Placebo	Observed−Expected: O−E	Observed−Expected: Var(O−E)	Rate Ratio (95% CI)
**Low− or moderate−intensity statin**	**(n=51288)**	**(n=51207)**			
0−3 months	47 (0.4)	42 (0.3)	2.5	22.2	1.12 (0.74 − 1.70)
3−6 months	43 (0.3)	34 (0.3)	4.5	19.2	1.26 (0.81 − 1.98)
6−9 months	26 (0.2)	20 (0.2)	3.0	11.5	1.30 (0.73 − 2.31)
9−12 months	36 (0.3)	30 (0.2)	3.0	16.5	1.20 (0.74 − 1.94)
**Year 1**	**152 (0.3)**	**126 (0.2)**	**13.0**	**69.5**	**1.21 (0.95 − 1.53)**
Year 2	101 (0.2)	86 (0.2)	7.5	46.7	1.17 (0.88 − 1.56)
Year 3	110 (0.2)	112 (0.2)	−1.1	55.5	0.98 (0.75 − 1.28)
Year 4	70 (0.2)	76 (0.2)	−3.4	36.5	0.91 (0.66 − 1.26)
Years 5+	45 (<0.1)	44 (<0.1)	−0.1	22.2	1.00 (0.66 − 1.51)
**All after year 1**	**326 (0.2)**	**318 (0.2)**	**3.0**	**161.0**	**1.02 (0.87 − 1.19)**
**All years**	**478 (0.2)**	**444 (0.2)**	**16.0**	**230.5**	**1.07 (0.94 − 1.22)**
**High−intensity statin**	**(n=10740)**	**(n=10705)**			
0−3 months	59 (2.2)	11 (0.4)	24.1	17.5	3.97 (2.49 − 6.35)
3−6 months	29 (1.1)	10 (0.4)	9.6	9.7	2.69 (1.44 − 5.04)
6−9 months	18 (0.7)	5 (0.2)	6.6	5.7	3.13 (1.38 − 7.09)
9−12 months	9 (0.3)	4 (0.2)	2.6	3.2	2.20 (0.74 − 6.53)
**Year 1**	**115 (1.1)**	**30 (0.3)**	**42.9**	**36.2**	**3.27 (2.36 − 4.52)**
Year 2	34 (0.4)	19 (0.2)	7.8	13.2	1.80 (1.05 − 3.08)
Year 3	12 (0.3)	14 (0.3)	−0.8	6.5	0.89 (0.41 − 1.91)
Year 4	4 (0.1)	6 (0.2)	−0.9	2.5	0.69 (0.20 − 2.38)
Years 5+	8 (0.4)	5 (0.2)	1.6	3.2	1.63 (0.55 − 4.85)
**All after year 1**	**58 (0.3)**	**44 (0.2)**	**7.7**	**25.5**	**1.35 (0.92 − 1.99)**
**All years**	**173 (0.6)**	**74 (0.3)**	**50.5**	**61.7**	**2.27 (1.77 − 2.91)**
**Any statin vs. placebo**	**(n=62028)**	**(n=61912)**			
0−3 months	106 (0.7)	53 (0.3)	26.7	39.7	1.96 (1.43 − 2.67)
3−6 months	72 (0.5)	44 (0.3)	14.2	29.0	1.63 (1.13 − 2.35)
6−9 months	44 (0.3)	25 (0.2)	9.6	17.2	1.74 (1.09 − 2.79)
9−12 months	45 (0.3)	34 (0.2)	5.5	19.7	1.32 (0.85 − 2.06)
**Year 1**	**267 (0.4)**	**156 (0.3)**	**55.9**	**105.7**	**1.70 (1.40 − 2.05)**
Year 2	135 (0.2)	105 (0.2)	15.3	60.0	1.29 (1.00 − 1.66)
Year 3	122 (0.2)	126 (0.2)	−1.9	62.0	0.97 (0.76 − 1.24)
Year 4	74 (0.2)	82 (0.2)	−4.3	39.0	0.90 (0.65 − 1.23)
Years 5+	53 (0.1)	49 (0.1)	1.5	25.5	1.06 (0.72 − 1.56)
**All after year 1**	**384 (0.2)**	**362 (0.2)**	**10.6**	**186.5**	**1.06 (0.92 − 1.22)**
**All years**	**651 (0.3)**	**518 (0.2)**	**66.6**	**292.2**	**1.26 (1.12 − 1.41)**

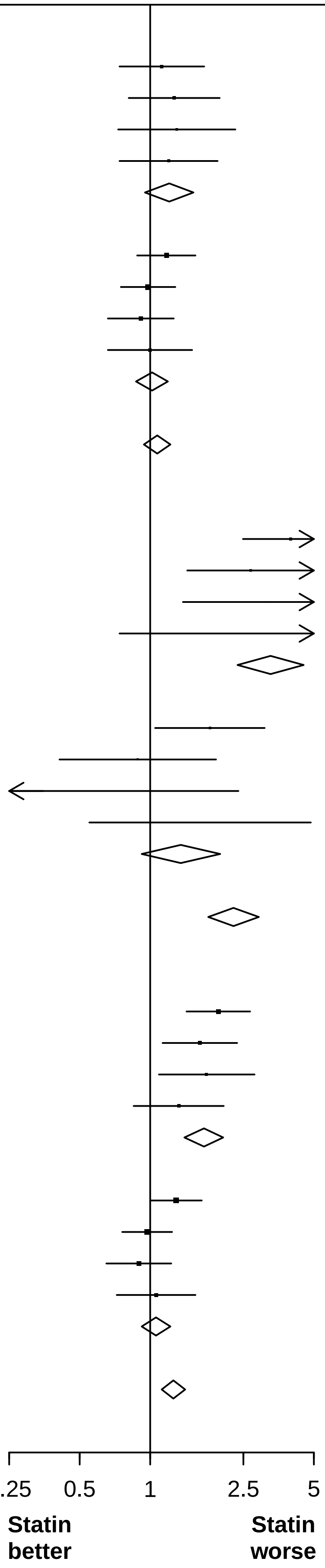

99% or 95% CI

For low− or moderate−intensity statin trials, the trend test for duration (Y1 vs Y1+) was: χ^2_1 =1.4, p=0.24

For high−intensity statin trials, the trend test for duration (Y1 vs Y1+) was: χ^2_1 =11.7, p=0.00063

For any−intensity statin trials, the trend test for duration (Y1 vs Y1+) was: χ^2_1 =15.0, p=0.00011

Webfigure 4c: Effect of statin vs placebo on RENAL AND URINARY DISORDERS: URINARY COMPOSITION ALTERATION, subdivided by duration of follow−up

Year	Events (% per annum) Statin	Events (% per annum) Placebo	Observed−Expected O−E	Observed−Expected Var(O−E)	Rate Ratio (95% CI)
**Low− or moderate−intensity statin**	**(n=51288)**	**(n=51207)**			
0−3 months	28 (0.2)	14 (0.1)	7.0	10.5	1.95 (1.06 − 3.57)
3−6 months	30 (0.2)	25 (0.2)	2.5	13.7	1.20 (0.71 − 2.04)
6−9 months	30 (0.2)	20 (0.2)	5.0	12.5	1.50 (0.86 − 2.60)
9−12 months	25 (0.2)	23 (0.2)	1.0	12.0	1.09 (0.62 − 1.92)
**Year 1**	**113 (0.2)**	**82 (0.2)**	**15.6**	**48.7**	**1.38 (1.04 − 1.82)**
Year 2	107 (0.2)	89 (0.2)	9.2	49.0	1.21 (0.91 − 1.60)
Year 3	111 (0.2)	110 (0.2)	0.8	55.2	1.01 (0.78 − 1.32)
Year 4	66 (0.2)	51 (0.1)	7.3	29.2	1.28 (0.89 − 1.84)
Years 5+	40 (<0.1)	23 (<0.1)	8.2	15.7	1.68 (1.03 − 2.76)
**All after year 1**	**324 (0.2)**	**273 (0.2)**	**25.4**	**149.2**	**1.19 (1.01 − 1.39)**
**All years**	**437 (0.2)**	**355 (0.2)**	**41.0**	**198.0**	**1.23 (1.07 − 1.41)**
**High−intensity statin**	**(n=10740)**	**(n=10705)**			
0−3 months	11 (0.4)	3 (0.1)	4.0	3.5	3.12 (1.10 − 8.91)
3−6 months	20 (0.8)	35 (1.3)	−7.5	13.7	0.58 (0.34 − 0.98)
6−9 months	16 (0.6)	15 (0.6)	0.5	7.7	1.06 (0.53 − 2.15)
9−12 months	14 (0.5)	13 (0.5)	0.5	6.7	1.07 (0.50 − 2.28)
**Year 1**	**61 (0.6)**	**66 (0.6)**	**−2.6**	**31.7**	**0.92 (0.65 − 1.30)**
Year 2	38 (0.4)	35 (0.4)	1.4	18.2	1.08 (0.68 − 1.71)
Year 3	15 (0.3)	11 (0.3)	2.0	6.5	1.37 (0.63 − 2.95)
Year 4	3 (<0.1)	5 (0.2)	−1.0	2.0	0.61 (0.15 − 2.44)
Years 5+	2 (<0.1)	0 (0.0)	1.0	0.5	
**All after year 1**	**58 (0.3)**	**51 (0.3)**	**3.5**	**27.2**	**1.14 (0.78 − 1.66)**
**All years**	**119 (0.4)**	**117 (0.4)**	**0.9**	**59.0**	**1.02 (0.79 − 1.31)**
**Any statin vs. placebo**	**(n=62028)**	**(n=61912)**			
0−3 months	39 (0.3)	17 (0.1)	11.0	14.0	2.19 (1.30 − 3.70)
3−6 months	50 (0.3)	60 (0.4)	−5.0	27.5	0.83 (0.57 − 1.21)
6−9 months	46 (0.3)	35 (0.2)	5.5	20.2	1.31 (0.85 − 2.03)
9−12 months	39 (0.3)	36 (0.2)	1.5	18.7	1.08 (0.69 − 1.70)
**Year 1**	**174 (0.3)**	**148 (0.2)**	**13.0**	**80.5**	**1.18 (0.94 − 1.46)**
Year 2	145 (0.3)	124 (0.2)	10.6	67.2	1.17 (0.92 − 1.49)
Year 3	126 (0.2)	121 (0.2)	2.8	61.7	1.05 (0.82 − 1.34)
Year 4	69 (0.2)	56 (0.1)	6.3	31.2	1.22 (0.86 − 1.74)
Years 5+	42 (<0.1)	23 (<0.1)	9.2	16.2	1.76 (1.09 − 2.87)
**All after year 1**	**382 (0.2)**	**324 (0.2)**	**28.9**	**176.5**	**1.18 (1.02 − 1.37)**
**All years**	**556 (0.2)**	**472 (0.2)**	**41.9**	**256.9**	**1.18 (1.04 − 1.33)**

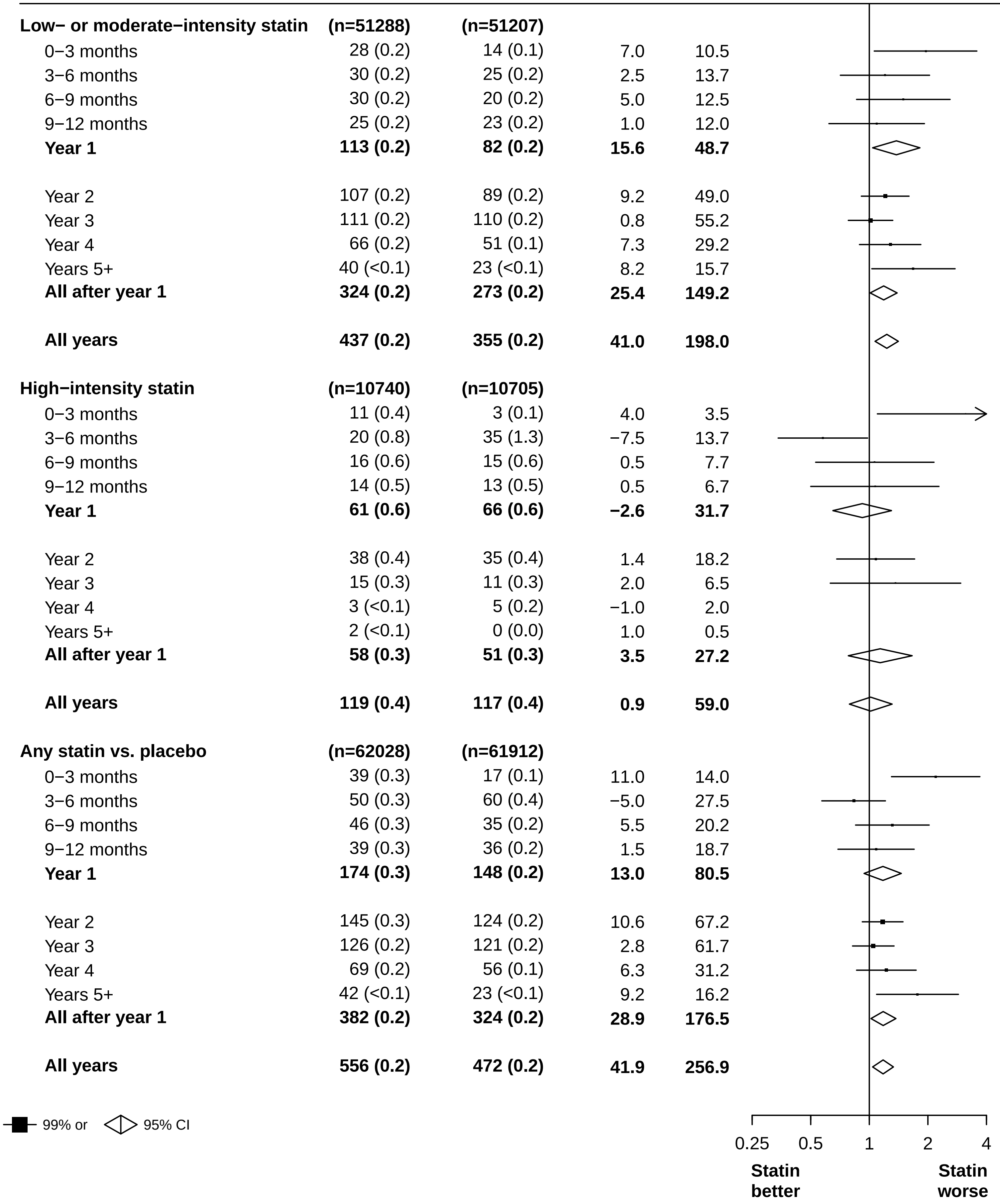

For low− or moderate−intensity statin trials, the trend test for duration (Y1 vs Y1+) was: χ^2_1 =0.8, p=0.36

For high−intensity statin trials, the trend test for duration (Y1 vs Y1+) was: χ^2_1 =0.7, p=0.42

For any−intensity statin trials, the trend test for duration (Y1 vs Y1+) was: χ^2_1 =0.1, p=0.98

Webfigure 4d: Effect of statin vs placebo on GENERAL DISORDERS AND ADMINISTRATION SITE CONDITIONS: OEDEMA, subdivided by duration of follow−up

Year	Events (% per annum) Statin	Events (% per annum) Placebo	Observed−Expected O−E	Observed−Expected Var(O−E)	Rate Ratio (95% CI)
**Low− or moderate−intensity statin**	**(n=51288)**	**(n=51207)**			
0−3 months	975 (7.7)	890 (7.1)	46.6	464.0	1.11 (1.01 − 1.21)
3−6 months	277 (2.2)	246 (2.0)	17.2	130.7	1.14 (0.96 − 1.35)
6−9 months	148 (1.2)	153 (1.2)	−2.0	75.2	0.97 (0.78 − 1.22)
9−12 months	161 (1.3)	183 (1.5)	−10.0	86.0	0.89 (0.72 − 1.10)
**Year 1**	**1561 (3.2)**	**1472 (3.0)**	**51.8**	**755.9**	**1.07 (1.00 − 1.15)**
Year 2	569 (1.2)	547 (1.2)	13.7	278.9	1.05 (0.93 − 1.18)
Year 3	487 (1.1)	467 (1.0)	11.8	238.5	1.05 (0.93 − 1.19)
Year 4	305 (0.8)	282 (0.8)	12.0	146.7	1.09 (0.92 − 1.28)
Years 5+	158 (0.3)	164 (0.4)	−2.9	80.4	0.96 (0.77 − 1.20)
**All after year 1**	**1519 (0.9)**	**1460 (0.8)**	**34.5**	**744.5**	**1.05 (0.97 − 1.13)**
**All years**	**3080 (1.4)**	**2932 (1.3)**	**86.3**	**1500.4**	**1.06 (1.01 − 1.11)**
**High−intensity statin**	**(n=10740)**	**(n=10705)**			
0−3 months	80 (3.0)	58 (2.2)	10.9	34.5	1.37 (0.98 − 1.92)
3−6 months	63 (2.4)	47 (1.8)	8.0	27.5	1.34 (0.92 − 1.95)
6−9 months	31 (1.2)	29 (1.1)	1.0	15.0	1.07 (0.65 − 1.78)
9−12 months	35 (1.4)	36 (1.4)	−0.5	17.7	0.97 (0.61 − 1.55)
**Year 1**	**209 (2.0)**	**170 (1.6)**	**19.5**	**94.7**	**1.23 (1.00 − 1.50)**
Year 2	106 (1.2)	106 (1.2)	0.1	53.0	1.00 (0.77 − 1.31)
Year 3	56 (1.3)	43 (1.0)	6.7	24.7	1.31 (0.89 − 1.95)
Year 4	32 (1.1)	31 (1.0)	0.7	15.7	1.04 (0.64 − 1.71)
Years 5+	12 (0.5)	17 (0.7)	−2.5	7.2	0.71 (0.34 − 1.47)
**All after year 1**	**206 (1.2)**	**197 (1.1)**	**5.0**	**100.7**	**1.05 (0.86 − 1.28)**
**All years**	**415 (1.5)**	**367 (1.3)**	**24.6**	**195.5**	**1.13 (0.99 − 1.30)**
**Any statin vs. placebo**	**(n=62028)**	**(n=61912)**			
0−3 months	1055 (6.9)	948 (6.2)	57.6	498.5	1.12 (1.03 − 1.23)
3−6 months	340 (2.3)	293 (1.9)	25.2	158.2	1.17 (1.00 − 1.37)
6−9 months	179 (1.2)	182 (1.2)	−1.0	90.2	0.99 (0.80 − 1.22)
9−12 months	196 (1.3)	219 (1.5)	−10.5	103.7	0.90 (0.75 − 1.10)
**Year 1**	**1770 (3.0)**	**1642 (2.7)**	**71.3**	**850.7**	**1.09 (1.02 − 1.16)**
Year 2	675 (1.2)	653 (1.2)	13.8	331.9	1.04 (0.94 − 1.16)
Year 3	543 (1.1)	510 (1.0)	18.5	263.2	1.07 (0.95 − 1.21)
Year 4	337 (0.8)	313 (0.8)	12.6	162.5	1.08 (0.93 − 1.26)
Years 5+	170 (0.4)	181 (0.4)	−5.4	87.7	0.94 (0.76 − 1.16)
**All after year 1**	**1725 (0.9)**	**1657 (0.9)**	**39.5**	**845.3**	**1.05 (0.98 − 1.12)**
**All years**	**3495 (1.4)**	**3299 (1.3)**	**110.8**	**1695.9**	**1.07 (1.02 − 1.12)**

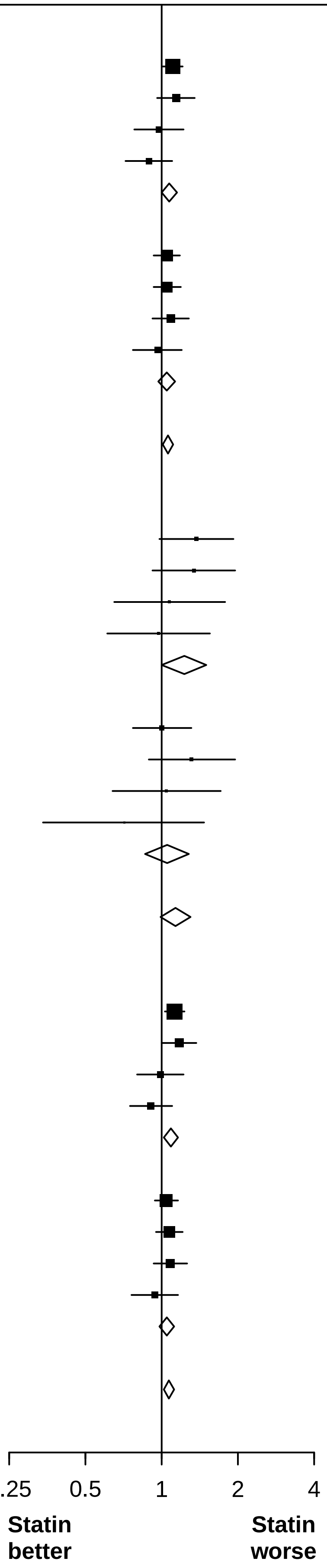

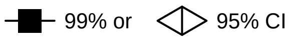

For low− or moderate−intensity statin trials, the trend test for duration (Y1 vs Y1+) was: χ^2_1 =0.2, p=0.67

For high−intensity statin trials, the trend test for duration (Y1 vs Y1+) was: χ^2_1 =1.2, p=0.27

For any−intensity statin trials, the trend test for duration (Y1 vs Y1+) was: χ^2_1 =0.6, p=0.45

Webfigure 5a: Effect of more vs less intensive statin on GASTROINTESTINAL, HEPATOBILIARY, AND METABOLISM AND NUTRITION DISORDERS listed in statin SmPCs, subdivided by category component parts

Endpoint	Events (% per annum)		Observed−Expected		Rate Ratio (95% CI)
	More statin (n=15390)	Less statin (n=15334)	O−E	Var(O−E)	
**Gastrointestinal disorders**					
Abdominal pain	795 (1.1)	792 (1.1)	0.5	396.6	1.00 (0.91 − 1.10)
Constipation	467 (0.7)	465 (0.6)	0.5	232.9	1.00 (0.88 − 1.14)
Diarrhoea	649 (0.9)	568 (0.8)	41.5	304.1	1.15 (1.02 − 1.28)
Dyspepsia	659 (0.9)	634 (0.9)	11.8	323.2	1.04 (0.93 − 1.16)
Eructation and flatulence	137 (0.2)	173 (0.2)	−18.5	77.5	0.79 (0.63 − 0.98)
Nausea and vomiting	450 (0.6)	417 (0.6)	16.7	216.7	1.08 (0.95 − 1.23)
Pancreatitis	62 (<0.1)	80 (0.1)	−9.1	35.5	0.77 (0.56 − 1.08)
**Hepatobiliary disorders**					
Cholestasis and jaundice	43 (<0.1)	27 (<0.1)	8.0	17.5	1.58 (0.99 − 2.52)
Hepatic failure or damage	15 (<0.1)	11 (<0.1)	2.0	6.5	1.36 (0.63 − 2.93)
Hepatitis	11 (<0.1)	15 (<0.1)	−2.0	6.5	0.73 (0.34 − 1.58)
Hepatic steatosis	10 (<0.1)	18 (<0.1)	−4.0	7.0	0.56 (0.27 − 1.18)
Abnormal liver transaminases *	218 (0.3)	102 (0.1)	57.9	80.0	2.06 (1.66 − 2.57)
Other LFT abnormality *†	307 (0.4)	162 (0.2)	73.2	117.2	1.87 (1.56 − 2.24)
**Metabolism and nutrition disorders ****					
Decreased appetite	76 (0.1)	66 (<0.1)	4.9	35.5	1.15 (0.83 − 1.60)
Hyperglycaemia	221 (0.3)	203 (0.3)	9.3	106.0	1.09 (0.90 − 1.32)
Hypoglycaemia	67 (<0.1)	59 (<0.1)	3.9	31.5	1.13 (0.80 − 1.61)
Weight increased	156 (0.2)	137 (0.2)	9.5	73.2	1.14 (0.91 − 1.43)

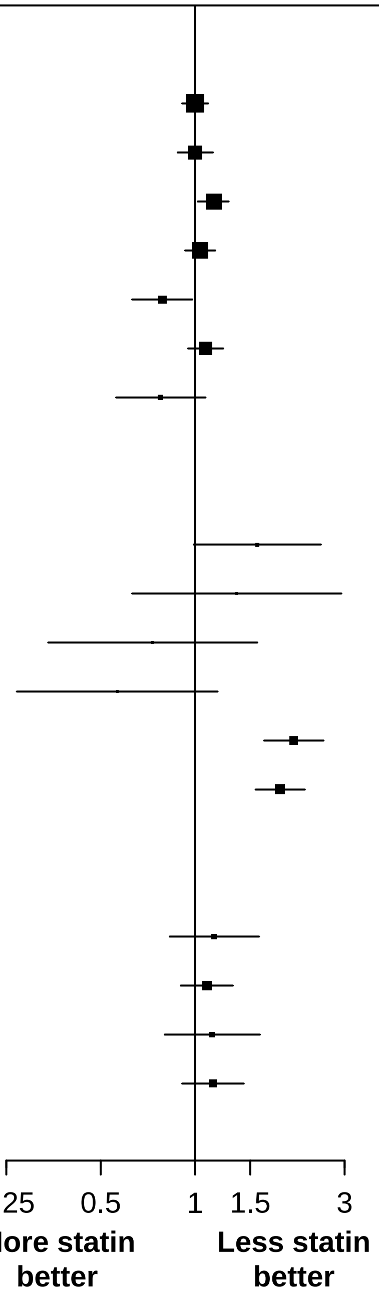

* FDR significant at the 5% level

† LFT=Liver function test

** The outcome diabetes mellitus is also included in statin labels as an potential undesirable effect but has previously been reported separately: <DOI: https://doi.org/10.1016/S2213−8587(24)00040−8>

Webfigure 5b: Effect of more vs less intensive statin on HEPATOBILIARY DISORDERS: ABNORMAL LIVER TRANSAMINASES, subdivided by statin intensity and trial

Trial	Dose	Events (% per annum) More statin (n=15390)	Events (% per annum) Less statin (n=15334)	Observed−Expected O−E	Observed−Expected Var(O−E)	Rate Ratio (95% CI)
**Comparison of moderate−intensity regimens**						
A to Z	S40−80 vs S20	87 (2.3)	54 (1.4)	15.9	35.2	1.57 (1.13 − 2.19)
SEARCH	S80 vs S20	−	−	−	−	
**Subtotal**		**87 (0.2)**	**54 (0.1)**	**15.9**	**35.2**	**1.57 (1.13 − 2.19)**
**Comparison of high− vs moderate−intensity regimens**						
TNT	A80 vs A10	130 (0.5)	47 (0.2)	42.0	44.2	2.58 (1.93 − 3.47)
PROVE−IT	A80 vs P40	1 (<0.1)	1 (<0.1)	0.0	0.5	
**Subtotal**		**131 (0.5)**	**48 (0.2)**	**42.0**	**44.7**	**2.56 (1.91 − 3.43)**
**All trials**		**218 (0.3)**	**102 (0.1)**	**57.9**	**80.0**	**2.06 (1.66 − 2.57)**

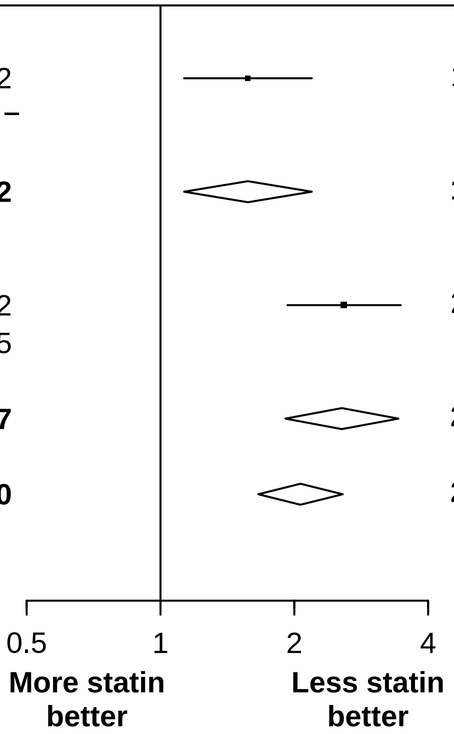

99% or 95% CI

Note: No data is represented by a '−'

RRs are only plotted for outcomes with at least 10 events, though all outcomes contribute to the shown subtotals and totals

Webfigure 5c: Effect of more vs less intensive statin on HEPATOBILIARY DISORDERS: OTHER LIVER FUNCTION TEST ABNORMALITY, subdivided by statin intensity and trial

Trial	Dose	Events (% per annum): More statin (n=15390)	Events (% per annum): Less statin (n=15334)	Observed−Expected: O−E	Observed−Expected: Var(O−E)	Rate Ratio (95% CI)
**Comparison of moderate−intensity regimens**						
A to Z	S40−80 vs S20	12 (0.3)	11 (0.3)	0.4	5.7	1.07 (0.47 − 2.41)
SEARCH	S80 vs S20	14 (<0.1)	13 (<0.1)	0.5	6.7	1.08 (0.51 − 2.29)
**Subtotal**		**26 (<0.1)**	**24 (<0.1)**	**0.9**	**12.5**	**1.07 (0.62 − 1.87)**
**Comparison of high− vs moderate−intensity regimens**						
TNT	A80 vs A10	191 (0.8)	87 (0.4)	52.9	69.5	2.14 (1.69 − 2.71)
PROVE−IT	A80 vs P40	90 (2.2)	51 (1.3)	19.5	35.2	1.74 (1.25 − 2.42)
**Subtotal**		**281 (1.0)**	**138 (0.5)**	**72.3**	**104.7**	**1.99 (1.65 − 2.42)**
**All trials**		**307 (0.4)**	**162 (0.2)**	**73.2**	**117.2**	**1.87 (1.56 − 2.24)**

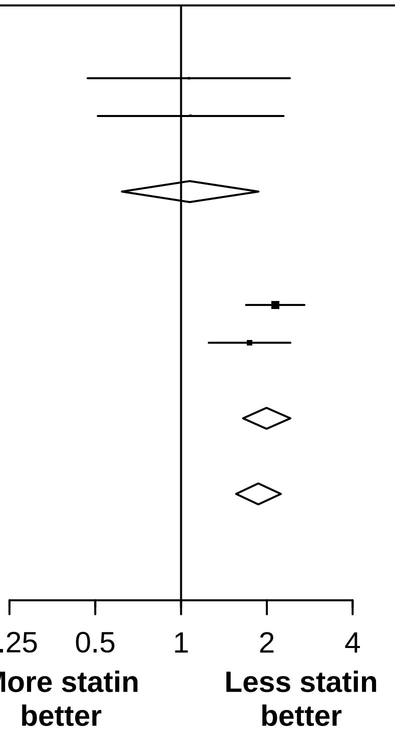

99% or 95% CI

Webfigure 6a: Effect of statin vs placebo on GASTROINTESTINAL, HEPATOBILIARY, AND METABOLISM & NUTRITION DISORDERS listed in statin SmPCs, subdivided by category component parts (includes the post−hoc outcome 'abnormal liver function tests')

Endpoint	Events (% per annum)		Observed−Expected		Rate Ratio (95% CI)
	Statin (n=62028)	Placebo (n=61912)	O−E	Var(O−E)	
**Gastrointestinal disorders**					
Abdominal pain	3443 (1.4)	3367 (1.3)	35.4	1702.1	1.02 (0.97 − 1.07)
Constipation	1865 (0.7)	1810 (0.7)	28.3	918.6	1.03 (0.97 − 1.10)
Diarrhoea	2707 (1.1)	2649 (1.0)	28.3	1338.6	1.02 (0.97 − 1.08)
Dyspepsia	2472 (1.0)	2430 (1.0)	18.5	1225.2	1.02 (0.96 − 1.07)
Eructation and flatulence	722 (0.3)	683 (0.3)	18.3	351.2	1.05 (0.95 − 1.17)
Nausea and vomiting	2111 (0.8)	1999 (0.8)	51.3	1027.2	1.05 (0.99 − 1.12)
Pancreatitis	164 (<0.1)	192 (<0.1)	−14.5	89.0	0.85 (0.69 − 1.05)
**Hepatobiliary disorders**					
Cholestasis and jaundice	144 (<0.1)	118 (<0.1)	12.8	65.5	1.22 (0.95 − 1.55)
Hepatic failure or damage	73 (<0.1)	68 (<0.1)	2.4	35.2	1.07 (0.77 − 1.49)
Hepatitis	48 (<0.1)	45 (<0.1)	1.4	23.2	1.06 (0.71 − 1.59)
Hepatic steatosis	43 (<0.1)	54 (<0.1)	−5.6	24.2	0.79 (0.53 − 1.18)
Abnormal liver function tests * ‡	1360 (0.5)	1020 (0.4)	172.9	594.8	1.34 (1.23 − 1.45)
**Metabolism and nutrition disorders** **					
Decreased appetite	503 (0.2)	486 (0.2)	8.4	247.2	1.03 (0.91 − 1.17)
Hyperglycaemia	766 (0.3)	706 (0.3)	29.7	367.9	1.08 (0.98 − 1.20)
Hypoglycaemia	373 (0.1)	338 (0.1)	16.3	177.7	1.10 (0.95 − 1.27)
Weight increased	578 (0.2)	516 (0.2)	29.5	273.5	1.11 (0.99 − 1.25)

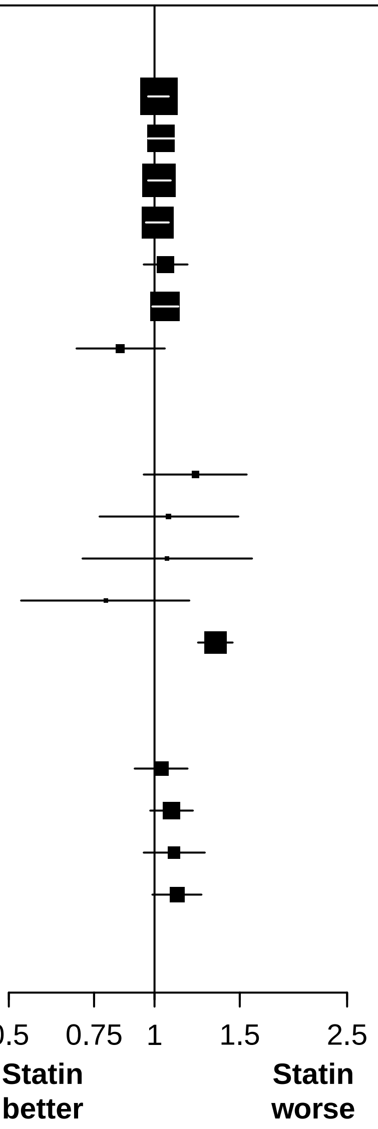

* FDR significant at the 5% level.

‡ Abnormal liver function tests is the composite of the outcomes 'abnormal liver transaminases' or 'other liver function test abnormality'

** The outcome diabetes mellitus is also included in statin labels as an undesirable effect but has previously been reported separately: <DOI: https://doi.org/10.1016/S2213−8587(24)00040−8>

Webfigure 6b: Effect of statin vs placebo on HEPATOBILIARY DISORDERS: ABNORMAL LIVER FUNCTION TESTS ‡, subdivided by statin intensity and trial

Trial	Dose	Events (% per annum) Statin (n=62028)	Events (% per annum) Placebo (n=61912)	Observed−Expected O−E	Observed−Expected Var(O−E)	Rate Ratio (95% CI)
**Low− or moderate−intensity statin**						
**Lovastatin**						
AFCAPS/TexCAPS	20−40mg	157 (1.0)	108 (0.7)	24.5	66.2	1.45 (1.14 − 1.84)
**Subtotal**		**157 (1.0)**	**108 (0.7)**	**24.5**	**66.2**	**1.45 (1.14 − 1.84)**
**Fluvastatin**						
ALERT	40−80mg	28 (0.5)	21 (0.4)	3.6	12.2	1.34 (0.77 − 2.35)
LIPS	80mg	14 (0.4)	10 (0.3)	1.9	6.0	1.37 (0.62 − 3.06)
**Subtotal**		**42 (0.5)**	**31 (0.4)**	**5.5**	**18.2**	**1.35 (0.85 − 2.14)**
**Simvastatin**						
4S	20−40mg	254 (2.4)	203 (1.9)	26.3	114.2	1.26 (1.05 − 1.51)
HPS	40mg	17 (<0.1)	22 (<0.1)	−2.6	9.7	0.77 (0.41 − 1.44)
**Subtotal**		**271 (0.4)**	**225 (0.4)**	**23.7**	**124.0**	**1.21 (1.02 − 1.44)**
**Pravastatin**						
WOSCOPS	40mg	32 (0.2)	31 (0.2)	0.3	15.7	1.02 (0.62 − 1.68)
CARE	40mg	109 (1.1)	122 (1.3)	−7.1	57.7	0.88 (0.68 − 1.15)
LIPID	40mg	63 (0.2)	43 (0.2)	9.8	26.5	1.45 (0.99 − 2.12)
PROSPER	40mg	85 (0.9)	73 (0.8)	6.4	39.5	1.17 (0.86 − 1.61)
**Subtotal**		**289 (0.5)**	**269 (0.4)**	**9.4**	**139.5**	**1.07 (0.91 − 1.26)**
**Atorvastatin**						
ASCOT−LLA	10mg	39 (0.2)	39 (0.2)	−0.1	19.5	1.00 (0.64 − 1.55)
ASPEN	10mg	30 (0.7)	34 (0.8)	−2.5	16.0	0.86 (0.53 − 1.40)
CARDS	10mg	34 (0.6)	39 (0.7)	−2.8	18.2	0.86 (0.54 − 1.36)
4D	20mg	10 (0.6)	9 (0.5)	0.5	4.7	1.11 (0.45 − 2.72)
**Subtotal**		**113 (0.4)**	**121 (0.4)**	**−4.8**	**58.5**	**0.92 (0.71 − 1.19)**
**Rosuvastatin**						
CORONA	10mg	28 (0.5)	16 (0.3)	5.9	11.0	1.72 (0.95 − 3.10)
GISSI−HF	10mg	31 (0.4)	18 (0.2)	6.6	12.2	1.71 (0.98 − 3.00)
AURORA	10mg	25 (0.6)	23 (0.5)	1.0	12.0	1.09 (0.62 − 1.92)
HOPE−3	10mg	18 (<0.1)	16 (<0.1)	1.0	8.5	1.12 (0.57 − 2.19)
**Subtotal**		**102 (0.2)**	**73 (0.1)**	**14.5**	**43.7**	**1.39 (1.04 − 1.87)**
**Any low− or moderate−intensity**		**974 (0.4)**	**827 (0.4)**	**72.9**	**450.1**	**1.18 (1.07 − 1.29)**
**High−intensity statin**						
**Atorvastatin**						
SPARCL	80mg	226 (2.2)	72 (0.7)	80.5	74.4	2.95 (2.35 − 3.70)
**Subtotal**		**226 (2.2)**	**72 (0.7)**	**80.5**	**74.4**	**2.95 (2.35 − 3.70)**
**Rosuvastatin**						
JUPITER	20mg	160 (0.9)	121 (0.7)	19.5	70.2	1.32 (1.05 − 1.67)
**Subtotal**		**160 (0.9)**	**121 (0.7)**	**19.5**	**70.2**	**1.32 (1.05 − 1.67)**
**Any high−intensity**		**386 (1.4)**	**193 (0.7)**	**100.0**	**144.6**	**2.00 (1.70 − 2.35)**
**All trials**		**1360 (0.5)**	**1020 (0.4)**	**172.9**	**594.8**	**1.34 (1.23 − 1.45)**

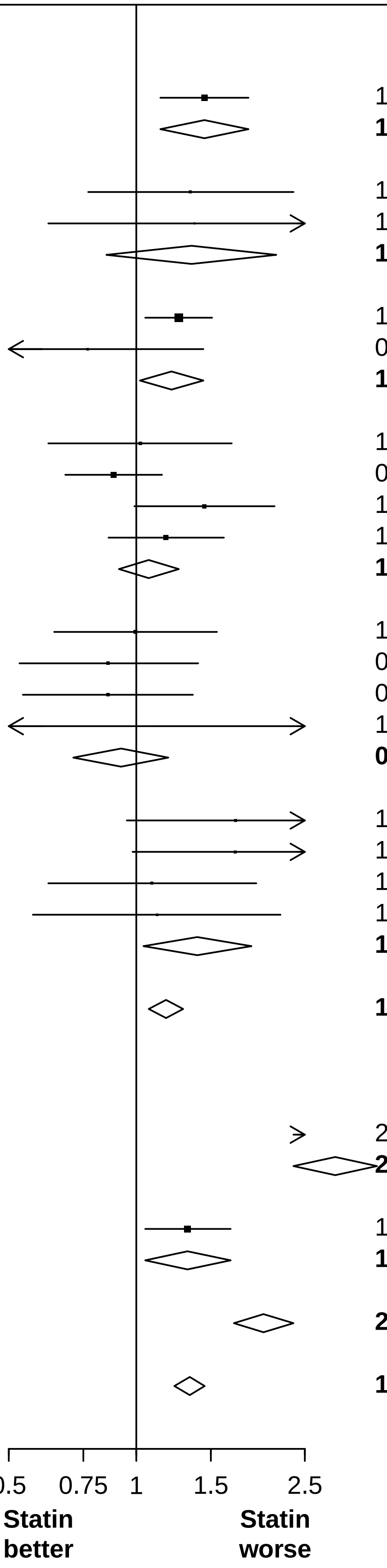

99% or 95% CI

Test for heterogeneity between 6 statins in the low− or moderate−intensity analysis: χ^2_5 =9.3, p=0.096

Test for heterogeneity between 2 statins in the high−intensity trials analysis: χ^2_1 =23.3, p<0.0001

Test for heterogeneity between low− or moderate−intensity and high−intensity trials analysis: χ^2_1 =30.7, p<0.0001

‡ Abnormal liver function tests is the composite of the outcomes 'abnormal liver transaminases' or 'other liver function test abnormality'

Webfigure 6c: Effect of statin vs placebo on HEPATOBILIARY DISORDERS: ABNORMAL LIVER FUNCTION TESTS ‡, subdivided by baseline characteristics

Subgroup	Events (% per annum) Statin (n=62028)	Events (% per annum) Placebo (n=61912)	Observed−Expected O−E	Observed−Expected Var(O−E)	Rate Ratio (95% CI)
**Age, years** (Trend: χ^2_1 =4.2, p=0.040)					
≤65	906 (0.6)	717 (0.5)	100.3	405.5	1.28 (1.16 − 1.41)
>65, ≤75	338 (0.4)	238 (0.3)	47.7	143.9	1.39 (1.18 − 1.64)
>75	116 (0.7)	65 (0.4)	26.1	45.2	1.78 (1.33 − 2.38)
**Sex** (Heterogeneity: χ^2_1 =5.2, p=0.022)					
Female	377 (0.5)	244 (0.4)	69.6	155.0	1.57 (1.34 − 1.83)
Male	983 (0.5)	776 (0.4)	103.4	439.6	1.27 (1.15 − 1.39)
**Race/Ethnicity** (Heterogeneity: χ^2_3 =0.6, p=0.90)					
Asian	20 (0.1)	12 (<0.1)	3.9	8.0	1.64 (0.82 − 3.28)
Black	19 (0.7)	16 (0.6)	1.2	8.7	1.15 (0.59 − 2.23)
Caucasian	631 (0.5)	447 (0.3)	93.9	269.4	1.42 (1.26 − 1.60)
Other	43 (0.3)	33 (0.2)	5.6	19.0	1.35 (0.86 − 2.11)
Missing	647 (0.7)	512 (0.6)	68.3	289.7	1.27 (1.13 − 1.42)
**History of vascular disease** (Heterogeneity: χ^2_1 =3.4, p=0.067)					
No	535 (0.4)	439 (0.3)	48.5	243.3	1.22 (1.08 − 1.38)
Yes	825 (0.6)	581 (0.4)	123.7	351.3	1.42 (1.28 − 1.58)
**Body mass index, kg/m^2** (Trend: χ^2_1 =4.5, p=0.034)					
≤25	395 (0.5)	292 (0.4)	54.6	171.7	1.37 (1.18 − 1.60)
>25, ≤30	541 (0.5)	429 (0.4)	54.2	242.3	1.25 (1.10 − 1.42)
>30	268 (0.5)	247 (0.4)	8.7	128.6	1.07 (0.90 − 1.27)
**Estimated GFR, mL/min/1.73m^2** (Trend: χ^2_1 =0.1, p=0.78)					
On dialysis	35 (0.6)	32 (0.5)	1.5	16.7	1.09 (0.68 − 1.76)
≤60	354 (0.6)	241 (0.4)	58.5	148.7	1.48 (1.26 − 1.74)
>60, ≤90	580 (0.4)	459 (0.3)	60.4	259.6	1.26 (1.12 − 1.43)
>90	119 (0.4)	71 (0.2)	24.4	47.4	1.67 (1.26 − 2.22)
**LDL−C, mmol/L** (Trend: χ^2_1 =0.2, p=0.65)					
≤3.0	319 (0.5)	222 (0.3)	49.3	135.2	1.44 (1.22 − 1.70)
>3.0, ≤4.0	545 (0.5)	423 (0.4)	61.5	241.9	1.29 (1.14 − 1.46)
>4.0	484 (0.6)	362 (0.5)	63.5	211.3	1.35 (1.18 − 1.55)
**Total**	**1360 (0.5)**	**1020 (0.4)**	**172.9**	**594.8**	**1.34 (1.23 − 1.45)**

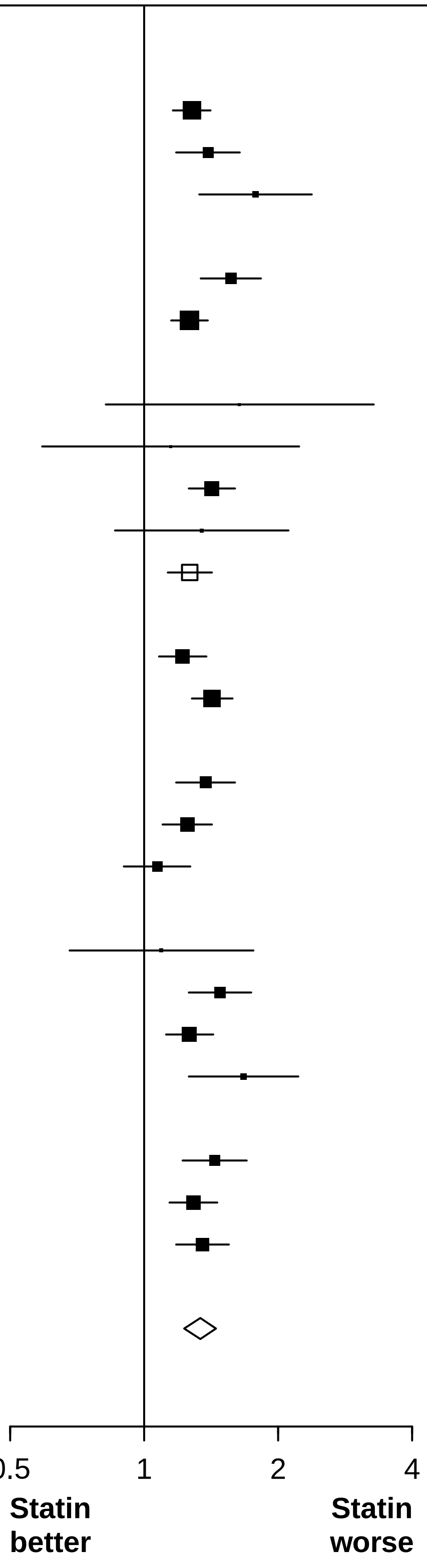

‡ Abnormal liver function tests is the composite of the outcomes 'abnormal liver transaminases' or 'other liver function test abnormality'

Webfigure 6d: Effect of statin vs placebo on HEPATOBILIARY DISORDERS: ABNORMAL LIVER FUNCTION TESTS ‡, subdivided by duration of follow−up

Year	Events (% per annum) Statin	Events (% per annum) Placebo	Observed−Expected O−E	Observed−Expected Var(O−E)	Rate Ratio (95% CI)
**Low− or moderate−intensity statin**	**(n=51288)**	**(n=51207)**			
0−3 months	131 (1.0)	97 (0.8)	17.0	57.0	1.35 (1.04 − 1.75)
3−6 months	93 (0.7)	69 (0.5)	12.1	40.5	1.35 (0.99 − 1.83)
6−9 months	67 (0.5)	57 (0.5)	5.1	31.0	1.18 (0.83 − 1.68)
9−12 months	73 (0.6)	61 (0.5)	6.1	33.5	1.20 (0.85 − 1.68)
**Year 1**	**364 (0.7)**	**284 (0.6)**	**40.3**	**161.9**	**1.28 (1.10 − 1.50)**
Year 2	204 (0.4)	161 (0.3)	21.8	91.2	1.27 (1.03 − 1.56)
Year 3	175 (0.4)	171 (0.4)	2.0	86.5	1.02 (0.83 − 1.26)
Year 4	129 (0.3)	123 (0.3)	2.6	63.0	1.04 (0.81 − 1.33)
Years 5+	102 (0.2)	88 (0.2)	6.1	47.5	1.14 (0.86 − 1.51)
**All after year 1**	**610 (0.3)**	**543 (0.3)**	**32.5**	**288.2**	**1.12 (1.00 − 1.26)**
**All years**	**974 (0.4)**	**827 (0.4)**	**72.9**	**450.1**	**1.18 (1.07 − 1.29)**
**High−intensity statin**	**(n=10740)**	**(n=10705)**			
0−3 months	123 (4.6)	23 (0.9)	50.5	36.5	4.00 (2.89 − 5.53)
3−6 months	81 (3.1)	38 (1.4)	22.0	29.7	2.10 (1.46 − 3.00)
6−9 months	43 (1.7)	24 (0.9)	9.8	16.7	1.79 (1.11 − 2.89)
9−12 months	21 (0.8)	10 (0.4)	5.7	7.7	2.08 (1.03 − 4.22)
**Year 1**	**268 (2.6)**	**95 (0.9)**	**88.0**	**90.7**	**2.64 (2.15 − 3.24)**
Year 2	74 (0.9)	47 (0.5)	14.4	30.2	1.61 (1.13 − 2.30)
Year 3	24 (0.6)	27 (0.6)	−0.9	12.7	0.93 (0.54 − 1.62)
Year 4	9 (0.3)	13 (0.4)	−1.7	5.5	0.73 (0.32 − 1.68)
Years 5+	11 (0.5)	11 (0.5)	0.3	5.5	1.06 (0.46 − 2.45)
**All after year 1**	**118 (0.7)**	**98 (0.5)**	**12.0**	**54.0**	**1.25 (0.96 − 1.63)**
**All years**	**386 (1.4)**	**193 (0.7)**	**100.0**	**144.6**	**2.00 (1.70 − 2.35)**
**Any statin vs. placebo**	**(n=62028)**	**(n=61912)**			
0−3 months	254 (1.6)	120 (0.8)	67.5	93.4	2.06 (1.68 − 2.52)
3−6 months	174 (1.1)	107 (0.7)	34.1	70.2	1.63 (1.29 − 2.05)
6−9 months	110 (0.7)	81 (0.5)	14.9	47.7	1.37 (1.03 − 1.81)
9−12 months	94 (0.6)	71 (0.5)	11.8	41.2	1.33 (0.98 − 1.80)
**Year 1**	**632 (1.0)**	**379 (0.6)**	**128.3**	**252.6**	**1.66 (1.47 − 1.88)**
Year 2	278 (0.5)	208 (0.4)	36.1	121.5	1.35 (1.13 − 1.61)
Year 3	199 (0.4)	198 (0.4)	1.1	99.2	1.01 (0.83 − 1.23)
Year 4	138 (0.3)	136 (0.3)	0.9	68.5	1.01 (0.80 − 1.28)
Years 5+	113 (0.2)	99 (0.2)	6.5	53.0	1.13 (0.86 − 1.48)
**All after year 1**	**728 (0.4)**	**641 (0.3)**	**44.6**	**342.2**	**1.14 (1.02 − 1.27)**
**All years**	**1360 (0.5)**	**1020 (0.4)**	**172.9**	**594.8**	**1.34 (1.23 − 1.45)**

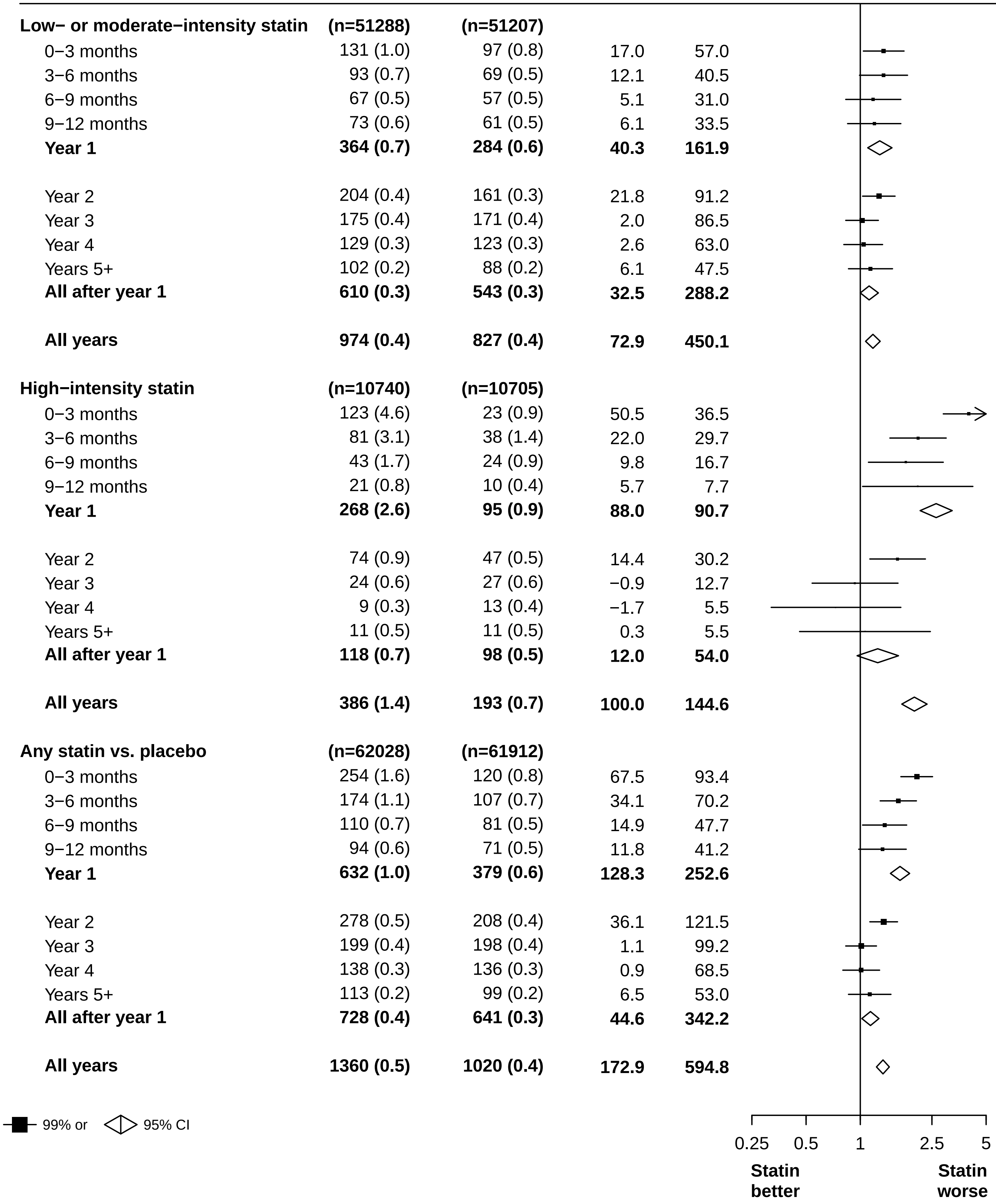

For low− or moderate−intensity statin trials, the trend test for duration (Y1 vs Y1+) was: χ^2_1 =1.9, p=0.17

For high−intensity statin trials, the trend test for duration (Y1 vs Y1+) was: χ^2_1 =18.9, p<0.0001

For any−intensity statin trials, the trend test for duration (Y1 vs Y1+) was: χ^2_1 =20.7, p<0.0001

‡ Abnormal liver function tests is the composite of the outcomes 'abnormal liver transaminases' or 'other liver function test abnormality'

Webfigure 7a: Effect of more vs less intensive statin on GASTROINTESTINAL, HEPATOBILIARY, AND METABOLISM & NUTRITION DISORDERS listed in statin SmPCs, subdivided by category component parts (includes the post−hoc outcome 'abnormal liver function tests')

Endpoint	Events (% per annum) More statin (n=15390)	Events (% per annum) Less statin (n=15334)	Observed−Expected O−E	Observed−Expected Var(O−E)	Rate Ratio (95% CI)
**Gastrointestinal disorders**					
Abdominal pain	795 (1.1)	792 (1.1)	0.5	396.6	1.00 (0.91 − 1.10)
Constipation	467 (0.7)	465 (0.6)	0.5	232.9	1.00 (0.88 − 1.14)
Diarrhoea	649 (0.9)	568 (0.8)	41.5	304.1	1.15 (1.02 − 1.28)
Dyspepsia	659 (0.9)	634 (0.9)	11.8	323.2	1.04 (0.93 − 1.16)
Eructation and flatulence	137 (0.2)	173 (0.2)	−18.5	77.5	0.79 (0.63 − 0.98)
Nausea and vomiting	450 (0.6)	417 (0.6)	16.7	216.7	1.08 (0.95 − 1.23)
Pancreatitis	62 (<0.1)	80 (0.1)	−9.1	35.5	0.77 (0.56 − 1.08)
**Hepatobiliary disorders**					
Cholestasis and jaundice	43 (<0.1)	27 (<0.1)	8.0	17.5	1.58 (0.99 − 2.52)
Hepatic failure or damage	15 (<0.1)	11 (<0.1)	2.0	6.5	1.36 (0.63 − 2.93)
Hepatitis	11 (<0.1)	15 (<0.1)	−2.0	6.5	0.73 (0.34 − 1.58)
Hepatic steatosis	10 (<0.1)	18 (<0.1)	−4.0	7.0	0.56 (0.27 − 1.18)
Abnormal liver function tests * ‡	500 (0.7)	244 (0.3)	129.9	185.9	2.01 (1.74 − 2.32)
**Metabolism and nutrition disorders ** **					
Decreased appetite	76 (0.1)	66 (<0.1)	4.9	35.5	1.15 (0.83 − 1.60)
Hyperglycaemia	221 (0.3)	203 (0.3)	9.3	106.0	1.09 (0.90 − 1.32)
Hypoglycaemia	67 (<0.1)	59 (<0.1)	3.9	31.5	1.13 (0.80 − 1.61)
Weight increased	156 (0.2)	137 (0.2)	9.5	73.2	1.14 (0.91 − 1.43)

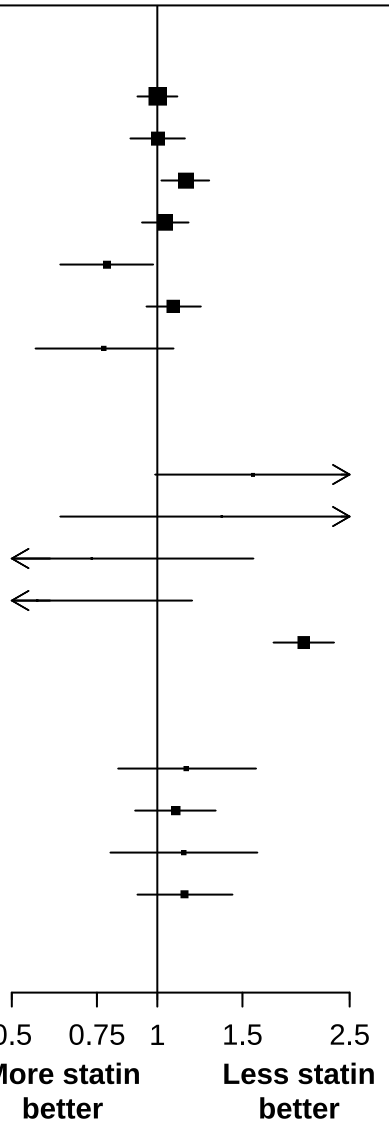

* FDR significant at the 5% level.

‡ Abnormal liver function tests is the composite of the outcomes 'abnormal liver transaminases' or 'other liver function test abnormality'

** The outcome diabetes mellitus is also included in statin labels as an undesirable effect but has previously been reported separately: <DOI: https://doi.org/10.1016/S2213−8587(24)00040−8>

Webfigure 7b: Effect of more vs less intensive statin on HEPATOBILIARY DISORDERS: ABNORMAL LIVER FUNCTION TESTS ‡, subdivided by statin intensity and trial

Trial	Dose	Events (% per annum) More statin (n=15390)	Events (% per annum) Less statin (n=15334)	Observed−Expected O−E	Observed−Expected Var(O−E)	Rate Ratio (95% CI)
**Comparison of moderate−intensity regimens**						
A to Z	S40−80 vs S20	91 (2.4)	55 (1.4)	17.4	36.5	1.61 (1.17 − 2.23)
SEARCH	S80 vs S20	14 (<0.1)	13 (<0.1)	0.5	6.7	1.08 (0.51 − 2.29)
**Subtotal**		**105 (0.2)**	**68 (0.2)**	**17.9**	**43.2**	**1.51 (1.12 − 2.04)**
**Comparison of high− vs moderate−intensity regimens**						
TNT	A80 vs A10	305 (1.3)	125 (0.5)	92.5	107.5	2.36 (1.96 − 2.86)
PROVE−IT	A80 vs P40	90 (2.2)	51 (1.3)	19.5	35.2	1.74 (1.25 − 2.42)
**Subtotal**		**395 (1.4)**	**176 (0.6)**	**111.9**	**142.7**	**2.19 (1.86 − 2.58)**
**All trials**		**500 (0.7)**	**244 (0.3)**	**129.9**	**185.9**	**2.01 (1.74 − 2.32)**

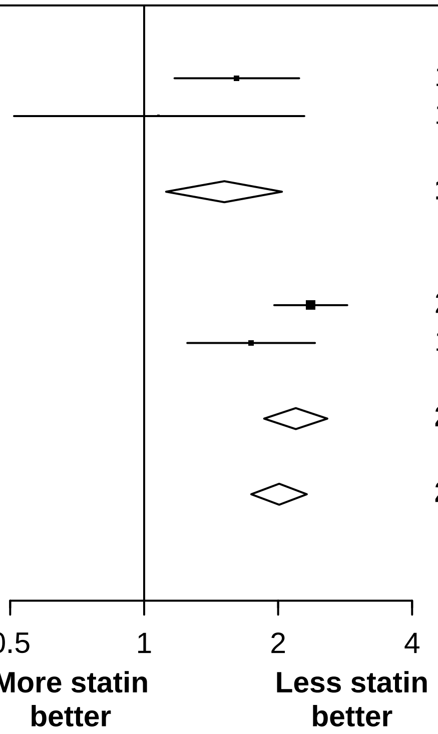

■ 99% or ◇ 95% CI

RRs are only plotted for outcomes with at least 10 events, though all outcomes contribute to the shown subtotals and totals

‡ Abnormal liver function tests is the composite of the outcomes 'abnormal liver transaminases' or 'other liver function test abnormality'

Webfigure 7c: Effect of more vs less intensive statin on HEPATOBILIARY DISORDERS: ABNORMAL LIVER FUNCTION TESTS ‡, subdivided by baseline characteristics

Subgroup	Events (% per annum): More statin (n=15390)	Events (% per annum): Less statin (n=15334)	Observed−Expected: O−E	Observed−Expected: Var(O−E)	Rate Ratio (95% CI)
**Age, years** (Trend: χ^2_1 =6.7, p=0.0097)					
≤65	308 (0.7)	175 (0.4)	67.4	120.7	1.75 (1.46 − 2.09)
>65, ≤75	164 (0.7)	61 (0.2)	52.1	56.2	2.53 (1.95 − 3.28)
>75	27 (0.7)	8 (0.2)	9.8	8.7	3.07 (1.58 − 5.95)
**Sex** (Heterogeneity: χ^2_1 =4.8, p=0.028)					
Female	138 (1.1)	50 (0.4)	45.9	46.9	2.66 (2.00 − 3.54)
Male	362 (0.6)	194 (0.3)	84.5	139.0	1.84 (1.56 − 2.17)
**Race/Ethnicity** (Heterogeneity: χ^2_3 =1.8, p=0.62)					
Asian	13 (1.2)	8 (0.8)	2.0	5.2	1.49 (0.63 − 3.52)
Black	13 (1.4)	10 (0.9)	2.4	5.7	1.52 (0.67 − 3.46)
Caucasian	463 (0.7)	219 (0.3)	123.4	170.4	2.06 (1.78 − 2.40)
Other	11 (1.1)	7 (0.8)	1.3	4.4	1.33 (0.52 − 3.36)
**History of vascular disease**					
No	−	−			
Yes	500 (0.7)	244 (0.3)	129.9	185.9	2.01 (1.74 − 2.32)
**Body mass index, kg/m²** (Trend: χ^2_1 =3.0, p=0.082)					
≤25	117 (0.7)	59 (0.3)	28.7	44.0	1.92 (1.43 − 2.58)
>25, ≤30	180 (0.6)	103 (0.3)	40.6	70.7	1.78 (1.41 − 2.24)
>30	183 (0.8)	65 (0.3)	59.5	62.0	2.61 (2.04 − 3.35)
**Estimated GFR, mL/min/1.73m²** (Trend: χ^2_1 =3.2, p=0.074)					
On dialysis	−	−			
≤60	145 (0.9)	60 (0.4)	42.1	51.2	2.27 (1.73 − 2.99)
>60, ≤90	294 (0.7)	143 (0.3)	78.6	109.2	2.05 (1.70 − 2.48)
>90	46 (0.4)	32 (0.2)	5.4	19.4	1.32 (0.84 − 2.06)
**LDL−C, mmol/L** (Trend: χ^2_1 =0.3, p=0.62)					
≤3.0	416 (0.7)	198 (0.3)	109.8	153.4	2.05 (1.75 − 2.40)
>3.0, ≤4.0	72 (0.6)	37 (0.3)	18.2	27.2	1.95 (1.34 − 2.84)
>4.0	5 (0.6)	3 (0.3)	0.5	2.0	1.29 (0.32 − 5.20)
**Total**	**500 (0.7)**	**244 (0.3)**	**129.9**	**185.9**	**2.01 (1.74 − 2.32)**

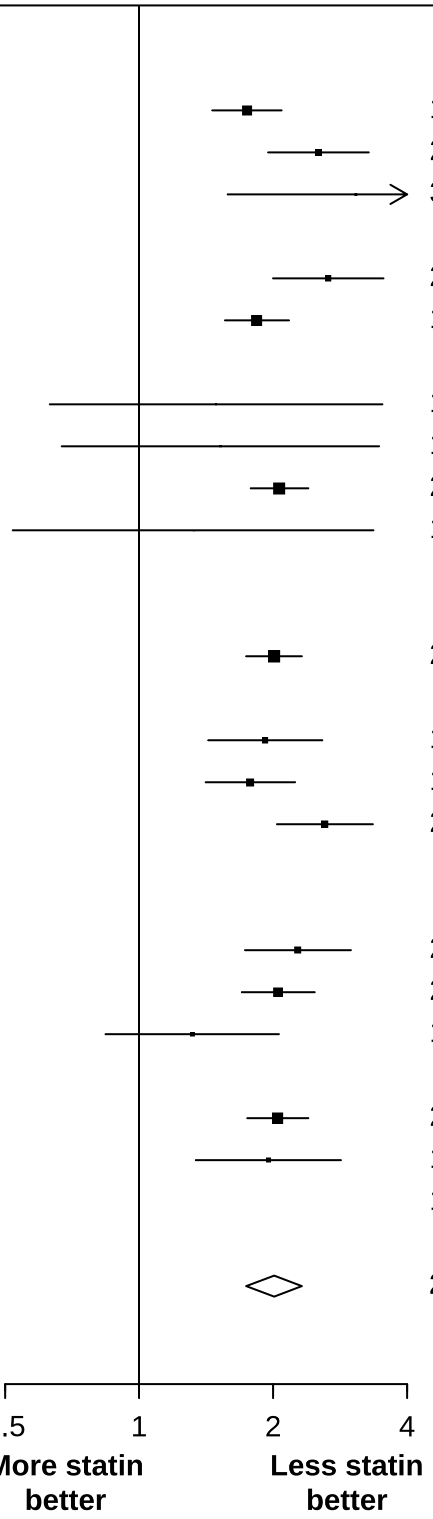

‡ Abnormal liver function tests is the composite of the outcomes 'abnormal liver transaminases' or 'other liver function test abnormality'

Webfigure 7d: Effect of more vs less intensive statin on HEPATOBILIARY DISORDERS: ABNORMAL LIVER FUNCTION TESTS ‡, subdivided by duration of follow−up

Year	Events (% per annum) More statin (n=15390)	Events (% per annum) Less statin (n=15334)	Observed−Expected O−E	Observed−Expected Var(O−E)	Rate Ratio (95% CI)
0−3 months	133 (3.5)	65 (1.7)	33.6	49.5	1.97 (1.49 − 2.61)
3−6 months	95 (2.5)	36 (1.0)	29.6	32.7	2.47 (1.75 − 3.48)
6−9 months	30 (0.8)	13 (0.3)	8.5	10.7	2.20 (1.21 − 4.01)
9−12 months	37 (1.0)	13 (0.4)	12.2	12.5	2.65 (1.52 − 4.61)
**Year 1**	**295 (2.0)**	**127 (0.8)**	**83.9**	**105.4**	**2.22 (1.83 − 2.68)**
Year 2	81 (0.6)	38 (0.3)	21.9	29.7	2.09 (1.46 − 2.99)
Year 3	35 (0.3)	25 (0.2)	5.4	15.0	1.44 (0.87 − 2.38)
Year 4	44 (0.4)	17 (0.2)	14.0	15.2	2.50 (1.51 − 4.13)
Years 5+	45 (0.2)	37 (0.2)	4.7	20.5	1.26 (0.81 − 1.94)
**All years**	**500 (0.7)**	**244 (0.3)**	**129.9**	**185.9**	**2.01 (1.74 − 2.32)**

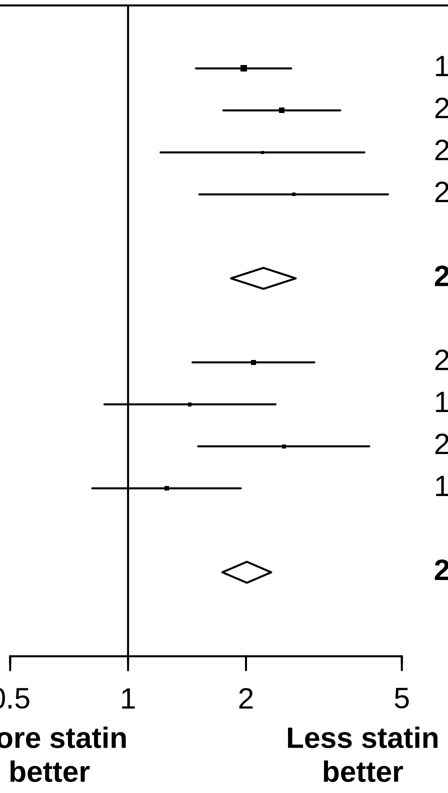

99% or ◇ 95% CI

The trend test for duration was: χ^2_1 =3.9, p=0.049

For each risk period, percentages shown are of those alive and still at risk of a first report of the outcome at the start of the risk period.

‡ Abnormal liver function tests is the composite of the outcomes 'abnormal liver transaminases' or 'other liver function test abnormality'

Webfigure 8a: Effect of statin vs placebo on HEPATOBILIARY DISORDERS: OTHER LIVER FUNCTION TEST ABNORMALITY, subdivided by component parts

Endpoint	Events (% per annum)		Observed−Expected		Rate Ratio (95% CI)
	Statin (n=62028)	Placebo (n=61912)	O−E	Var(O−E)	
Blood ALP* increased	77 (<0.1)	66 (<0.1)	5.4	35.7	1.16 (0.84 − 1.61)
Blood ALP* abnormal	7 (<0.1)	5 (<0.1)	1.0	3.0	1.38 (0.45 − 4.29)
**Subtotal**	**83 (<0.1)**	**70 (<0.1)**	**6.4**	**38.2**	**1.18 (0.86 − 1.62)**
GGT** increased	139 (<0.1)	114 (<0.1)	12.3	63.2	1.21 (0.95 − 1.55)
GGT** abnormal	−	−	−	−	
**Subtotal**	**139 (<0.1)**	**114 (<0.1)**	**12.3**	**63.2**	**1.21 (0.95 − 1.55)**
LFT*** abnormal	114 (<0.1)	107 (<0.1)	3.3	55.2	1.06 (0.81 − 1.38)
LFT*** increased	40 (<0.1)	54 (<0.1)	−7.2	23.5	0.74 (0.49 − 1.11)
Hepatic function abnormal	130 (<0.1)	107 (<0.1)	11.4	59.2	1.21 (0.94 − 1.56)
Hepatic enzyme abnormal	3 (<0.1)	2 (<0.1)	0.5	1.2	1.47 (0.26 − 8.50)
Hepatic enzyme increased	203 (<0.1)	109 (<0.1)	47.3	78.0	1.83 (1.47 − 2.29)
**Subtotal**	**469 (0.2)**	**367 (0.1)**	**51.2**	**209.0**	**1.28 (1.12 − 1.46)**
**Other LFT*** abnormality**	**651 (0.3)**	**518 (0.2)**	**66.6**	**292.2**	**1.26 (1.12 − 1.41)**

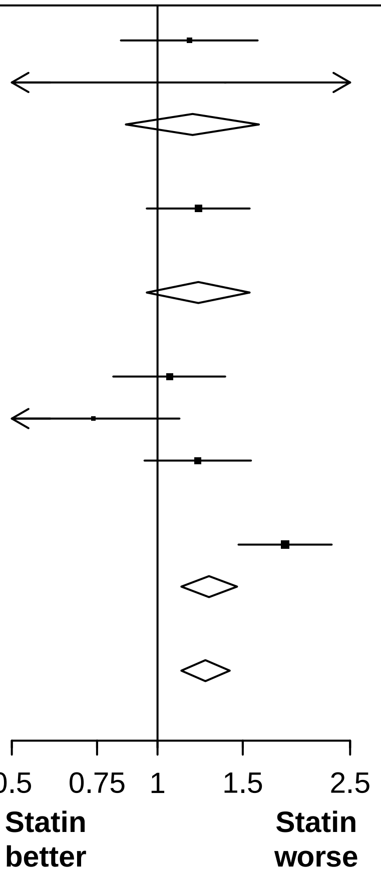

A patient may contribute to more than one row, but only once in each row.

* ALP=Alkaline phosphatase

** GGT=Gamma−glutamyltransferase

*** LFT=Liver function test

Webfigure 8b Effect of more vs less intensive statin on HEPATOBILIARY DISORDERS: OTHER LIVER FUNCTION TEST ABNORMALITY, subdivided by component parts

Endpoint	Events (% per annum) More statin (n=15390)	Events (% per annum) Less statin (n=15334)	Observed−Expected O−E	Observed−Expected Var(O−E)	Rate Ratio (95% CI)
Blood ALP* increased	41 (<0.1)	23 (<0.1)	8.9	16.0	1.75 (1.07 − 2.85)
Blood ALP* abnormal	0 (0.0)	2 (<0.1)	−1.0	0.5	
**Subtotal**	**41 (<0.1)**	**25 (<0.1)**	**7.9**	**16.5**	**1.62 (1.00 − 2.62)**
GGT** increased	16 (<0.1)	15 (<0.1)	0.5	7.7	1.06 (0.52 − 2.14)
GGT** abnormal	0 (0.0)	1 (<0.1)	−0.5	0.2	
**Subtotal**	**16 (<0.1)**	**16 (<0.1)**	**0.0**	**8.0**	**0.99 (0.50 − 1.99)**
LFT*** abnormal	140 (0.2)	73 (0.1)	33.5	53.2	1.88 (1.44 − 2.46)
LFT*** increased	2 (<0.1)	2 (<0.1)	0.0	1.0	0.99 (0.14 − 7.05)
Hepatic function abnormal	10 (<0.1)	6 (<0.1)	2.0	4.0	1.64 (0.62 − 4.37)
Hepatic enzyme abnormal	6 (<0.1)	1 (<0.1)	2.5	1.7	4.18 (0.95 − 18.38)
Hepatic enzyme increased	129 (0.2)	58 (<0.1)	35.6	46.7	2.14 (1.61 − 2.85)
**Subtotal**	**258 (0.4)**	**127 (0.2)**	**66.1**	**96.2**	**1.99 (1.63 − 2.43)**
**Other LFT*** abnormality**	**307 (0.4)**	**162 (0.2)**	**73.2**	**117.2**	**1.87 (1.56 − 2.24)**

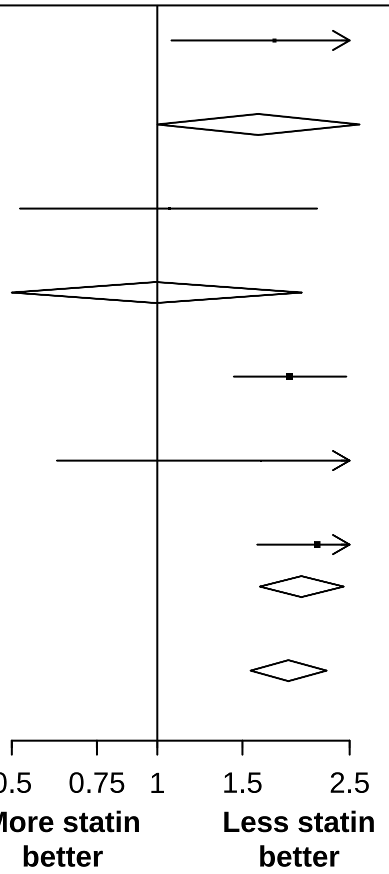

A patient may contribute to more than one row, but only once in each row.

* ALP=Alkaline phosphatase

** GGT=Gamma−glutamyltransferase

*** LFT=Liver function test

Webfigure 9a: Effect of statin vs placebo on RENAL AND URINARY DISORDERS: URINARY COMPOSITION ALTERATION, subdivided by component parts

Endpoint	Events (% per annum) Statin (n=62028)	Events (% per annum) Placebo (n=61912)	Observed−Expected O−E	Observed−Expected Var(O−E)	Rate Ratio (95% CI)
Proteinuria	261 (<0.1)	221 (<0.1)	19.7	120.5	1.18 (0.99 − 1.41)
Albuminuria	4 (<0.1)	11 (<0.1)	−3.6	3.7	0.39 (0.14 − 1.07)
Microalbuminuria	53 (<0.1)	34 (<0.1)	9.1	21.7	1.52 (1.00 − 2.32)
**Subtotal**	**317 (0.1)**	**262 (0.1)**	**26.8**	**144.7**	**1.20 (1.02 − 1.42)**
WBC * urine positive	41 (<0.1)	43 (<0.1)	−1.2	21.0	0.95 (0.62 − 1.45)
RBC ** urine	8 (<0.1)	9 (<0.1)	−0.5	4.2	0.89 (0.34 − 2.29)
RBC ** urine positive	196 (<0.1)	169 (<0.1)	14.0	91.2	1.17 (0.95 − 1.43)
**Subtotal**	**242 (<0.1)**	**216 (<0.1)**	**13.4**	**114.5**	**1.12 (0.94 − 1.35)**
Urine abnormality	17 (<0.1)	14 (<0.1)	1.5	7.7	1.21 (0.60 − 2.45)
**Urinary Composition Alteration**	**556 (0.2)**	**472 (0.2)**	**41.9**	**256.9**	**1.18 (1.04 − 1.33)**

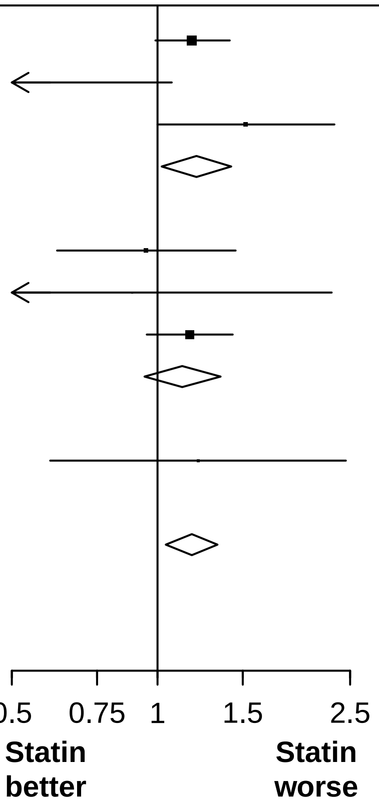

A patient may contribute to more than one row, but only once in each row.

* WBC=White blood cell count

** RBC=Red blood cell count

Webfigure 9b: Effect of more vs less intensive statin on RENAL AND URINARY DISORDERS: URINARY COMPOSITION ALTERATION, subdivided by component parts

Endpoint	Events (% per annum)		Observed−Expected		Rate Ratio (95% CI)
	More statin (n=15390)	Less statin (n=15334)	O−E	Var(O−E)	
Proteinuria	60 (<0.1)	60 (<0.1)	0.0	30.0	1.00 (0.70 − 1.43)
Albuminuria	2 (<0.1)	0 (0.0)	1.0	0.5	
Microalbuminuria	7 (<0.1)	6 (<0.1)	0.5	3.2	1.17 (0.39 − 3.46)
**Subtotal**	**68 (<0.1)**	**66 (<0.1)**	**1.0**	**33.5**	**1.03 (0.73 − 1.44)**
WBC * urine positive	5 (<0.1)	8 (<0.1)	−1.5	3.2	0.63 (0.21 − 1.87)
RBC ** urine	−	−	−	−	
RBC ** urine positive	1 (<0.1)	1 (<0.1)	0.0	0.5	
**Subtotal**	**6 (<0.1)**	**9 (<0.1)**	**−1.5**	**3.7**	**0.67 (0.24 − 1.84)**
Urine abnormality	7 (<0.1)	5 (<0.1)	1.0	3.0	1.40 (0.45 − 4.34)
**Urinary Composition Alteration**	**81 (0.1)**	**78 (0.1)**	**1.5**	**39.7**	**1.04 (0.76 − 1.42)**

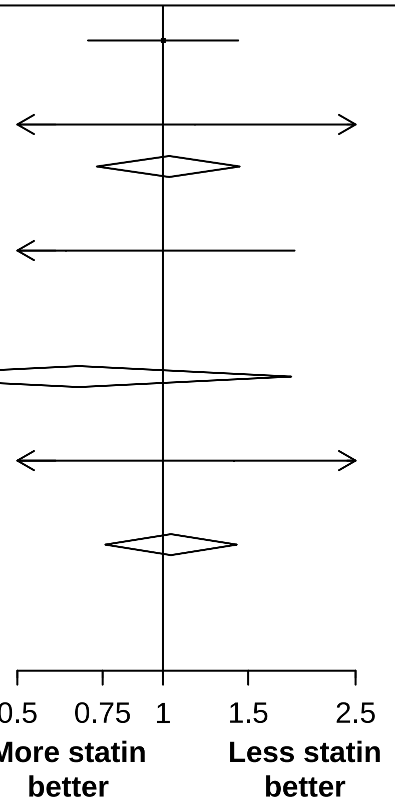

A patient may contribute to more than one row, but only once in each row.

* WBC=White blood cell count

** RBC=Red blood cell count

Webfigure 10a: Effect of more vs less intensive statin on RENAL AND URINARY, REPRODUCTIVE SYSTEM AND BREAST, MUSCULOSKELETAL AND CONNECTIVE TISSUE DISORDERS, AND GENERAL DISORDERS AND ADMINISTRATION SITE CONDITIONS listed in statin SmPCs, subdivided by category component parts

Endpoint	Events (% per annum)		Observed−Expected		Rate Ratio (95% CI)
	More statin (n=15390)	Less statin (n=15334)	O−E	Var(O−E)	
**Renal and urinary disorders**					
Acute kidney injury	58 (<0.1)	70 (<0.1)	−6.1	32.0	0.83 (0.58 − 1.17)
Dysuria	51 (<0.1)	38 (<0.1)	6.6	22.2	1.34 (0.89 − 2.03)
Haematuria	190 (0.3)	196 (0.3)	−3.1	96.5	0.97 (0.79 − 1.18)
Micturition disorder	314 (0.4)	261 (0.4)	26.7	143.7	1.20 (1.02 − 1.42)
Urinary composition alteration	81 (0.1)	78 (0.1)	1.5	39.7	1.04 (0.76 − 1.42)
**Reproductive system and breast disorders**					
Erectile and sexual dysfunction	325 (0.5)	282 (0.4)	21.7	151.7	1.15 (0.98 − 1.35)
Gynaecomastia	23 (<0.1)	12 (<0.1)	5.5	8.7	1.87 (0.97 − 3.63)
**Musculoskeletal and connective tissue disorders**					
Joint swelling or pain	1139 (1.6)	1191 (1.7)	−28.0	582.4	0.95 (0.88 − 1.03)
Non−specific muscle disorder	16 (<0.1)	8 (<0.1)	4.0	6.0	1.93 (0.87 − 4.30)
Tendon problems	185 (0.3)	197 (0.3)	−6.2	95.5	0.94 (0.77 − 1.14)
**General disorders and administration site conditions**					
Asthenia, fatigue and malaise	1157 (1.7)	1144 (1.6)	5.5	574.9	1.01 (0.93 − 1.10)
Oedema	763 (1.1)	799 (1.1)	−19.4	390.4	0.95 (0.86 − 1.05)
Pain	1887 (2.8)	1926 (2.8)	−26.0	952.6	0.97 (0.91 − 1.04)
Pyrexia	98 (0.1)	86 (0.1)	5.8	46.0	1.13 (0.85 − 1.51)

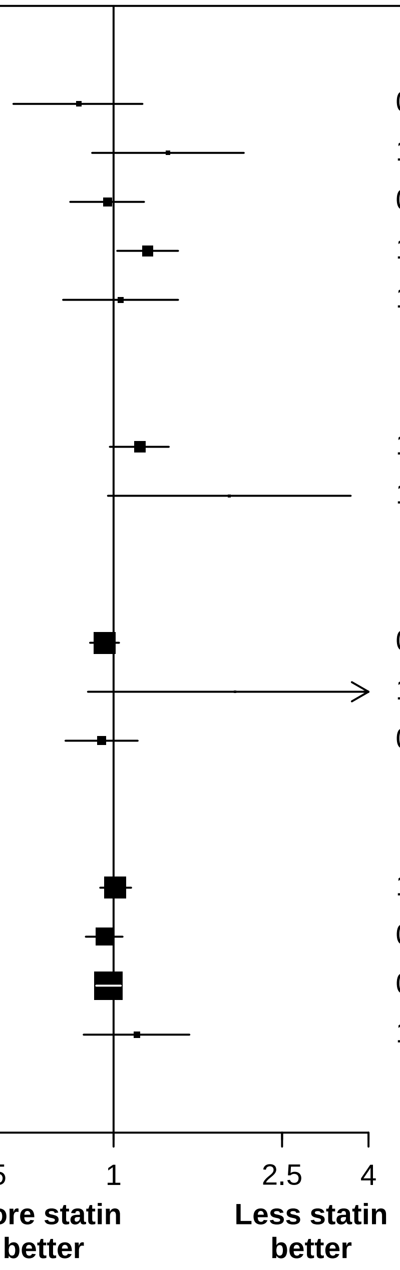

Webfigure 10b: Effect of more vs less intensive statin on NERVOUS SYSTEM, PSYCHIATRIC, EYE, AND EAR AND LABYRINTH DISORDERS listed in statin SmPCs, subdivided by category component parts

Endpoint	Events (% per annum)		Observed−Expected		Rate Ratio (95% CI)
	More statin (n=15390)	Less statin (n=15334)	O−E	Var(O−E)	
**Nervous system disorders**					
Cognitive or memory impairment	144 (0.2)	158 (0.2)	−7.2	75.5	0.91 (0.73 − 1.14)
Dementia including Alzheimer's	79 (0.1)	78 (0.1)	0.5	39.2	1.01 (0.74 − 1.38)
Dizziness	1143 (1.6)	1100 (1.6)	21.1	560.5	1.04 (0.96 − 1.13)
Headache	592 (0.8)	617 (0.9)	−14.2	302.1	0.95 (0.85 − 1.07)
Altered sensation	467 (0.7)	447 (0.6)	10.4	228.5	1.05 (0.92 − 1.19)
Peripheral or poly−neuropathy	100 (0.1)	114 (0.2)	−7.2	53.5	0.87 (0.67 − 1.14)
**Psychiatric disorders**					
Depression	567 (0.8)	589 (0.8)	−12.2	289.0	0.96 (0.85 − 1.08)
Sleep−related disorders	425 (0.6)	498 (0.7)	−38.0	230.7	0.85 (0.75 − 0.96)
**Eye disorders**					
Diplopia	16 (<0.1)	16 (<0.1)	0.0	8.0	1.00 (0.50 − 2.00)
Vision blurred	56 (<0.1)	47 (<0.1)	4.5	25.7	1.19 (0.81 − 1.75)
Vision impairment	123 (0.2)	136 (0.2)	−6.8	64.7	0.90 (0.71 − 1.15)
**Ear and labyrinth disorders**					
Deafness	44 (<0.1)	39 (<0.1)	2.5	20.7	1.13 (0.74 − 1.74)
Tinnitus	52 (<0.1)	62 (<0.1)	−5.0	28.5	0.84 (0.58 − 1.21)

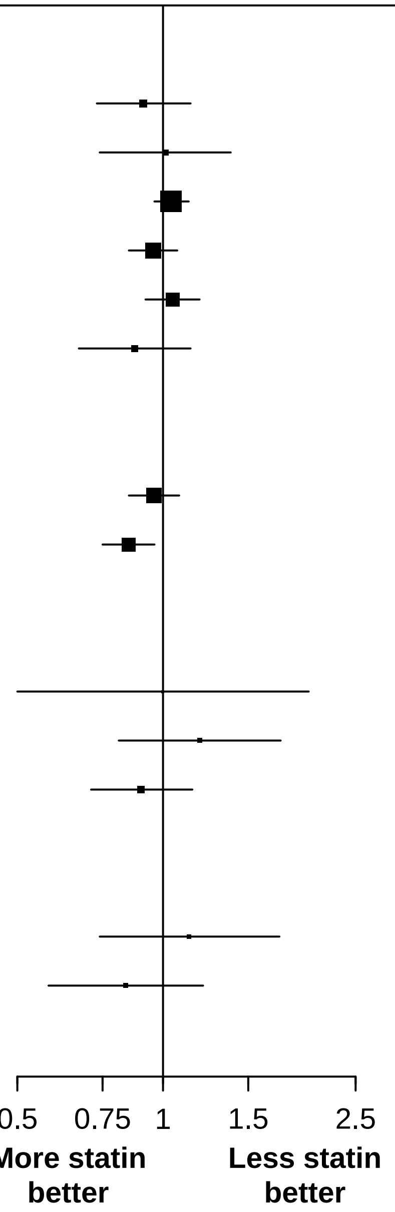

Webfigure 10c: Effect of more vs less intensive statin on BLOOD AND LYMPHATIC SYSTEM, IMMUNE RELATED, SKIN AND SUBCUTANEOUS TISSUE, AND RESPIRATORY, THORACIC AND MEDIASTINAL DISORDERS listed in statin SmPCs, subdivided by category component parts

Endpoint	Events (% per annum)		Observed−Expected		Rate Ratio (95% CI)
	More statin (n=15390)	Less statin (n=15334)	O−E	Var(O−E)	
**Blood and lymphatic system disorders**					
Anaemia	616 (0.9)	613 (0.9)	2.0	307.2	1.01 (0.90 − 1.13)
Eosinophilia	0 (0.0)	2 (<0.1)	−1.0	0.5	
Thrombocytopaenia	56 (<0.1)	35 (<0.1)	10.5	22.7	1.59 (1.05 − 2.39)
RBC sedimentation rate increased †	1 (<0.1)	4 (<0.1)	−1.5	1.2	0.30 (0.05 − 1.74)
**Immune related disorders**					
Anaphylactic reaction or hypersensitivity	161 (0.2)	139 (0.2)	10.9	75.0	1.16 (0.92 − 1.45)
Angioedema	43 (<0.1)	47 (<0.1)	−2.1	22.5	0.91 (0.60 − 1.38)
Immune conditions affecting muscle	36 (<0.1)	47 (<0.1)	−5.5	20.7	0.77 (0.50 − 1.18)
Lupus−like syndrome	7 (<0.1)	6 (<0.1)	0.5	3.2	1.17 (0.39 − 3.46)
Serious skin drug reactions	1 (<0.1)	0 (0.0)	0.5	0.2	
Vasculitis	5 (<0.1)	8 (<0.1)	−1.5	3.2	0.63 (0.21 − 1.86)
**Skin and subcutaneous tissue disorders**					
Hair disorder	37 (<0.1)	47 (<0.1)	−5.1	21.0	0.79 (0.51 − 1.20)
Dermatitis and eczema	143 (0.2)	143 (0.2)	−0.1	71.5	1.00 (0.79 − 1.26)
Rash or urticaria	589 (0.8)	591 (0.8)	−1.9	294.9	0.99 (0.89 − 1.11)
Pruritus	157 (0.2)	198 (0.3)	−20.9	88.7	0.79 (0.64 − 0.97)
Other skin condition	120 (0.2)	135 (0.2)	−7.5	63.7	0.89 (0.70 − 1.14)
Flushing	68 (<0.1)	82 (0.1)	−7.2	37.5	0.83 (0.60 − 1.14)
**Respiratory, thoracic and mediastinal disorders**					
Cough	523 (0.7)	514 (0.7)	4.4	259.2	1.02 (0.90 − 1.15)
Dyspnoea	1030 (1.5)	1093 (1.6)	−35.8	530.6	0.93 (0.86 − 1.02)
Epistaxis	181 (0.2)	169 (0.2)	5.8	87.5	1.07 (0.87 − 1.32)
Interstitial lung disease	32 (<0.1)	33 (<0.1)	−0.5	16.2	0.97 (0.60 − 1.57)
Laryngeal pain	89 (0.1)	93 (0.1)	−2.0	45.5	0.96 (0.72 − 1.28)
Nasopharyngitis	403 (0.6)	419 (0.6)	−8.1	205.5	0.96 (0.84 − 1.10)

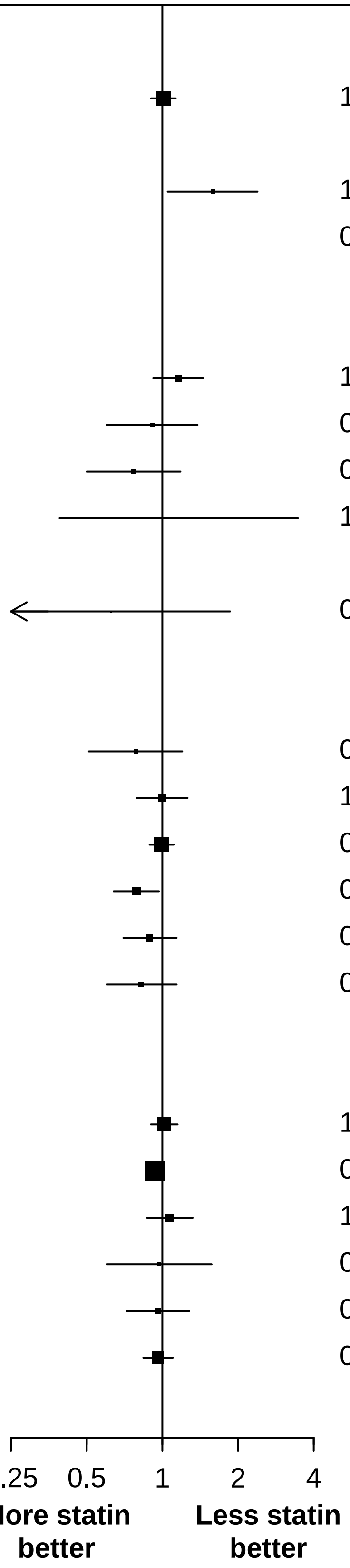

† RBC=Red blood cell

RRs are only plotted for outcomes with at least 10 events, though all outcomes contribute to the shown subtotals and totals

Webfigure 11: Effect of statin vs placebo on ANY MUSCLE PAIN OR WEAKNESS and on NEW−ONSET DIABETES by statin intensity

	Events (% per annum)		Observed−Expected		Rate Ratio (95% CI)
	Statin	Placebo	O−E	Var(O−E)	
**Any muscle pain or weakness [1]**					
Low− or moderate−intensity statin	14359 / 51288 (7.7)	14147 / 51207 (7.6)	178.6	7099.6	1.03 (1.00 − 1.05)
High−intensity statin	2476 / 10740 (10.3)	2299 / 10705 (9.4)	104.5	1193.1	1.09 (1.03 − 1.16)
**New−onset diabetes [2]**					
Low− or moderate−intensity statin	2420 / 39179 (1.3)	2214 / 39266 (1.2)	106.8	1156.8	1.10 (1.04 − 1.16)
High−intensity statin	1221 / 9935 (4.8)	905 / 9859 (3.5)	163.9	530.8	1.36 (1.25 − 1.48)

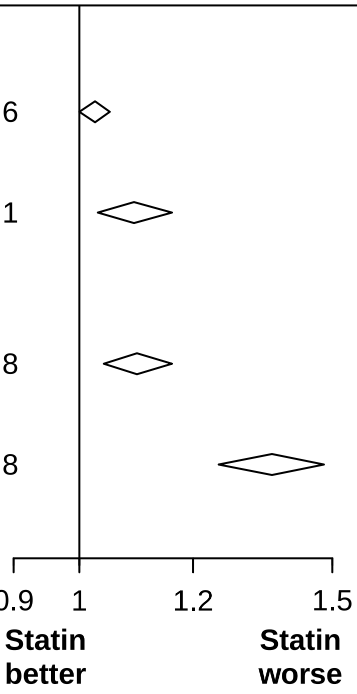

Results shown are as previously reported.
Denominators for the outcome of muscle pain or weakness[1] are all participants randomised into the 19 trials of statin therapy vs placebo {https://doi.org/10.1016/S0140−6736(22)01545−8}.
Denominators for the outcome of new−onset diabetes[2] are participants in the relevant group of trials without known diabetes at randomisation {https://doi.org/10.1016/S2213−8587(24)00040−8}.
